# Imaging Dihydrogen Bond‐Driven Assembly of Borazine on Au(111)

**DOI:** 10.1002/chem.202403996

**Published:** 2024-12-06

**Authors:** Matthias Zeilerbauer, Marco Thaler, Barbara Obwaller, Milan Ončák, Laerte L. Patera

**Affiliations:** ^1^ Department of Physical Chemistry University of Innsbruck 6020 Innsbruck Austria; ^2^ Department of Ion Physics and Applied Physics University of Innsbruck 6020 Innsbruck Austria

**Keywords:** Borazine, Dihydrogen bonding, Scanning tunnelling microscopy, Density functional theory, Molecular assembly

## Abstract

Dihydrogen bonding (DHB) is a peculiar type of attractive interaction occurring between a partially positively charged hydrogen atom and a partially negatively charged hydrogen atom. Borazine represents a prototypical molecule exhibiting dihydrogen bonding in both gas phase, as well as in its crystalline form. For borazine assemblies on solid surfaces, a direct observation and characterization of dihydrogen bonding has remained elusive, possibly due to an intricate interplay of substrate‐molecule and intermolecular interactions. Here we present evidence of dihydrogen bonding occurring in borazine assemblies on a Au(111) surface. By means of low‐temperature scanning tunneling microscopy, we unveiled distinct configurations, exhibiting single and double dihydrogen bonding. Density functional theory calculations elucidate the interplay between substrate adsorption and intermolecular interactions to stabilize the formation of borazine dimers on Au(111). The dimers constitute the building blocks for the formation of larger assemblies.

## Graphical abstract

This study presents the first observation of dihydrogen bonding (DHB) in borazine assemblies on a Au(111) surface using low‐temperature scanning tunneling microscopy. Density functional theory calculations elucidate the stabilization mechanisms behind single and double DHB configurations, revealing the crucial role of DHBs in the self‐assembly processes of borazine on metal surfaces.

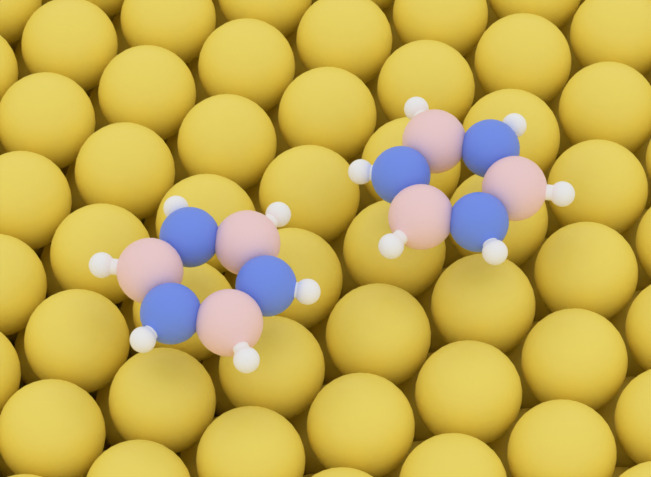

## Introduction

Dihydrogen bonding (DHB) emerged as a fascinating area of study within chemical interactions, showcasing its influence on diverse aspects of chemistry ranging from reaction mechanisms to crystal packing.[^1^, ^2^] In conventional hydrogen bonding (HB) the attractive interactions are caused by a combination of electrostatic contributions, charge transfer and dispersion forces between the hydrogen bond donor and acceptor.^[3]^ The same concept can be applied to dihydrogen bonding, where the interaction arises between a protonic hydrogen and a hydridic hydrogen.^[4]^ Specifically, the bonding is of the type D−H⋅⋅⋅H−A, with D being a typical hydrogen donor (as O, N, F), and A being a hydrogen acceptor, often a transition metal (like Ir or Fe) or B.

First experimental evidences of DHB trace back to the late 1960s, where interactions between hydrogen atoms in BH_3_NH_3_ have been reported in infrared (IR) solution spectra.[^5^, ^6^] The existence of DHBs has been highlighted also by striking differences in properties such as melting points, as evidenced by the substantial difference between BH_3_NH_3_ and the isoelectronic ethane molecule.^[7]^ Energy gains associated with DHB formation, such as the 6.1 kcal/mol per bond observed in BH_3_NH_3_ dimers, underscore the significance of these interactions.^[7]^ Criteria for identifying DHB include destabilization of the protic hydrogen atom upon complex formation^[7]^ and their respective IR and NMR spectral changes.^[8]^ In contrast to isotropic van der Waals interactions, DHB also possesses strong directional preference, i. e. D−H⋅⋅⋅H−A bond angles usually between 110° and 180°.^[3]^ Density Functional Theory (DFT) calculations have shown that in a dimer structure of BH_3_NH_3_ all B−H and N−H bonds involved in DHBs lengthen, while bonds not directly participating in the B−H⋅⋅⋅H−N bond shorten slightly.^[7]^ The formation of DHB has been shown to influence reaction mechanisms, as exemplified by the synthesis of diammoniate of diborane where DHB stabilizes intermediates.^[9]^ Moreover, DHB plays crucial roles in crystal packing, hydrogen‐storage materials, as well as organometallic reaction mechanisms.[^1^, ^10^] Intriguingly, the unique ability of DHBs to lose H_2_ in the solid state, trading weak H^δ−^⋅⋅⋅H^δ+^ interactions for strong covalent bonds, promises new routes to the rational assembly of ordered, extended covalent materials.[^11^, ^12^]

**Figure 1:**
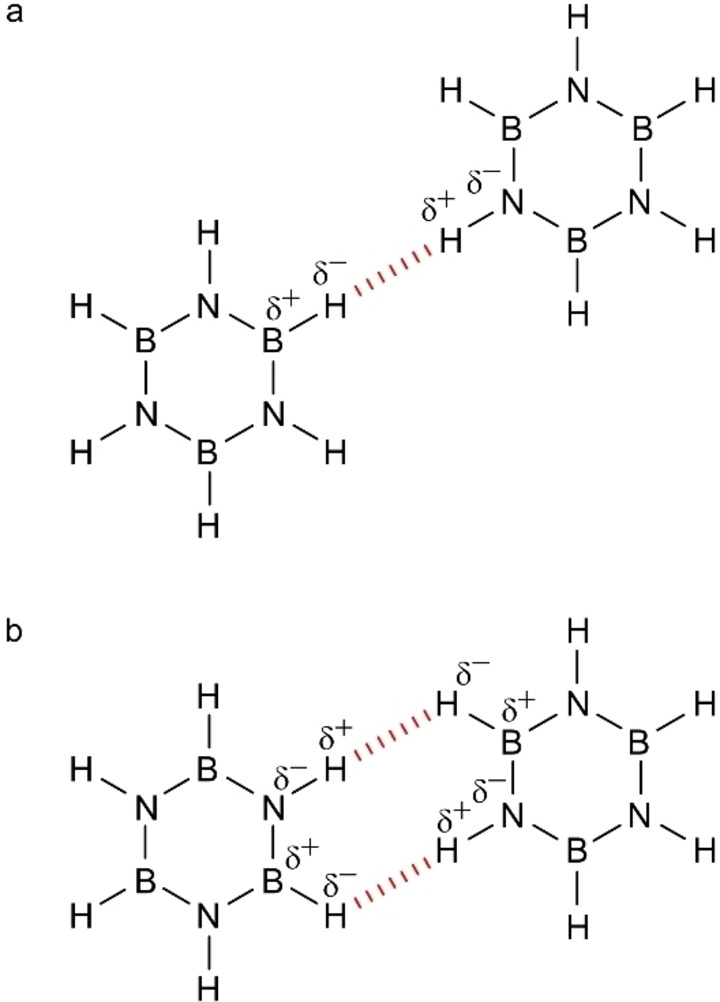
Schematic illustrations of borazine dimers forming (a) one and (b) two dihydrogen bonds, respectively. The non‐covalent interactions are highlighted in red.

Borazine is considered as the inorganic analogue of benzene.[^13^, ^14^, ^15^] However, the difference in electronegativity between N and B hinders a complete electron delocalization and induces a polarization of the N−H and B−H bonds, making borazine a promising candidate for the formation of DHB.^[10]^ For a coplanar borazine dimer, two distinct bonding motifs can be foreseen, each characterized by either one or two dihydrogen bonds (DHBs), as shown in Figure 1. Matrix isolation infrared spectroscopy and *ab‐initio* quantum chemical calculations of borazine dimers reported the formation of several pair configurations, with distances between the two interacting hydrogens down to about 2.1 Å.^[16]^ In the crystalline structure, the dihydrogen bond length is prolonged to about 2.5 Å.^[17]^

Borazine has been widely used in the field of surface science for the synthesis of high‐quality monolayers of hexagonal boron nitride on metal surfaces.[^18^, ^19^, ^20^, ^21^, ^22^, ^23^, ^24^, ^25^] Recently, real‐space imaging of borazine assemblies on a Ag(111) surface has been reported for the first time by means of low‐temperature scanning tunneling microscopy (LT‐STM).^[26]^ Although specific building blocks have been identified within the monolayer structure, no clear evidence of significant intermolecular interaction has been reported. Therefore, a direct observation and characterization of DHB in borazine assemblies on surfaces has remained elusive, possibly due to an intricate interplay of substrate‐molecule and intermolecular interactions.[^27^, ^28^]

Here, we provide direct evidence of DHB occurring for borazine molecules adsorbed on a Au(111) surface. LT‐STM images reveal the presence of different configurations characterized by single and double DHB, which drives the formation of borazine dimers and clusters. DFT calculations elucidate the contributions of substrate‐molecule interactions and intermolecular forces, providing insights into the on‐surface formation of DHB‐driven molecular assembly.

## Results and Discussion

Borazine molecules have been deposited on the Au(111) substrate kept at a temperature *T*≈22 K inside the microscope and subsequently cooled down to *T*≈8.5 K for characterization (see Supporting Information, SI). The Au(111) surface has been chosen because of its inertness,^[29]^ which facilitates the investigation of fundamental intermolecular interactions in borazine assemblies. The triangular lattice of the substrate matches with the *D*_3h_ symmetry of the borazine, being shown to stabilize small molecules upon STM imaging.^[30]^ Dosing on the cold substrate (*T*≈22 K) ensures non‐dissociative adsorption.[^19^, ^26^] The borazine molecules were deposited at low coverages, with an average concentration of approximately one molecule per 40 nm^2^. This approach was taken to prevent the formation of dense assembly structures, which could overshadow the intermolecular interactions of interest.[^22^, ^31^, ^32^, ^33^]

**Figure 2:**
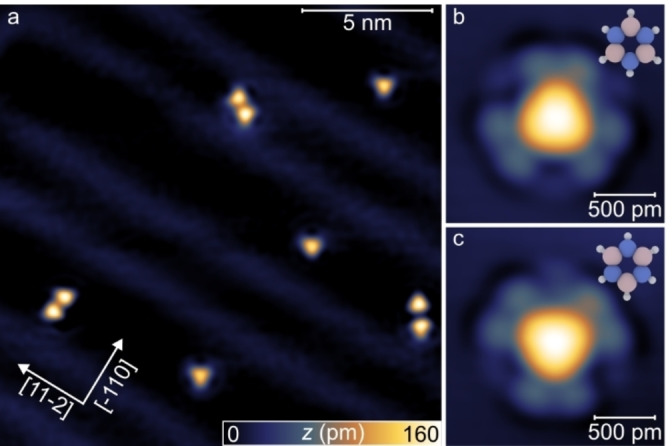
Borazine on Au(111). (a) Overview STM image of borazine on Au(111) showing monomers and dimers (*V*=200 mV; *I*=45 pA). (b,c) High‐resolution STM images of borazine monomers (*V*=20 mV; *I*=20 pA). Ball models of the molecule are superimposed, showing the corresponding molecular orientations. Pink, blue and white spheres represent boron, nitrogen and hydrogen, respectively.

Figure 2a shows an STM image acquired upon borazine dosing on the Au(111) surface, revealing the presence of individual molecules, as well as small aggregates (see also Figure S1a). No evident decoration of the step‐edge sites is observed (Figure S1b), indicating that the substrate temperature during the deposition (*T*≈22 K) limits the molecular diffusion, enabling kinetic trapping of weakly‐bound configurations.^[34]^ In high‐resolution images (Figure 2b), individual molecules appear as triangular protrusions, resembling the STM contrast reported for borazine on Ag(111).^[26]^ The corners of the triangular feature allow locating the positions of the B atoms within each molecule (see models in Figure 2b, c).^[26]^ Each molecule typically displays a rim composed of several protrusions of lower apparent height, resembling a solvation shell. The latter can be assigned to H_2_ molecules, which result from the partial decomposition of borazine in the gas phase (see Figure S2).[^19^, ^35^] A direct identification of the small protrusion as individual hydrogen molecules has been obtained through inelastic electron tunneling spectroscopy (IETS, see Figure S3). In STM images, borazine molecules are found to exhibit two distinct orientations rotated by 180°, indicating a non‐negligible interaction with the symmetry‐matched surface (Figure 2b, c).^[26]^

To elucidate the adsorption behavior of borazine on the Au(111) surface, we performed DFT calculations using the PBE functional and the projector‐augmented wave (PAW) method.^[36]^ The simulations employed periodic boundary conditions, an energy cut–off of 350 eV, and a model comprising five layers of Au to represent the surface. Additionally, we included the vdW^surf^ dispersion correction.^[37]^ The calculations were performed in the VASP package, version 6.4.2 (see the SI for details).[^38^, ^39^] The calculations reveal that individual borazine molecules prefer to adsorb with nitrogen atoms in *top* position, and the boron atoms either in *fcc* or *hcp* positions (Figure S4). Their antiparallel alignment is consistent with the azimuthal orientations observed in the STM images (Figure 2b, c). Both *fcc* and *hcp* conformations have nearly the same adsorption energy of ~0.75 eV, slightly lower compared to the borazine adsorption energy calculated for the Ag(111) surface, 0.8–0.9 eV.^[26]^ Borazine is adsorbed on Au(111) at a height of ~3.1 Å above the surface in a flat conformation.

**Figure 3:**
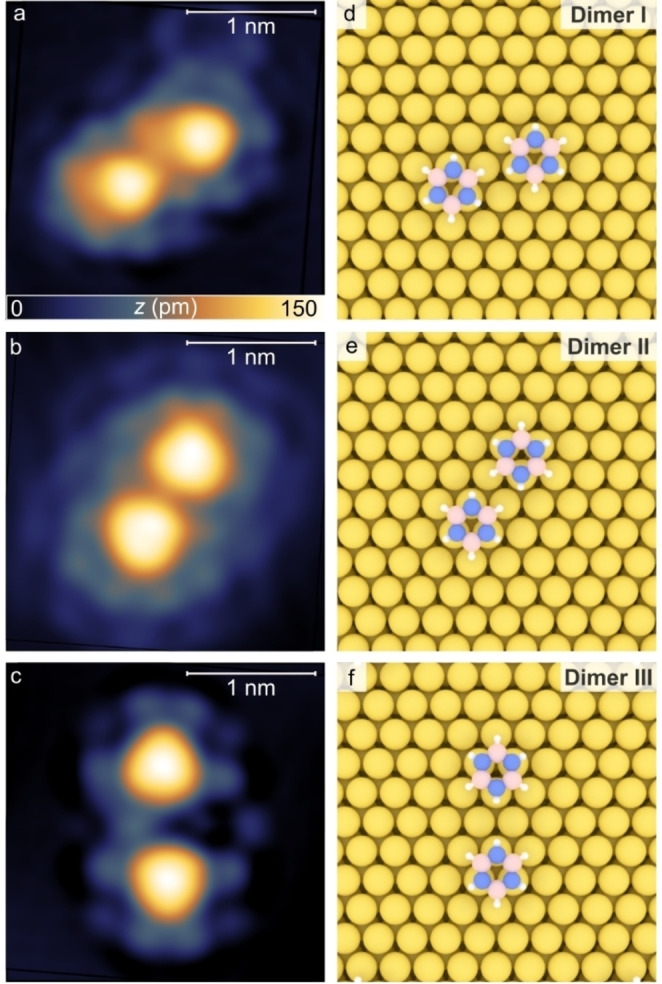
Borazine dimers on Au(111). (a–c) STM images of borazine dimers (a) *V*=50 mV; *I*=30 pA), (b) (*V*=30 mV; *I*=30 pA) (c) (*V*=15 mV, *I*=15 pA). (d–f) DFT optimized structures of the molecular dimers. Pink, blue, white and yellow spheres represent boron, nitrogen, hydrogen and gold respectively.

Having identified the adsorption structures of individual borazine molecules on Au(111), we proceeded with the investigation of the dimer structures. Figure 3a–c show STM images of the dimer configurations. In Figure 3a (dimer I), the molecules in the dimer have a distance between the centroids (*d*_cc_) of 7.0± 0.5 Å (see Table 1) and both possess the same azimuthal orientation. A second structure (dimer II, Figure 3b) displays a *d*_cc_ =6.8±0.5Å , but opposite relative orientations of the molecules. The structure in Figure 3c (dimer III) is composed of two borazine molecules exhibiting *d*_cc_ = 8.5± 0.5 Å and opposite azimuthal orientation. Figure 3d–f show models of the corresponding DFT‐optimized structures for the three dimer configurations. Again, the most stable structures show nitrogen atoms in *top* positions.

**Table 1:** Borazine dimers on Au(111). Experimental and calculated centroid distances (*d*_cc_), calculated H−H distance (*d*_HH_) and calculated dimer formation energies (Δ*E*).

	*d*_cc_ (exp) [Å]	*d*_cc_ (calc) [Å]	*d*_HH_ (calc) [Å]	Δ*E* [eV]
Dimer I	7.0±0.5	7.3	2.4	−0.02
Dimer II	6.8±0.5	6.9	2.5 (2x)	−0.04
Dimer III	8.5±0.5	8.4	3.5	0.02

Notably, short H−H lengths (*d*_HH_) are observed in dimers I and II (see Table 1). Therefore, it can be concluded that dimers I and II shown in Figure 3a, b may result from DHB between one and two pairs of interacting hydrogens, respectively. Instead for dimer III, the repulsive force resulting from the facing N−H groups implies that no bonding occurs for this dimer.

According to the DFT calculations, the *d*_HH_ for dimer I and II is 2.4–2.5 Å respectively (Table 1), being longer than those reported for gas‐phase dimers,^[16]^ but close to the values in the crystal.^[10]^ The *d*_HH_ values are the result of an interplay between the intermolecular interactions and the molecule‐substrate‐interaction. For both dimer I and II, shorter values of *d*_HH_ would result in the displacement of the molecule out from the preferred adsorption configuration, being energetically unfavored. The formation energies indicate that dimers I and II are more stable than two independently adsorbed borazine molecules by 0.02 eV and 0.04 eV, respectively (see Table 1). The calculated *d*_cc_ values show strong agreement with the experimental data (see Table 1) across all dimer configurations (see SI and Table 1). Co‐adsorbed H_2_ molecules have no influence on the dimer stability, as shown in Figure S5 for dimer II.

While for dimer I and II the STM images (Figure 3a, b) match well with the relaxed structures (Figure 3d, e), a lateral offset between the two borazine molecules can be observed in the STM image of dimer III (Figure 3c), which is not perfectly aligned with the calculated model (Figure 3f). This discrepancy can be attributed to the asymmetric distribution of adsorbed H₂ molecules around dimer III. Given that dimer III is non‐bonding in nature, it may in fact be more susceptible to perturbations from surrounding adsorbates, leading to the lateral offset observed in the STM images. This asymmetry in the local environment could influence the molecular orientation, resulting in the subtle misalignment.

To assess whether borazine dimers are already present in the gas phase during dosing or whether they form after adsorption on the Au(111) surface, we refer to the literature and compare their behavior to that of H_2_O. At ambient conditions, gas‐phase water consists mainly of H_2_O monomers with only a small fraction (about 0.1 %) of the water molecules being present as hydrogen‐bonded dimers.^[40]^ Given the dosing conditions (*T*=300 K), it is reasonable to assume that the gas phase of borazine primarily consists of monomers. This assumption is supported by the similar binding energies of borazine dimers (120 meV for a dimer with two DHBs)^[16]^ and H₂O dimers (140 meV).^[41]^ Our experimental observations also reveal a higher prevalence of monomers on the surface (see Figure S1a). Therefore, we attribute the formation of dimers and small clusters mainly to monomer adsorption on the Au(111) surface from the gas phase, followed by surface diffusion and aggregation. It is important to note that the deposition on the cold substrate (*T*≈22 K) results in a kinetic trapping of borazine assemblies, yielding the formation of metastable structures (dimer I, Figure 3a, d),[^34^, ^42^] as well as in non‐interacting configurations (dimer III, Figure 3c, f).

**Figure 4:**
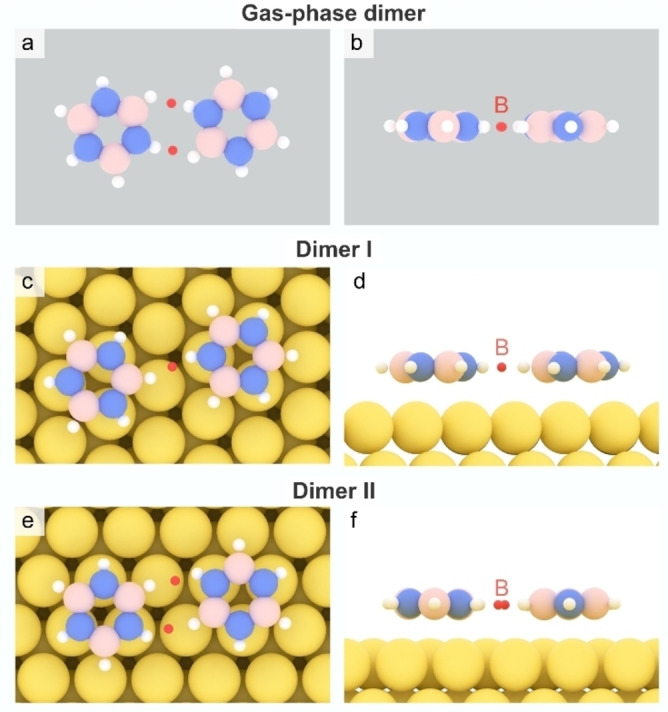
Calculated bond critical points (B, red) for the gas‐phase dimer (a, b) and for dimer I (c, d) and dimer II (e, f) adsorbed on the Au(111) surface. Panels a, c, and e show the top views, while b, d, and f depict the side views, respectively.

We performed bond critical point (BCP) analyses for borazine dimers within the Critic2 software[^43^, ^44^] to substantiate the presence of dihydrogen bonding in dimer I and II. BCPs are first‐order saddle points of the electron density, signifying a region that is characteristic of a bonding interaction.^[45]^ Their presence and properties, such as electron density values, serve as quantitative indicators of DHB by confirming subtle interactions between hydrogen atoms. In the gas‐phase dimer, two BCPs are identified between hydrogen atoms (see Figure 4a, b), indicating the presence of intermolecular interactions characteristic of DHB.^[16]^ The corresponding electron density at the BCPs are ~10^−2^ a.u., approximately one order of magnitude lower than that of covalent bonds and comparable to the value observed for the hydrogen bond in a water dimer (~3×10^−2^ a.u., see also Figure S8). Similar BCPs, although with the density of ~4×10^−3^ a.u., were also observed for dimers I and II, suggesting the persistence of DHB upon adsorption on the Au(111) surface (Figure 4c–f). The identification of the critical points reinforces the role of dihydrogen bonding in stabilizing the borazine assemblies.

Electron density calculations for the borazine dimers reveal higher electron density around the hydrogen atoms implicated in DHB (Figure S6), highlighting specific charge arrangements that persist even upon adsorption on the Au(111) surface and reinforcing the presence of DHB.

Furthermore, DFT calculations reveal that the bond lengths for N−H and B−H participating in DHB increase. This phenomenon is observed in both the gas‐phase dimer and in dimers I and II (Figure S7). In contrast, no deviations in bond lengths are observed in dimer III compared to those found in a borazine monomer adsorbed on Au(111).

Notably, borazine pairs with antiparallel alignment and short intermolecular distance (*d*_cc_=6.4 Å) have been recently observed in dense assembly of borazine on Ag(111).^[26]^ This configuration closely resembles the dimer II (Figure 3b, e), suggesting that DHB may play a crucial role in driving the self‐assembly of borazine into two‐dimensional crystalline networks.

Overall, DHB represents the driving force for the on‐surface formation of borazine dimers on Au(111). This highlights the role of electronegativity of heteroatoms in steering intramolecular charge accumulation, driving non‐covalent intermolecular bonds at surfaces.[^46^, ^47^, ^48^] A similar effect has been recently reported for the case of a mixed layer of benzene and hexafluorobenzene, where the presence of a π hole in fluorinated anthracene gives rise to specific electrostatic interactions.^[49]^ Benzene molecules have been reported to form small clusters once deposited on Cu(111), being stabilized by the electronic modulation of the surface density of states close to the step edges.^[50]^ Instead, borazine dimers and clusters are typically found on flat terraces (in both *fcc* and *hcp* regions) of the Au(111) surface (see Figures 1a and S1b), further pointing at intermolecular interactions as the driving force for the formation of the observed assemblies on Au(111).

**Figure 5:**
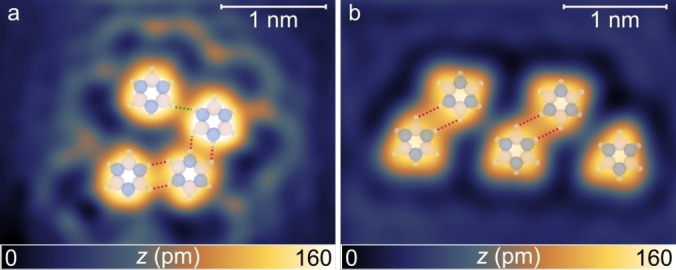
Borazine clusters on Au(111). (a) *V*=30 mV; *I*=20 pA), (b) (*V*=50 mV; *I*=50 pA). Ball‐and‐stick models are superimposed to indicate the positions and mutual orientations of individual borazine molecules. In these models, pink, blue, and white spheres represent boron, nitrogen, and hydrogen atoms, respectively. Dihydrogen bonds (DHBs) are highlighted in green and red, representing dimer I and dimer II types, respectively.

The DHB motifs identified in Figure 3 constitute the building blocks for larger borazine assemblies. Figure 5a shows a tetramer, consisting of three molecules linked by the dimer II motif, with a fourth molecule attached via a dimer I configuration. In Figure 5b a cluster composed of five borazine molecules can be observed, in which two dimers of type II are evident. No DHB is present either between the two dimers or between the central dimer and the adjacent single molecule on the right, closely resembling the dimer III configuration.

## Conclusions

This study provides the first evidence of dihydrogen bonding in borazine assemblies on a Au(111) surface. Through low‐temperature scanning tunneling microscopy and density functional theory calculations, we elucidated the formation of borazine dimers stabilized by single and double DHBs. The interplay between substrate adsorption and intermolecular interactions is crucial in stabilizing these configurations, which serve as the fundamental building blocks for larger assemblies. The results highlight the significant role of DHBs in the self‐assembly processes of borazine on metal surfaces, offering new insights into the potential of DHBs to drive molecular organization.

## Conflict of Interests

The authors declare no conflict of interest.

1

## Supporting information

As a service to our authors and readers, this journal provides supporting information supplied by the authors. Such materials are peer reviewed and may be re‐organized for online delivery, but are not copy‐edited or typeset. Technical support issues arising from supporting information (other than missing files) should be addressed to the authors.

Supporting Information

## Data Availability

The data that support the findings of this study are openly available in researchdata.uibk.ac.at at https://doi.org/10.48323/t7c0m‐26418, reference number 24082024.
