# Supplementary material for: Imaging Dihydrogen Bond‐Driven Assembly of Borazine on Au(111)
Source: Chemistry. 2024 Dec 6;31(5):e202403996. doi: 10.1002/chem.202403996 (PMC11753383; doi:10.1002/chem.202403996)
Supplement: Supplementary file 1 — Supporting Information [file CHEM-31-e202403996-s001.pdf]

Chemistry–A European Journal

Supporting Information

Imaging Dihydrogen Bond-Driven Assembly of Borazine on Au(111)

Matthias Zeilerbauer, Marco Thaler, Barbara Obwaller, Milan Ončák,* and Laerte L. Patera*

Supporting Information
©Wiley-VCH 2021
69451 Weinheim, Germany

Imaging Dihydrogen Bond-Driven Assembly of Borazine on Au(111)

Matthias Zeilerbauer,[a] Marco Thaler,[a] Barbara Obwaller,[b] Milan Ončák,*[b] and Laerte L. Patera*[a]

[a] M. Zeilerbauer, M. Thaler, and Prof. Dr. L.L. Patera
Department of Physical Chemistry
University of Innsbruck
6020 Innsbruck (Austria)
E-mail: Laerte.Patera@uibk.ac.at

[b] B. Obwaller, and Prof. Dr. M. Ončák
Department of Ion Physics and Applied Physics
University of Innsbruck
6020 Innsbruck (Austria)
E-mail: Milan.Oncak@uibk.ac.at

Experimental Procedures

STM/STS experiments have been conducted using a commercial low-temperature STM (Infinity SPM, Scienta Omicron GmbH) in ultrahigh vacuum ($p \approx 5 \times 10^{-10}$ mbar) and at a temperature of ≈ 8.5 K. Bias voltage (V) is given as sample bias with respect to the tip. dI/dV spectroscopy has been performed using lock-in detection of the tunneling current I by adding a sinusoidal voltage modulation (V_{mod} = 20 mV_{rms}) at 759 Hz to the sample bias voltage V. The d^2I/dV^2 spectra have been obtained by numerically differentiation of the dI/dV ones. STM feedback was used for adjusting the tip-sample distance at each location.
STM images have been processed with Gwyddion.[1] A 2D Fast Fourier Transform (FFT) filter to remove high-frequency noise components.

Mass spectra were measured by a quadrupole mass spectrometer (PrismaPro QMG 250, Pfeiffer Vacuum GmbH).

Borazine was purchased from Katchem. In order to minimize the polymerization of borazine to polyborazylene, borazine was not stored as a liquid, but instead a minican was filled with borazine vapor (at room temperature the vapor pressure of borazine is approximately 270 mbar). The minican was connected to a leak valve by a stainless-steel pipe. Borazine is known to decompose into diborane at room temperature. To avoid contamination with diborane, borazine was cleaned using a modified version of the borazine purification protocol developed by Cuxart et al.[2] The procedure for purification was as follows:

- Pump gas line for 2 minutes with a scroll pump (p = 3×10^{-2} mbar).
- Install cooling trap filled with a mixture of Ethanol and LN_2 (constant temperature of the mixture is -116°C) and wait 5 minutes for line to cool down.
- Fill line with borazine from the minican for 20 seconds, borazine will condense in the line.
- Pump down line for 2 minutes with scroll pump in order to remove diborane.
- Let line (and borazine) warm to room temperature, the line is now filled with pure borazine.

Borazine molecules have been deposited on the Au(111) substrate kept at a temperature T ≈ 22 K inside the microscope ($p \approx 5 \times 10^{-7}$ mbar, t = 10 minutes) and subsequently cooled down to ≈ 8.5 K for characterization.

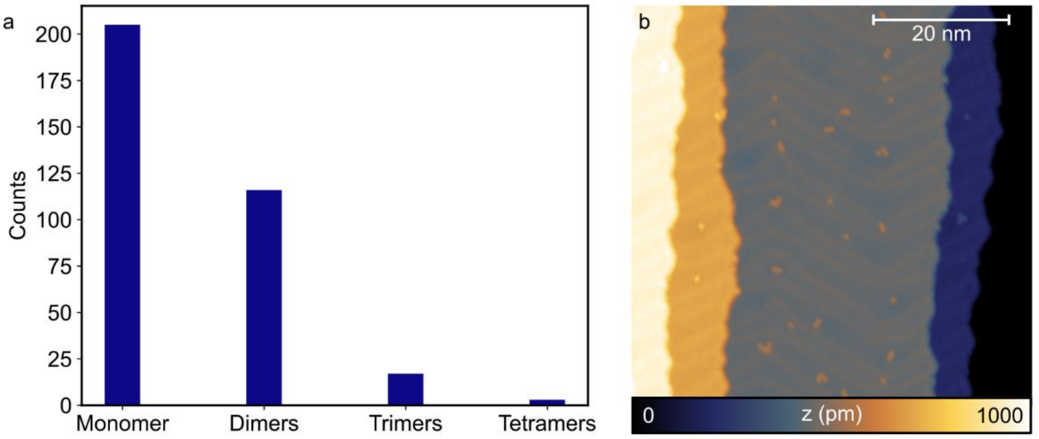

Figure S1. (a) Histogram of monomers, dimers, trimers and tetramers identified upon dosing borazine on the Au(111) kept at T ≈ 22 K. Out of 14 total dimers resolved with sufficient resolution, 2 correspond to dimer I, 9 to dimer II, and 3 to dimer III. (b) Large scale STM image showing several step edges of the gold surfaces. Individual molecules, as well as small clusters can be observed on the terraces, while no evident decoration of the step edge site is visible.

Details on the analysis of the STM images of the dimers

A total of 12 dimers were selected for determining the centroid distances (d_{cc}) of the three dimer structures. While over 100 dimers were imaged (see Figure SI1a), the analysis focused on this subset because these images exhibited the high resolution required to precisely and reliably identify the positions of individual molecules.
The following procedure was applied to all of the detail images:

1. Scaled models of borazine have been superimposed to the high-resolution STM images of the dimers, aligning the bright protrusions with the positions of boron atoms.
2. Center-to-center distances in the models have been measured.

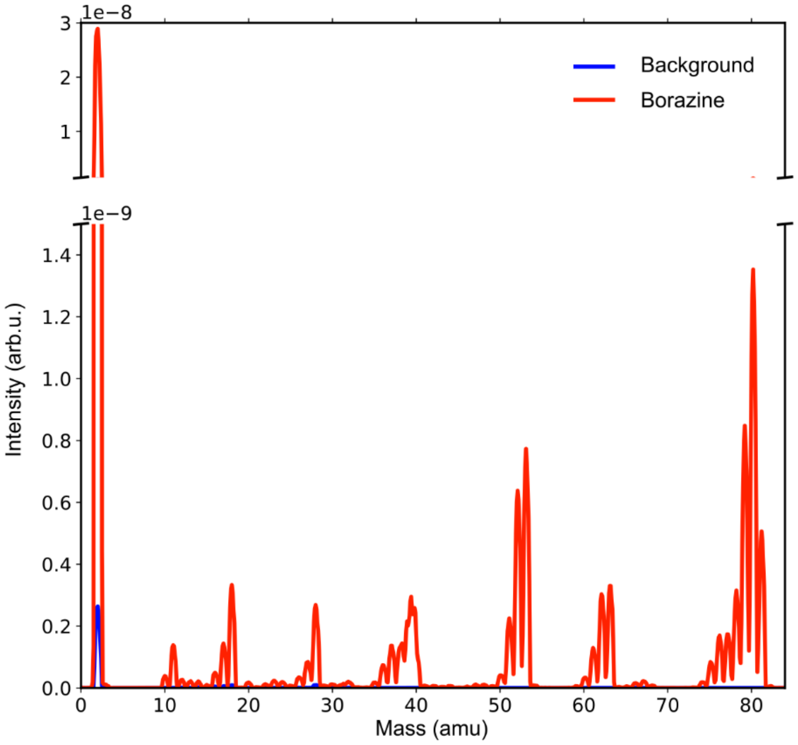

Figure S2. Mass spectra of the chamber background and the purified borazine. The latter has been measured at a partial pressure $p = 5\times10^{-7}$ mbar.

Borazine-H_2 structures

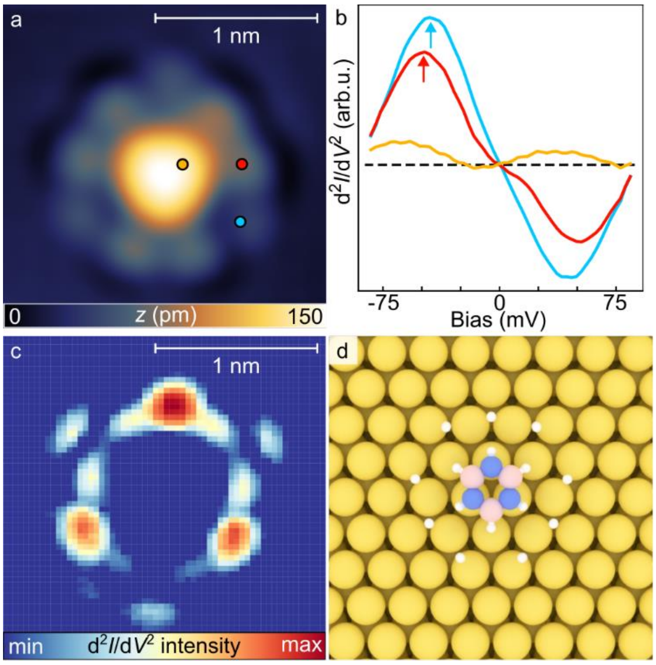

Figure S3. (a) STM image of an individual borazine surrounded by H_2 molecules (a, V = 100 mV; I = 200 pA), (b) IET spectra recorded at the positions marked in (a). The zero-signal level is indicated by a dashed line. (c) IETS intensity map. STM feedback was used for adjusting the tip-sample distance at each pixel. A slight image distortion is given due to residual drift. (d) Relaxed geometry of borazine with nine H_2. Pink, blue and white spheres represent boron, nitrogen and hydrogen, respectively.

Figure S3a shows an STM image of a borazine molecule surrounded by a rim of several protrusions. These additional lobes are attributed to adsorbed H_2, arising from the deposition process (see Figure S2). To unambiguously assign those features to H_2 we performed inelastic electron tunneling spectroscopy (IETS) for a borazine monomer. Spatially resolved IET spectra are shown in Figure S3b. While the spectrum acquired above the center of the borazine is featureless, the line shapes of the d^2I/dV^2 spectra on the rim are

characterized by an asymmetric dip. Similar spectral features have been reported for H_2/Au(110).[3] Hydrogen vibrational motion bouncing between the tip and substrate ($\nu = 0 \rightarrow 1$) has been identified around 11.4 mV on a clean Au(110) surface, while rotational excitation of para H_2 ($j = 0 \rightarrow 2$) gives rise to a feature at 42.0 mV. The IETS excitation energies have been shown to increase upon tip approach towards the surface.[3–7] Further changes of the excitation energies occur due to the spatial distribution of the interaction potential within an adsorbed molecule.[8] As the vibrational excitation is typically characterized by a larger intensity in the IET spectra compared to the rotational one, the features observed in Figure S3b are attributed to H_2 vibrations. We performed d^2I/dV^2 spectroscopic mapping, acquiring a grid of 64 × 64 spectra over an individual borazine molecule surrounded by adsorbed H_2. Figure S3c shows a map of the IETS intensities extracted from each spectrum. Notably, as no feature of an IET excitation is observed upon acquisition of a spectrum above the borazine, we conclude that for this tip termination the H_2 is not constantly trapped in the junction.[3,7] Therefore, the corresponding IETS map allows precisely locating the adsorption sites of the H_2 molecules around the borazine.

Three main features are located in front of the N-H bonds, while groups of three protrusions are located nearby the B-H one. Figure S3d shows the DFT relaxed structure of a borazine monomer surrounded by nine hydrogen molecules. The adsorption sites of the H_2 molecules match the position of the main features in IETS map (Figure S3c). The adsorption energy is about 0.09 eV per H_2 molecule.

Computational details

The calculations were performed on a surface Au(111) model including five Au layers, with the initial closest Au–Au separation of 2.884 Å, each Au layer containing 64 Au atoms (see Figure S4). During the course of optimization, the three lower layers were kept fixed to mimic interactions with bulk. In the *z* direction, the size of the cell was 30 Å, thus providing more than 15 Å of a vacuum layer between periodic images in this direction. We performed calculations employing density functional theory (DFT) with the PBE functional and the energy cut-off of 350 eV. To obtain more precise energies, the final structures were single-point recalculated using the cut-off of 500 eV, leading to a very slight decrease in adsorption energies below 2%. The smearing method of Methfessel-Paxton, second order, was employed. Only the gamma point was considered in the reciprocal space. Empirical dispersion was included employing the vdW^{surf} correction.[9] The energy convergence criterion was set to 10^{-6} eV for stopping the electronic self-consistent loop and 10^{-5} eV for structure optimization. We sampled various positions of the borazine monomer and dimer on the Au(111) surface, i.e., *fcc*, *hcp* and with a gold atom of the first layer below in the ring center, with either boron or nitrogen atoms of borazines interacting with surface gold atoms, i.e. structures differing by 60° rotation. The adsorption energy (ΔE) was calculated as the difference of the energy of the whole system compared of the energy of a neat Au(111) layer and the energy of a borazine molecule as calculated alone in a cell of the same shape. Borazine molecules remain almost flat upon adsorption (within dihedral angle of 2°), with only a slight distortion due to molecule-surface interactions. When modeling adsorption of hydrogen molecules, H_2 molecules were added next to the hydrogen atoms of borazine either horizontally or vertically with respect to the surface (for the horizontal position, both along the B-H/N-H bond and perpendicular to it).
For comparison, the dimer binding energy in the gas phase is calculated here to be 0.13 eV, the difference to the CCSD(T)/CBS value of 0.104 eV[10] can be accounted for the fact that the zero-point correction (0.03 eV) is not included in our calculations.

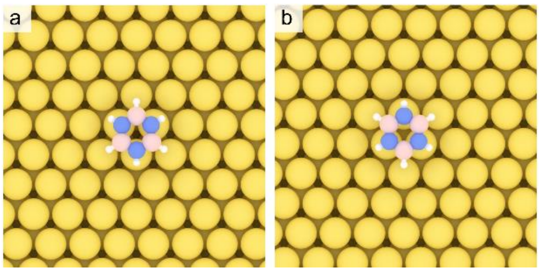

Figure S4. Optimized structures of the borazine monomer adsorbed on a Au(111) surface in *hcp* (a) and *fcc* (b) conformation, as obtained through PBE calculations.

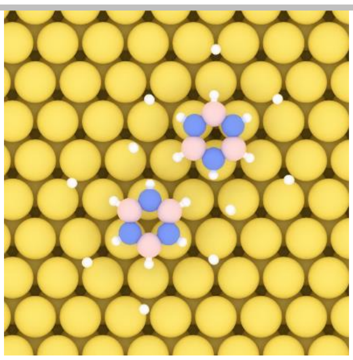

Figure S5. Optimized structures of dimer II in presence of eight H_2, as obtained through PBE calculations.

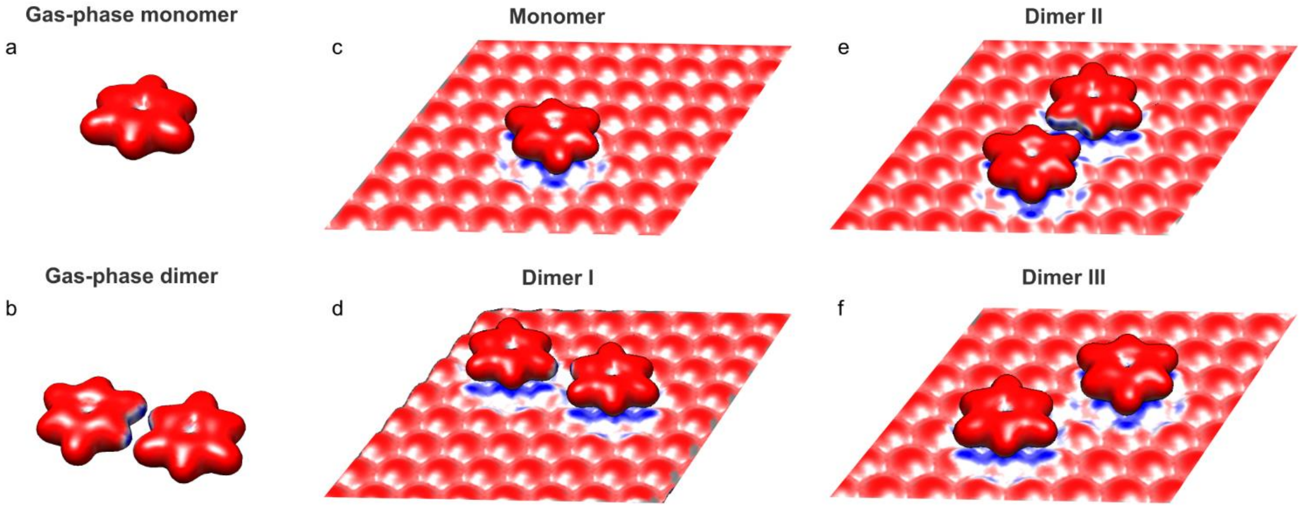

Figure S6. Calculated charge densities for the gas-phase monomer (a) and dimer (b), as well as the monomer (c), dimer I (d), dimer II (e), and dimer III (f) adsorbed on the Au(111) surface, obtained through PBE calculations. The isosurface is set at $\rho = 0.044$ au, with red regions indicating areas of lower negative charge, and blue regions indicating areas of higher negative charge.

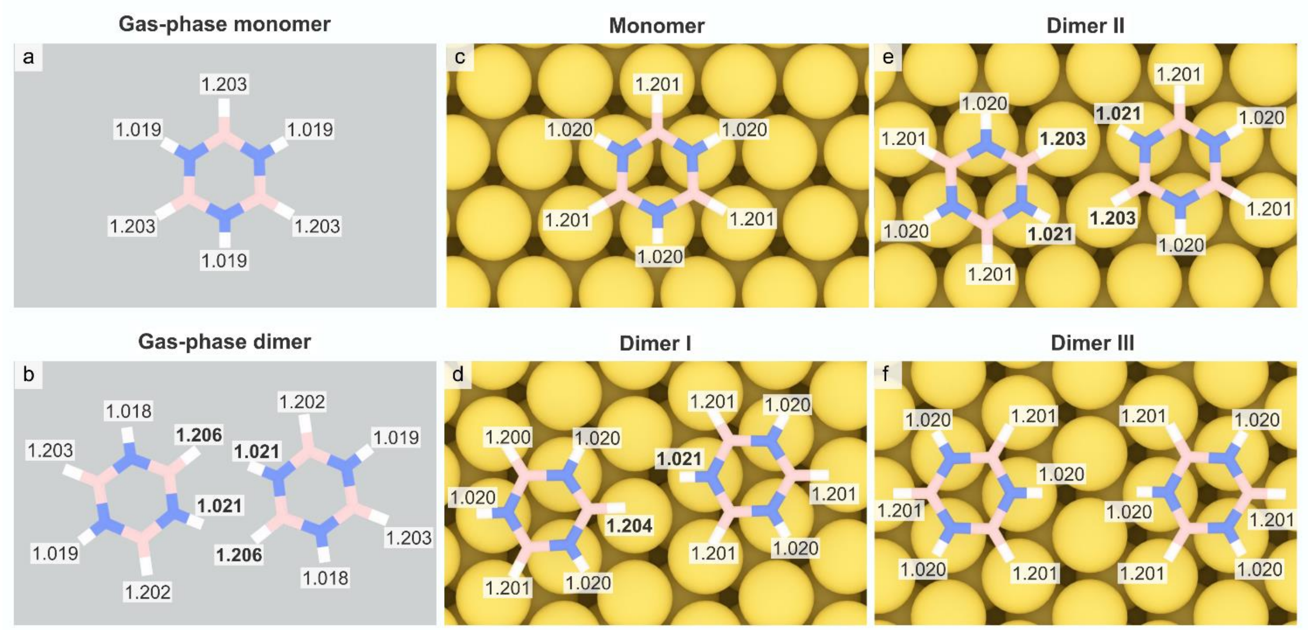

Figure S7. Optimized structures of the gas-phase monomer (a) and dimer (b), along with the monomer (c), dimer I (d), dimer II (e), and dimer III (f) adsorbed on the Au(111) surface, obtained from PBE calculations. The N-H and B-H bond lengths are shown in Ångstroms, with bond lengths involving H atoms participating in dihydrogen bonding (DHB) highlighted in bold for clarity.

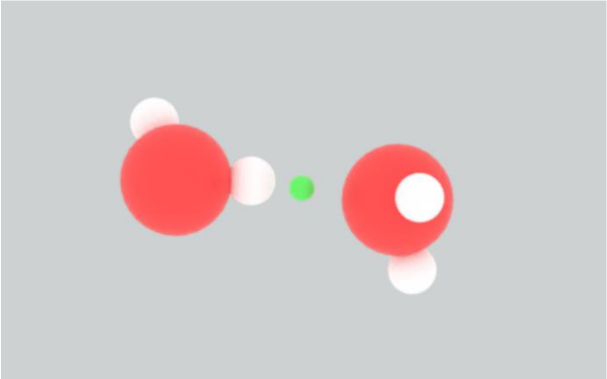

Figure S8. Calculated bond critical point (green) for the gas-phase water dimer. Red and white spheres represent oxygen and hydrogen, respectively.

References

[1] D. Nečas, P. Klapetek, *Cent Eur J Phys* **2012**, *10*, 181–188.
[2] M. G. Cuxart, K. Seufert, V. Chesnyak, W. A. Waqas, A. Robert, M.-L. Bocquet, G. S. Duesberg, H. Sachdev, W. Auwärter, *Sci. Adv.* **2021**, *7*, eabk1490.
[3] S. Li, A. Yu, F. Toledo, Z. Han, H. Wang, H. Y. He, R. Wu, W. Ho, *Phys. Rev. Lett.* **2013**, *111*, 146102.
[4] S. Li, D. Yuan, A. Yu, G. Czap, R. Wu, W. Ho, *Phys. Rev. Lett.* **2015**, *114*, 1–5.
[5] H. Wang, S. Li, H. He, A. Yu, F. Toledo, Z. Han, W. Ho, R. Wu, *J. Phys. Chem. Lett.* **2015**, *6*, 3453–3457.
[6] C. Weiss, C. Wagner, C. Kleimann, M. Rohlfing, F. S. Tautz, R. Temirov, *Phys. Rev. Lett.* **2010**, *105*, 2–5.
[7] R. Temirov, S. Soubatch, O. Neucheva, A. C. Lassise, F. S. Tautz, *New J. Phys.* **2008**, *10*, 053012.
[8] A. Yu, S. Li, G. Czap, W. Ho, *J. Phys. Chem. C* **2015**, *119*, 14737–14741.
[9] V. G. Ruiz, W. Liu, A. Tkatchenko, *Phys. Rev. B* **2016**, *93*, 035118.
[10] K. Verma, K. S. Viswanathan, *Phys. Chem. Chem. Phys.* **2017**, *19*, 19067–19074.

Coordinates (in Å and fractional coordinates, VASP format) of DFT optimized structures

```
Neat Au(111) surface
    23.0697829620000014    0.0000000000000000    0.0000000000000000
   -11.5348914810000007   19.9790169730000002    0.0000000000000000
     0.0000000000000000    0.0000000000000000   30.0000000000000000
   Au
   320
Selective dynamics
Direct
  0.0000000000000000  0.0000000000000000  0.0000000000000000   F   F   F
  0.0416667146899670  0.0833334293799339  0.0784850000000006   F   F   F
  0.0833332951369883  0.0416667146899670  0.1569700000000012   F   F   F
 -0.0000000000000000  0.0000000000000000  0.2386017402791817   T   T   T
  0.0416667146899670  0.0833334293799339  0.3224147603230061   T   T   T
  0.0000001342429456  0.1250001440699009  0.0000000000000000   F   F   F
  0.0416668489329126  0.2083335734498348  0.0784850000000006   F   F   F
  0.0833334293799339  0.1666668587598679  0.1569700000000012   F   F   F
  0.0000001342429456  0.1250001440699009  0.2386017402791817   T   T   T
  0.0416668489329126  0.2083335734498348  0.3224147603230061   T   T   T
  0.0000000182233322  0.2499997876146764  0.0000000000000000   F   F   F
  0.0416667329132991  0.3333332169946104  0.0784850000000006   F   F   F
  0.0833333133603205  0.2916665023046434  0.1569700000000012   F   F   F
  0.0000000182233322  0.2499997876146764  0.2386017402791817   T   T   T
  0.0416667329132991  0.3333332169946104  0.3224147603230061   T   T   T
  0.0000001524662778  0.3749999316845773  0.0000000000000000   F   F   F
  0.0416668671562448  0.4583333610645113  0.0784850000000006   F   F   F
  0.0833334476032661  0.4166666463745443  0.1569700000000012   F   F   F
  0.0000001524662778  0.3749999316845773  0.2386017402791817   T   T   T
  0.0416668671562448  0.4583333610645113  0.3224147603230061   T   T   T
  0.9999998532417820  0.5000000757544782  0.0000000000000000   F   F   F
  0.0416665679317489  0.5833335051344122  0.0784850000000006   F   F   F
  0.0833335818462118  0.5416667904444452  0.1569700000000012   F   F   F
  0.9999998532417820  0.5000000757544782  0.2386017402791817   T   T   T
  0.0416665679317489  0.5833335051344122  0.3224147603230061   T   T   T
  0.9999999874847276  0.6250002198243791  0.0000000000000000   F   F   F
  0.0416664519121284  0.7083331486791877  0.0784850000000006   F   F   F
  0.0833330323591497  0.6666664339892208  0.1569700000000012   F   F   F
  0.9999999874847276  0.6250002198243791  0.2386017402791817   T   T   T
  0.0416664519121284  0.7083331486791877  0.3224147603230061   T   T   T
  0.9999998714651142  0.7499998633691547  0.0000000000000000   F   F   F
  0.0416665861550811  0.8333332927490886  0.0784850000000006   F   F   F
  0.0833331666020953  0.7916665780591217  0.1569700000000012   F   F   F
  0.9999998714651142  0.7499998633691547  0.2386017402791817   T   T   T
  0.0416665861550811  0.8333332927490886  0.3224147603230061   T   T   T
  0.0000000057080598  0.8750000074390556  0.0000000000000000   F   F   F
  0.0416667203980268  0.9583334368189895  0.0784850000000006   F   F   F
  0.0833333008450481  0.9166667221290226  0.1569700000000012   F   F   F
  0.0000000057080598  0.8750000074390556  0.2386017402791817   T   T   T
  0.0416667203980268  0.9583334368189895  0.3224147603230061   T   T   T
  0.1249998755840025  0.0000000000000000  0.0000000000000000   F   F   F
  0.1666665902739695  0.0833334293799339  0.0784850000000006   F   F   F
  0.2083331707209908  0.0416667146899670  0.1569700000000012   F   F   F
  0.1249998755840025 -0.0000000000000000  0.2386017402791817   T   T   T
  0.1666665902739695  0.0833334293799339  0.3224147603230061   T   T   T
  0.1250000098269552  0.1250001440699009  0.0000000000000000   F   F   F
  0.1666667245169222  0.2083335734498348  0.0784850000000006   F   F   F
  0.2083333049639364  0.1666668587598679  0.1569700000000012   F   F   F
  0.1250000098269552  0.1250001440699009  0.2386017402791817   T   T   T
  0.1666667245169222  0.2083335734498348  0.3224147603230061   T   T   T
  0.1249998938073347  0.2499997876146764  0.0000000000000000   F   F   F
  0.1666666084973016  0.3333332169946104  0.0784850000000006   F   F   F
  0.2083331889443230  0.2916665023046434  0.1569700000000012   F   F   F
  0.1249998938073347  0.2499997876146764  0.2386017402791817   T   T   T
  0.1666666084973016  0.3333332169946104  0.3224147603230061   T   T   T
  0.1250000280502874  0.3749999316845773  0.0000000000000000   F   F   F
  0.1666667427402544  0.4583333610645113  0.0784850000000006   F   F   F
  0.2083333231872686  0.4166666463745443  0.1569700000000012   F   F   F
  0.1250000280502874  0.3749999316845773  0.2386017402791817   T   T   T
  0.1666667427402544  0.4583333610645113  0.3224147603230061   T   T   T
  0.1250001622932331  0.5000000757544782  0.0000000000000000   F   F   F
  0.1666668769832000  0.5833335051344122  0.0784850000000006   F   F   F
  0.2083334574302214  0.5416667904444452  0.1569700000000012   F   F   F
  0.1250001622932331  0.5000000757544782  0.2386017402791817   T   T   T
```

0.1666668769832000	0.5833335051344122	0.3224147603230061	T	T	T
0.1250002965361787	0.6250002198243791	0.0000000000000000	F	F	F
0.1666667609635866	0.7083331486791877	0.0784850000000006	F	F	F
0.2083333414106079	0.6666664339892208	0.1569700000000012	F	F	F
0.1250002965361787	0.6250002198243791	0.2386017402791817	T	T	T
0.1666667609635866	0.7083331486791877	0.3224147603230061	T	T	T
0.1249997470491166	0.7499998633691547	0.0000000000000000	F	F	F
0.1666664617390836	0.8333332927490886	0.0784850000000006	F	F	F
0.2083334756535535	0.7916665780591217	0.1569700000000012	F	F	F
0.1249997470491166	0.7499998633691547	0.2386017402791817	T	T	T
0.1666664617390836	0.8333332927490886	0.3224147603230061	T	T	T
0.1249998812920623	0.8750000074390556	0.0000000000000000	F	F	F
0.1666665959820293	0.9583334368189895	0.0784850000000006	F	F	F
0.2083331764290506	0.9166667221290226	0.1569700000000012	F	F	F
0.1249998812920623	0.8750000074390556	0.2386017402791817	T	T	T
0.1666665959820293	0.9583334368189895	0.3224147603230061	T	T	T
0.2500001846354607	0.0000000000000000	0.0000000000000000	F	F	F
0.2916668993254277	0.0833334293799339	0.0784850000000006	F	F	F
0.3333334797724490	0.0416667146899670	0.1569700000000012	F	F	F
0.2500001846354607	-0.0000000000000000	0.2386017402791817	T	T	T
0.2916668993254277	0.0833334293799339	0.3224147603230061	T	T	T
0.2499998854109577	0.1250001440699009	0.0000000000000000	F	F	F
0.2916666001009247	0.2083335734498348	0.0784850000000006	F	F	F
0.3333336140153946	0.1666668587598679	0.1569700000000012	F	F	F
0.2499998854109577	0.1250001440699009	0.2386017402791817	T	T	T
0.2916666001009247	0.2083335734498348	0.3224147603230061	T	T	T
0.2499997693913443	0.2499997876146764	0.0000000000000000	F	F	F
0.2916664840813112	0.3333332169946104	0.0784850000000006	F	F	F
0.3333330645283255	0.2916665023046434	0.1569700000000012	F	F	F
0.2499997693913443	0.2499997876146764	0.2386017402791817	T	T	T
0.2916664840813112	0.3333332169946104	0.3224147603230061	T	T	T
0.2499999036342899	0.3749999316845773	0.0000000000000000	F	F	F
0.2916666183242569	0.4583333610645113	0.0784850000000006	F	F	F
0.3333331987712782	0.4166666463745443	0.1569700000000012	F	F	F
0.2499999036342899	0.3749999316845773	0.2386017402791817	T	T	T
0.2916666183242569	0.4583333610645113	0.3224147603230061	T	T	T
0.2500000378772356	0.5000000757544782	0.0000000000000000	F	F	F
0.2916667525672025	0.5833335051344122	0.0784850000000006	F	F	F
0.3333333330142239	0.5416667904444452	0.1569700000000012	F	F	F
0.2500000378772356	0.5000000757544782	0.2386017402791817	T	T	T
0.2916667525672025	0.5833335051344122	0.3224147603230061	T	T	T
0.2500001721201883	0.6250002198243791	0.0000000000000000	F	F	F
0.2916666365475891	0.7083331486791877	0.0784850000000006	F	F	F
0.3333332169946104	0.6666664339892208	0.1569700000000012	F	F	F
0.2500001721201883	0.6250002198243791	0.2386017402791817	T	T	T
0.2916666365475891	0.7083331486791877	0.3224147603230061	T	T	T
0.2500000561005749	0.7499998633691547	0.0000000000000000	F	F	F
0.2916667707905418	0.8333332927490886	0.0784850000000006	F	F	F
0.3333333512375560	0.7916665780591217	0.1569700000000012	F	F	F
0.2500000561005749	0.7499998633691547	0.2386017402791817	T	T	T
0.2916667707905418	0.8333332927490886	0.3224147603230061	T	T	T
0.2500001903435205	0.8750000074390556	0.0000000000000000	F	F	F
0.2916669050334875	0.9583334368189895	0.0784850000000006	F	F	F
0.3333334854805088	0.9166667221290226	0.1569700000000012	F	F	F
0.2500001903435205	0.8750000074390556	0.2386017402791817	T	T	T
0.2916669050334875	0.9583334368189895	0.3224147603230061	T	T	T
0.3750000602194632	0.0000000000000000	0.0000000000000000	F	F	F
0.4166667749094302	0.0833334293799339	0.0784850000000006	F	F	F
0.4583333553564515	0.0416667146899670	0.1569700000000012	F	F	F
0.3750000602194632	0.0000000000000000	0.2386017402791817	T	T	T
0.4166667749094302	0.0833334293799339	0.3224147603230061	T	T	T
0.3750001944624159	0.1250001440699009	0.0000000000000000	F	F	F
0.4166669091523829	0.2083335734498348	0.0784850000000006	F	F	F
0.4583334895993971	0.1666668587598679	0.1569700000000012	F	F	F
0.3750001944624159	0.1250001440699009	0.2386017402791817	T	T	T
0.4166669091523829	0.2083335734498348	0.3224147603230061	T	T	T
0.3750000784427954	0.2499997876146764	0.0000000000000000	F	F	F
0.4166667931327623	0.3333332169946104	0.0784850000000006	F	F	F
0.4583333735797837	0.2916665023046434	0.1569700000000012	F	F	F
0.3750000784427954	0.2499997876146764	0.2386017402791817	T	T	T
0.4166667931327623	0.3333332169946104	0.3224147603230061	T	T	T
0.3749997792182995	0.3749999316845773	0.0000000000000000	F	F	F
0.4166664939082665	0.4583333610645113	0.0784850000000006	F	F	F
0.4583335078227293	0.4166666463745443	0.1569700000000012	F	F	F
0.3749997792182995	0.3749999316845773	0.2386017402791817	T	T	T

0.4166664939082665	0.4583333610645113	0.3224147603230061	T	T	T
0.3749999134612452	0.5000000757544782	0.0000000000000000	F	F	F
0.4166666281512121	0.5833335051344122	0.0784850000000006	F	F	F
0.4583332085982335	0.5416667904444452	0.1569700000000012	F	F	F
0.3749999134612452	0.5000000757544782	0.2386017402791817	T	T	T
0.4166666281512121	0.5833335051344122	0.3224147603230061	T	T	T
0.3750000477041908	0.6250002198243791	0.0000000000000000	F	F	F
0.4166665121315987	0.7083331486791877	0.0784850000000006	F	F	F
0.4583330925786129	0.6666664339892208	0.1569700000000012	F	F	F
0.3750000477041908	0.6250002198243791	0.2386017402791817	T	T	T
0.4166665121315987	0.7083331486791877	0.3224147603230061	T	T	T
0.3749999316845773	0.7499998633691547	0.0000000000000000	F	F	F
0.4166666463745443	0.8333332927490886	0.0784850000000006	F	F	F
0.4583332268215656	0.7916665780591217	0.1569700000000012	F	F	F
0.3749999316845773	0.7499998633691547	0.2386017402791817	T	T	T
0.4166666463745443	0.8333332927490886	0.3224147603230061	T	T	T
0.3750000659275230	0.8750000074390556	0.0000000000000000	F	F	F
0.4166667806174900	0.9583334368189895	0.0784850000000006	F	F	F
0.4583333610645113	0.9166667221290226	0.1569700000000012	F	F	F
0.3750000659275230	0.8750000074390556	0.2386017402791817	T	T	T
0.4166667806174900	0.9583334368189895	0.3224147603230061	T	T	T
0.4999999358034728	0.0000000000000000	0.0000000000000000	F	F	F
0.5416666504934398	0.0833334293799339	0.0784850000000006	F	F	F
0.5833332309404540	0.0416667146899670	0.1569700000000012	F	F	F
0.4999999358034728	-0.0000000000000000	0.2386017402791817	T	T	T
0.5416666504934398	0.0833334293799339	0.3224147603230061	T	T	T
0.5000000700464184	0.1250001440699009	0.0000000000000000	F	F	F
0.5416667847363854	0.2083335734498348	0.0784850000000006	F	F	F
0.5833333651834067	0.1666668587598679	0.1569700000000012	F	F	F
0.5000000700464184	0.1250001440699009	0.2386017402791817	T	T	T
0.5416667847363854	0.2083335734498348	0.3224147603230061	T	T	T
0.4999999540268050	0.2499997876146764	0.0000000000000000	F	F	F
0.5416666687167719	0.3333332169946104	0.0784850000000006	F	F	F
0.5833332491637861	0.2916665023046434	0.1569700000000012	F	F	F
0.4999999540268050	0.2499997876146764	0.2386017402791817	T	T	T
0.5416666687167719	0.3333332169946104	0.3224147603230061	T	T	T
0.5000000882697506	0.3749999316845773	0.0000000000000000	F	F	F
0.5416668029597176	0.4583333610645113	0.0784850000000006	F	F	F
0.5833333834067389	0.4166666463745443	0.1569700000000012	F	F	F
0.5000000882697506	0.3749999316845773	0.2386017402791817	T	T	T
0.5416668029597176	0.4583333610645113	0.3224147603230061	T	T	T
0.5000002225126963	0.5000000757544782	0.0000000000000000	F	F	F
0.5416669372026632	0.5833335051344122	0.0784850000000006	F	F	F
0.5833335176496846	0.5416667904444452	0.1569700000000012	F	F	F
0.5000002225126963	0.5000000757544782	0.2386017402791817	T	T	T
0.5416669372026632	0.5833335051344122	0.3224147603230061	T	T	T
0.4999999232882004	0.6250002198243791	0.0000000000000000	F	F	F
0.5416663877156012	0.7083331486791877	0.0784850000000006	F	F	F
0.5833334016300711	0.6666664339892208	0.1569700000000012	F	F	F
0.4999999232882004	0.6250002198243791	0.2386017402791817	T	T	T
0.5416663877156012	0.7083331486791877	0.3224147603230061	T	T	T
0.4999998072685798	0.7499998633691547	0.0000000000000000	F	F	F
0.5416665219585468	0.8333332927490886	0.0784850000000006	F	F	F
0.5833331024055681	0.7916665780591217	0.1569700000000012	F	F	F
0.4999998072685798	0.7499998633691547	0.2386017402791817	T	T	T
0.5416665219585468	0.8333332927490886	0.3224147603230061	T	T	T
0.4999999415115326	0.8750000074390556	0.0000000000000000	F	F	F
0.5416666562014996	0.9583334368189895	0.0784850000000006	F	F	F
0.5833332366485138	0.9166667221290226	0.1569700000000012	F	F	F
0.4999999415115326	0.8750000074390556	0.2386017402791817	T	T	T
0.5416666562014996	0.9583334368189895	0.3224147603230061	T	T	T
0.6249998113874753	0.0000000000000000	0.0000000000000000	F	F	F
0.6666665260774423	0.0833334293799339	0.0784850000000006	F	F	F
0.7083335399919122	0.0416667146899670	0.1569700000000012	F	F	F
0.6249998113874753	-0.0000000000000000	0.2386017402791817	T	T	T
0.6666665260774423	0.0833334293799339	0.3224147603230061	T	T	T
0.6249999456304209	0.1250001440699009	0.0000000000000000	F	F	F
0.6666666603203879	0.2083335734498348	0.0784850000000006	F	F	F
0.7083332407674092	0.1666668587598679	0.1569700000000012	F	F	F
0.6249999456304209	0.1250001440699009	0.2386017402791817	T	T	T
0.6666666603203879	0.2083335734498348	0.3224147603230061	T	T	T
0.6249998296108075	0.2499997876146764	0.0000000000000000	F	F	F
0.6666665443007744	0.3333332169946104	0.0784850000000006	F	F	F
0.7083331247477958	0.2916665023046434	0.1569700000000012	F	F	F
0.6249998296108075	0.2499997876146764	0.2386017402791817	T	T	T

0.6666665443007744	0.3333332169946104	0.3224147603230061	T	T	T
0.6249999638537531	0.3749999316845773	0.0000000000000000	F	F	F
0.6666666785437201	0.4583333610645113	0.0784850000000006	F	F	F
0.7083332589907414	0.4166666463745443	0.1569700000000012	F	F	F
0.6249999638537531	0.3749999316845773	0.2386017402791817	T	T	T
0.6666666785437201	0.4583333610645113	0.3224147603230061	T	T	T
0.6250000980967059	0.5000000757544782	0.0000000000000000	F	F	F
0.6666668127866728	0.5833335051344122	0.0784850000000006	F	F	F
0.7083333932336870	0.5416667904444452	0.1569700000000012	F	F	F
0.6250000980967059	0.5000000757544782	0.2386017402791817	T	T	T
0.6666668127866728	0.5833335051344122	0.3224147603230061	T	T	T
0.6250002323396515	0.6250002198243791	0.0000000000000000	F	F	F
0.6666666967670594	0.7083331486791877	0.0784850000000006	F	F	F
0.7083332772140736	0.6666664339892208	0.1569700000000012	F	F	F
0.6250002323396515	0.6250002198243791	0.2386017402791817	T	T	T
0.6666666967670594	0.7083331486791877	0.3224147603230061	T	T	T
0.6250001163200380	0.7499998633691547	0.0000000000000000	F	F	F
0.6666668310100050	0.8333332927490886	0.0784850000000006	F	F	F
0.7083334114570263	0.7916665780591217	0.1569700000000012	F	F	F
0.6250001163200380	0.7499998633691547	0.2386017402791817	T	T	T
0.6666668310100050	0.8333332927490886	0.3224147603230061	T	T	T
0.6249998170955351	0.8750000074390556	0.0000000000000000	F	F	F
0.6666665317855021	0.9583334368189895	0.0784850000000006	F	F	F
0.7083335456999720	0.9166667221290226	0.1569700000000012	F	F	F
0.6249998170955351	0.8750000074390556	0.2386017402791817	T	T	T
0.6666665317855021	0.9583334368189895	0.3224147603230061	T	T	T
0.7500001204389335	0.0000000000000000	0.0000000000000000	F	F	F
0.7916668351289005	0.0833334293799339	0.0784850000000006	F	F	F
0.8333334155759147	0.0416667146899670	0.1569700000000012	F	F	F
0.7500001204389335	0.0000000000000000	0.2386017402791817	T	T	T
0.7916668351289005	0.0833334293799339	0.3224147603230061	T	T	T
0.7500002546818791	0.1250001440699009	0.0000000000000000	F	F	F
0.7916669693718461	0.2083335734498348	0.0784850000000006	F	F	F
0.8333335498188674	0.1666668587598679	0.1569700000000012	F	F	F
0.7500002546818791	0.1250001440699009	0.2386017402791817	T	T	T
0.7916669693718461	0.2083335734498348	0.3224147603230061	T	T	T
0.7499997051948100	0.2499997876146764	0.0000000000000000	F	F	F
0.7916664198847769	0.3333332169946104	0.0784850000000006	F	F	F
0.8333334337992468	0.2916665023046434	0.1569700000000012	F	F	F
0.7499997051948100	0.2499997876146764	0.2386017402791817	T	T	T
0.7916664198847769	0.3333332169946104	0.3224147603230061	T	T	T
0.7499998394377627	0.3749999316845773	0.0000000000000000	F	F	F
0.7916665541277297	0.4583333610645113	0.0784850000000006	F	F	F
0.8333331345747439	0.4166666463745443	0.1569700000000012	F	F	F
0.7499998394377627	0.3749999316845773	0.2386017402791817	T	T	T
0.7916665541277297	0.4583333610645113	0.3224147603230061	T	T	T
0.7499999736807084	0.5000000757544782	0.0000000000000000	F	F	F
0.7916666883706753	0.5833335051344122	0.0784850000000006	F	F	F
0.8333332688176966	0.5416667904444452	0.1569700000000012	F	F	F
0.7499999736807084	0.5000000757544782	0.2386017402791817	T	T	T
0.7916666883706753	0.5833335051344122	0.3224147603230061	T	T	T
0.7500001079236540	0.6250002198243791	0.0000000000000000	F	F	F
0.7916665723510619	0.7083331486791877	0.0784850000000006	F	F	F
0.8333331527980832	0.6666664339892208	0.1569700000000012	F	F	F
0.7500001079236540	0.6250002198243791	0.2386017402791817	T	T	T
0.7916665723510619	0.7083331486791877	0.3224147603230061	T	T	T
0.7499999919040405	0.7499998633691547	0.0000000000000000	F	F	F
0.7916667065940075	0.8333332927490886	0.0784850000000006	F	F	F
0.8333332870410288	0.7916665780591217	0.1569700000000012	F	F	F
0.7499999919040405	0.7499998633691547	0.2386017402791817	T	T	T
0.7916667065940075	0.8333332927490886	0.3224147603230061	T	T	T
0.7500001261469933	0.8750000074390556	0.0000000000000000	F	F	F
0.7916668408369603	0.9583334368189895	0.0784850000000006	F	F	F
0.8333334212839745	0.9166667221290226	0.1569700000000012	F	F	F
0.7500001261469933	0.8750000074390556	0.2386017402791817	T	T	T
0.7916668408369603	0.9583334368189895	0.3224147603230061	T	T	T
0.8749999960229360	0.0000000000000000	0.0000000000000000	F	F	F
0.9166667107129030	0.0833334293799339	0.0784850000000006	F	F	F
0.9583332911599243	0.0416667146899670	0.1569700000000012	F	F	F
0.8749999960229360	0.0000000000000000	0.2386017402791817	T	T	T
0.9166667107129030	0.0833334293799339	0.3224147603230061	T	T	T
0.8750001302658816	0.1250001440699009	0.0000000000000000	F	F	F
0.9166668449558486	0.2083335734498348	0.0784850000000006	F	F	F
0.9583334254028699	0.1666668587598679	0.1569700000000012	F	F	F
0.8750001302658816	0.1250001440699009	0.2386017402791817	T	T	T

```
  0.9166668449558486  0.2083335734498348  0.3224147603230061   T   T   T
  0.8750000142462682  0.2499997876146764  0.0000000000000000   F   F   F
  0.9166667289362351  0.3333332169946104  0.0784850000000006   F   F   F
  0.9583333093832564  0.2916665023046434  0.1569700000000012   F   F   F
  0.8750000142462682  0.2499997876146764  0.2386017402791817   T   T   T
  0.9166667289362351  0.3333332169946104  0.3224147603230061   T   T   T
  0.8750001484892138  0.3749999316845773  0.0000000000000000   F   F   F
  0.9166668631791808  0.4583333610645113  0.0784850000000006   F   F   F
  0.9583334436262021  0.4166666463745443  0.1569700000000012   F   F   F
  0.8750001484892138  0.3749999316845773  0.2386017402791817   T   T   T
  0.9166668631791808  0.4583333610645113  0.3224147603230061   T   T   T
  0.8749998492647180  0.5000000757544782  0.0000000000000000   F   F   F
  0.9166665639546849  0.5833335051344122  0.0784850000000006   F   F   F
  0.9583335778691477  0.5416667904444452  0.1569700000000012   F   F   F
  0.8749998492647180  0.5000000757544782  0.2386017402791817   T   T   T
  0.9166665639546849  0.5833335051344122  0.3224147603230061   T   T   T
  0.8749999835076636  0.6250002198243791  0.0000000000000000   F   F   F
  0.9166664479350644  0.7083331486791877  0.0784850000000006   F   F   F
  0.9583330283820857  0.6666664339892208  0.1569700000000012   F   F   F
  0.8749999835076636  0.6250002198243791  0.2386017402791817   T   T   T
  0.9166664479350644  0.7083331486791877  0.3224147603230061   T   T   T
  0.8749998674880501  0.7499998633691547  0.0000000000000000   F   F   F
  0.9166665821780171  0.8333332927490886  0.0784850000000006   F   F   F
  0.9583331626250313  0.7916665780591217  0.1569700000000012   F   F   F
  0.8749998674880501  0.7499998633691547  0.2386017402791817   T   T   T
  0.9166665821780171  0.8333332927490886  0.3224147603230061   T   T   T
  0.8750000017309958  0.8750000074390556  0.0000000000000000   F   F   F
  0.9166667164209628  0.9583334368189895  0.0784850000000006   F   F   F
  0.9583332968679841  0.9166667221290226  0.1569700000000012   F   F   F
  0.8750000017309958  0.8750000074390556  0.2386017402791817   T   T   T
  0.9166667164209628  0.9583334368189895  0.3224147603230061   T   T   T
```

Borazine adsorbed on Au(111), fcc

```
    23.0697829620000014    0.0000000000000000    0.0000000000000000
   -11.5348914810000007   19.9790169730000002    0.0000000000000000
     0.0000000000000000    0.0000000000000000   30.0000000000000000
   Au   B    N    H
   320     3     3     6
Selective dynamics
Direct
  0.0000000000000000  0.0000000000000000  0.0000000000000000   F   F   F
  0.0416667146899670  0.0833334293799339  0.0784850000000006   F   F   F
  0.0833332951369883  0.0416667146899670  0.1569700000000012   F   F   F
  0.0000160592660004  0.0000824101868994  0.2385903343651345   T   T   T
  0.0416890688594192  0.0834359121303156  0.3224531366555959   T   T   T
  0.0000001342429456  0.1250001440699009  0.0000000000000000   F   F   F
  0.0416668489329126  0.2083335734498348  0.0784850000000006   F   F   F
  0.0833334293799339  0.1666668587598679  0.1569700000000012   F   F   F
  0.0000214474884563  0.1250725875698087  0.2385955806855374   T   T   T
  0.0417019989709336  0.2084313455404652  0.3224667396325719   T   T   T
  0.0000000182233322  0.2499997876146764  0.0000000000000000   F   F   F
  0.0416667329132991  0.3333332169946104  0.0784850000000006   F   F   F
  0.0833333133603205  0.2916665023046434  0.1569700000000012   F   F   F
  0.0000670478711396  0.2500812913532237  0.2385948821470338   T   T   T
  0.0417376009516099  0.3334464581765001  0.3223702263568261   T   T   T
  0.0000001524662778  0.3749999316845773  0.0000000000000000   F   F   F
  0.0416668671562448  0.4583333610645113  0.0784850000000006   F   F   F
  0.0833334476032661  0.4166666463745443  0.1569700000000012   F   F   F
  0.0000756922583793  0.3750812274772664  0.2385949940338609   T   T   T
  0.0417210373874813  0.4584503263573055  0.3224602539217810   T   T   T
  0.9999997248487205  0.5000000757544782  0.0000000000000000   F   F   F
  0.0416665679317489  0.5833335051344122  0.0784850000000006   F   F   F
  0.0833335818462118  0.5416667904444452  0.1569700000000012   F   F   F
  0.0000405775900372  0.5000518181521261  0.2385968409436032   T   T   T
  0.0417189455599258  0.5834382342356161  0.3224641974791492   T   T   T
  0.9999998590916661  0.6250002198243791  0.0000000000000000   F   F   F
  0.0416664519121284  0.7083331486791877  0.0784850000000006   F   F   F
  0.0833330323591497  0.6666664339892208  0.1569700000000012   F   F   F
  0.0000321281835850  0.6250326016413928  0.2385955140630000   T   T   T
  0.0417522210116292  0.7084507720982216  0.3223808930586209   T   T   T
  0.9999997430720526  0.7499998633691547  0.0000000000000000   F   F   F
  0.0416665861550811  0.8333332927490886  0.0784850000000006   F   F   F
  0.0833331666020953  0.7916665780591217  0.1569700000000012   F   F   F
  0.0000315831670588  0.7500363508826305  0.2385638802277331   T   T   T
  0.0417479253808527  0.8334588296609430  0.3223406152667365   T   T   T
```

0.0000000057080598	0.8750000074390556	0.0000000000000000	F	F	F
0.0416667203980268	0.9583334368189895	0.0784850000000006	F	F	F
0.0833333008450481	0.9166667221290226	0.1569700000000012	F	F	F
0.0000224060605926	0.8750733623661942	0.2385610529994890	T	T	T
0.0417252749714718	0.9584619671563017	0.3223750103233236	T	T	T
0.1249998755840025	0.0000000000000000	0.0000000000000000	F	F	F
0.1666665902739695	0.0833334293799339	0.0784850000000006	F	F	F
0.2083331707209908	0.0416667146899670	0.1569700000000012	F	F	F
0.1250254457972090	0.0000913385810211	0.2385652099847558	T	T	T
0.1667085419147076	0.0834667517801443	0.3223782345101731	T	T	T
0.1250000098269552	0.1250001440699009	0.0000000000000000	F	F	F
0.1666667245169222	0.2083335734498348	0.0784850000000006	F	F	F
0.2083333049639364	0.1666668587598679	0.1569700000000012	F	F	F
0.1249863516850996	0.1250792665822608	0.2386063318682305	T	T	T
0.1666607678831198	0.2084196106345914	0.3225469426173825	T	T	T
0.1249998938073347	0.2499997876146764	0.0000000000000000	F	F	F
0.1666666084973016	0.3333332169946104	0.0784850000000006	F	F	F
0.2083331889443230	0.2916665023046434	0.1569700000000012	F	F	F
0.1250195141349813	0.2500665478818964	0.2386466328045715	T	T	T
0.1667166587328592	0.3334013807074748	0.3225920198056800	T	T	T
0.1250000280502874	0.3749999316845773	0.0000000000000000	F	F	F
0.1666667427402544	0.4583333610645113	0.0784850000000006	F	F	F
0.2083333231872686	0.4166666463745443	0.1569700000000012	F	F	F
0.1251004037214080	0.3750862444042685	0.2386634885152906	T	T	T
0.1667162845318813	0.4584565942549095	0.3225764301066594	T	T	T
0.1250001622932331	0.5000000757544782	0.0000000000000000	F	F	F
0.1666668769832000	0.5833335051344122	0.0784850000000006	F	F	F
0.2083334574302214	0.5416667904444452	0.1569700000000012	F	F	F
0.1250377096995188	0.5000483897081603	0.2386500565167810	T	T	T
0.1666622786099144	0.5834091161193248	0.3225665458467867	T	T	T
0.1250002965361787	0.6250002198243791	0.0000000000000000	F	F	F
0.1666667609635866	0.7083331486791877	0.0784850000000006	F	F	F
0.2083333414106079	0.6666664339892208	0.1569700000000012	F	F	F
0.1249943595774436	0.6249981253952990	0.2386144169597353	T	T	T
0.1667351874562576	0.7084305534913176	0.3223901020765795	T	T	T
0.1249997470491166	0.7499998633691547	0.0000000000000000	F	F	F
0.1666664617390836	0.8333332927490886	0.0784850000000006	F	F	F
0.2083334756535535	0.7916665780591217	0.1569700000000012	F	F	F
0.1250380591481481	0.7500353245148468	0.2385645376315103	T	T	T
0.1667424965866857	0.8334571280175492	0.3223399068847111	T	T	T
0.1249998812920623	0.8750000074390556	0.0000000000000000	F	F	F
0.1666665959820293	0.9583334368189895	0.0784850000000006	F	F	F
0.2083331764290506	0.9166667221290226	0.1569700000000012	F	F	F
0.1250438912977013	0.8750655773678333	0.2385611201610541	T	T	T
0.1667274768319637	0.9584597603259936	0.3223422800582594	T	T	T
0.2500001846354607	0.0000000000000000	0.0000000000000000	F	F	F
0.2916668993254277	0.0833334293799339	0.0784850000000006	F	F	F
0.3333334797724490	0.0416667146899670	0.1569700000000012	F	F	F
0.2500620480726536	0.0000874602041673	0.2385642293819977	T	T	T
0.2917297833241572	0.0834618671719262	0.3223763147981907	T	T	T
0.2499998854109577	0.1250001440699009	0.0000000000000000	F	F	F
0.2916666001009247	0.2083335734498348	0.0784850000000006	F	F	F
0.3333336140153946	0.1666668587598679	0.1569700000000012	F	F	F
0.2500319960381965	0.1250793569507712	0.2386310331216709	T	T	T
0.2917377585343088	0.2084919758361979	0.3225136677617327	T	T	T
0.2499997693913443	0.2499997876146764	0.0000000000000000	F	F	F
0.2916664840813112	0.3333332169946104	0.0784850000000006	F	F	F
0.3333330645283255	0.2916665023046434	0.1569700000000012	F	F	F
0.2499067496958737	0.2499399070059814	0.2386673540893449	T	T	T
0.2916187108808060	0.3333175437839832	0.3226580762668418	T	T	T
0.2499999036342899	0.3749999316845773	0.0000000000000000	F	F	F
0.2916666183242569	0.4583333610645113	0.0784850000000006	F	F	F
0.3333331987712782	0.4166666463745443	0.1569700000000012	F	F	F
0.2499341453860379	0.3749426527557844	0.2387037518139811	T	T	T
0.2915183013907663	0.4583350779063118	0.3225247678351634	T	T	T
0.2500000378772356	0.5000000757544782	0.0000000000000000	F	F	F
0.2916667525672025	0.5833335051344122	0.0784850000000006	F	F	F
0.3333333330142239	0.5416667904444452	0.1569700000000012	F	F	F
0.2499475985689924	0.5000555060911974	0.2387016684525545	T	T	T
0.2915650041232637	0.5834668725744685	0.3226049008406521	T	T	T
0.2500001721201883	0.6250002198243791	0.0000000000000000	F	F	F
0.2916666365475891	0.7083331486791877	0.0784850000000006	F	F	F
0.3333332169946104	0.6666664339892208	0.1569700000000012	F	F	F
0.2498872818709465	0.6250583631379508	0.2386531146874909	T	T	T
0.2917445659148376	0.7084128461008835	0.3225442643261173	T	T	T

0.2500000561005749	0.7499998633691547	0.0000000000000000	F	F	F
0.2916667707905418	0.8333332927490886	0.0784850000000006	F	F	F
0.3333333512375560	0.7916665780591217	0.1569700000000012	F	F	F
0.2500347851673493	0.7500466975609583	0.2386485081859389	T	T	T
0.2917487242583772	0.8334503667770273	0.3223751632449269	T	T	T
0.2500001903435205	0.8750000074390556	0.0000000000000000	F	F	F
0.2916669050334875	0.9583334368189895	0.0784850000000006	F	F	F
0.3333334854805088	0.9166667221290226	0.1569700000000012	F	F	F
0.2500688461308869	0.8750691033403635	0.2385609647189393	T	T	T
0.2917311065786246	0.9584547461219675	0.3223721254192826	T	T	T
0.3750000602194632	0.0000000000000000	0.0000000000000000	F	F	F
0.4166667749094302	0.0833334293799339	0.0784850000000006	F	F	F
0.4583333553564515	0.0416667146899670	0.1569700000000012	F	F	F
0.3750630616500609	0.0000754879629904	0.2385901634210641	T	T	T
0.4167194022332963	0.0834251262522604	0.3224505898907526	T	T	T
0.3750001944624159	0.1250001440699009	0.0000000000000000	F	F	F
0.4166669091523829	0.2083335734498348	0.0784850000000006	F	F	F
0.4583334895993971	0.1666668587598679	0.1569700000000012	F	F	F
0.3750779013262076	0.1250735250566355	0.2386055747211897	T	T	T
0.4167406333357926	0.2084114045583904	0.3225462197598627	T	T	T
0.3750000784427954	0.2499997876146764	0.0000000000000000	F	F	F
0.4166667931327623	0.3333332169946104	0.0784850000000006	F	F	F
0.4583333735797837	0.2916665023046434	0.1569700000000012	F	F	F
0.3750220010297329	0.2499357312661011	0.2386675098546855	T	T	T
0.4167132541224061	0.3333128082768092	0.3226591724572858	T	T	T
0.3749997792182995	0.3749999316845773	0.0000000000000000	F	F	F
0.4166664939082665	0.4583333610645113	0.0784850000000006	F	F	F
0.4583335078227293	0.4166666463745443	0.1569700000000012	F	F	F
0.3747863653829338	0.3745616254369630	0.2385137008598879	T	T	T
0.4165359390220971	0.4580239857303312	0.3211786582240395	T	T	T
0.3749999134612452	0.5000000757544782	0.0000000000000000	F	F	F
0.4166666281512121	0.5833335051344122	0.0784850000000006	F	F	F
0.4583332085982335	0.5416667904444452	0.1569700000000012	F	F	F
0.3747990843797835	0.4998900259443104	0.2384534096729046	T	T	T
0.4164837952876278	0.5836993003832214	0.3208049841221690	T	T	T
0.3750000477041908	0.6250002198243791	0.0000000000000000	F	F	F
0.4166665121315987	0.7083331486791877	0.0784850000000006	F	F	F
0.4583330925786129	0.6666664339892208	0.1569700000000012	F	F	F
0.3747540906609672	0.6253109255958136	0.2384294590852624	T	T	T
0.4166976443609684	0.7086137015474986	0.3225634477734292	T	T	T
0.3749999316845773	0.7499998633691547	0.0000000000000000	F	F	F
0.4166666463745443	0.8333332927490886	0.0784850000000006	F	F	F
0.4583332268215656	0.7916665780591217	0.1569700000000012	F	F	F
0.3749996654354371	0.7502022946088098	0.2386454208617742	T	T	T
0.4167542855293080	0.8335050092638362	0.3225605594886281	T	T	T
0.3750000659275230	0.8750000074390556	0.0000000000000000	F	F	F
0.4166667806174900	0.9583334368189895	0.0784850000000006	F	F	F
0.4583333610645113	0.9166667221290226	0.1569700000000012	F	F	F
0.3750941606142256	0.8751049646302264	0.2386149833238288	T	T	T
0.4167308616277680	0.9584679920973705	0.3224612707944484	T	T	T
0.4999999358034728	0.0000000000000000	0.0000000000000000	F	F	F
0.5416666504934398	0.0833334293799339	0.0784850000000006	F	F	F
0.5833332309404540	0.0416667146899670	0.1569700000000012	F	F	F
0.5000441691691865	0.0000573037468683	0.2385962022455310	T	T	T
0.5417155917378390	0.0834381304127844	0.3224235222345618	T	T	T
0.5000000700464184	0.1250001440699009	0.0000000000000000	F	F	F
0.5416667847363854	0.2083335734498348	0.0784850000000006	F	F	F
0.5833333651834067	0.1666668587598679	0.1569700000000012	F	F	F
0.5000343868945877	0.1250631213115494	0.2385948578995603	T	T	T
0.5417051063603769	0.2084156383450357	0.3224670746356065	T	T	T
0.4999999540268050	0.2499997876146764	0.0000000000000000	F	F	F
0.5416666687167719	0.3333332169946104	0.0784850000000006	F	F	F
0.5833332491637861	0.2916665023046434	0.1569700000000012	F	F	F
0.5000333784517802	0.2500563308479012	0.2386477332121116	T	T	T
0.5416909647302771	0.3333872970230334	0.3225969128836906	T	T	T
0.5000000882697506	0.3749999316845773	0.0000000000000000	F	F	F
0.5416668029597176	0.4583333610645113	0.0784850000000006	F	F	F
0.5833333834067389	0.4166666463745443	0.1569700000000012	F	F	F
0.5000125358281635	0.3749326743128431	0.2387010419756858	T	T	T
0.5418661637343384	0.4583288106446257	0.3225020959770920	T	T	T
0.5000002225126963	0.5000000757544782	0.0000000000000000	F	F	F
0.5416669372026632	0.5833335051344122	0.0784850000000006	F	F	F
0.5833335176496846	0.5416667904444452	0.1569700000000012	F	F	F
0.5001170267997977	0.4998858730907291	0.2384461120007707	T	T	T
0.5422867089236449	0.5837120472353331	0.3207581985837014	T	T	T

0.4999999232882004	0.6250002198243791	0.0000000000000000	F	F	F
0.5416663877156012	0.7083331486791877	0.0784850000000006	F	F	F
0.5833334016300711	0.6666664339892208	0.1569700000000012	F	F	F
0.5001580691923957	0.6252888177469471	0.2383681492924001	T	T	T
0.5419209755248912	0.7087836678435302	0.3222434477173307	T	T	T
0.4999998072685798	0.7499998633691547	0.0000000000000000	F	F	F
0.5416665219585468	0.8333332927490886	0.0784850000000006	F	F	F
0.5833331024055681	0.7916665780591217	0.1569700000000012	F	F	F
0.5000105613724913	0.7501847256349238	0.2386490721425346	T	T	T
0.5417126057949613	0.8334873076300638	0.3225834966470547	T	T	T
0.4999999415115326	0.8750000074390556	0.0000000000000000	F	F	F
0.5416666562014996	0.9583334368189895	0.0784850000000006	F	F	F
0.5833332366485138	0.9166667221290226	0.1569700000000012	F	F	F
0.5000434113815517	0.8750705461546231	0.2386477880388142	T	T	T
0.5417130263513537	0.9584556614109982	0.3224755492647391	T	T	T
0.6249998113874753	0.0000000000000000	0.0000000000000000	F	F	F
0.6666665260774423	0.0833334293799339	0.0784850000000006	F	F	F
0.7083335399919122	0.0416667146899670	0.1569700000000012	F	F	F
0.6250064913004242	0.0000062034375297	0.2386021675786227	T	T	T
0.6667025366167020	0.0834366603621137	0.3223849525144470	T	T	T
0.6249999456304209	0.1250001440699009	0.0000000000000000	F	F	F
0.6666666603203879	0.2083335734498348	0.0784850000000006	F	F	F
0.7083332407674092	0.1666668587598679	0.1569700000000012	F	F	F
0.6250147844019939	0.1250459661249696	0.2385904910227548	T	T	T
0.6666999180563246	0.2084211619179716	0.3223872757555841	T	T	T
0.6249998296108075	0.2499997876146764	0.0000000000000000	F	F	F
0.6666665443007744	0.3333332169946104	0.0784850000000006	F	F	F
0.7083331247477958	0.2916665023046434	0.1569700000000012	F	F	F
0.6249992863128464	0.2500717531401611	0.2385959470097325	T	T	T
0.6667107428181145	0.3334321243366241	0.3223725291748882	T	T	T
0.6249999638537531	0.3749999316845773	0.0000000000000000	F	F	F
0.6666666785437201	0.4583333610645113	0.0784850000000006	F	F	F
0.7083332589907414	0.4166666463745443	0.1569700000000012	F	F	F
0.6249875464037888	0.3750772159719538	0.2386648766422488	T	T	T
0.6667794378227108	0.4584462848983953	0.3225762524214014	T	T	T
0.6250000980967059	0.5000000757544782	0.0000000000000000	F	F	F
0.6666668127866728	0.5833335051344122	0.0784850000000006	F	F	F
0.7083333932336870	0.5416667904444452	0.1569700000000012	F	F	F
0.6251376435219896	0.5000555585225729	0.2386965204458197	T	T	T
0.6669680120163465	0.5834679385580042	0.3225919999478752	T	T	T
0.6250002323396515	0.6250002198243791	0.0000000000000000	F	F	F
0.6666666967670594	0.7083331486791877	0.0784850000000006	F	F	F
0.7083332772140736	0.6666664339892208	0.1569700000000012	F	F	F
0.6256046562998371	0.6253182663495098	0.2384207137109645	T	T	T
0.6669849364905939	0.7086230309707084	0.3225637442462476	T	T	T
0.6250001163200380	0.7499998633691547	0.0000000000000000	F	F	F
0.6666668310100050	0.8333332927490886	0.0784850000000006	F	F	F
0.7083334114570263	0.7916665780591217	0.1569700000000012	F	F	F
0.6252134414662420	0.7501862640339975	0.2386500165807033	T	T	T
0.6668129444635192	0.8334882475657216	0.3225839022326539	T	T	T
0.6249998170955351	0.8750000074390556	0.0000000000000000	F	F	F
0.6666665317855021	0.9583334368189895	0.0784850000000006	F	F	F
0.7083335456999720	0.9166667221290226	0.1569700000000012	F	F	F
0.6250064603746927	0.8749866591791383	0.2386650109030440	T	T	T
0.6667180545508284	0.9584271954213448	0.3223794533347088	T	T	T
0.7500001204389335	0.0000000000000000	0.0000000000000000	F	F	F
0.7916668351289005	0.0833334293799339	0.0784850000000006	F	F	F
0.8333334155759147	0.0416667146899670	0.1569700000000012	F	F	F
0.7500058750223448	0.0000090670324008	0.2386024256118014	T	T	T
0.7917216298438309	0.0834417089433270	0.3223854671398894	T	T	T
0.7500002546818791	0.1250001440699009	0.0000000000000000	F	F	F
0.7916669693718461	0.2083335734498348	0.0784850000000006	F	F	F
0.8333335498188674	0.1666668587598679	0.1569700000000012	F	F	F
0.7500158258236025	0.1250441423733764	0.2385880788018098	T	T	T
0.7917121375733681	0.2084369468662992	0.3223946292368918	T	T	T
0.7499997051948100	0.2499997876146764	0.0000000000000000	F	F	F
0.7916664198847769	0.3333332169946104	0.0784850000000006	F	F	F
0.8333334337992468	0.2916665023046434	0.1569700000000012	F	F	F
0.7500189533380697	0.2500575206822914	0.2385873895513150	T	T	T
0.7917322259249771	0.3334464122517118	0.3223783504487422	T	T	T
0.7499998394377627	0.3749999316845773	0.0000000000000000	F	F	F
0.7916665541277297	0.4583333610645113	0.0784850000000006	F	F	F
0.8333331345747439	0.4166666463745443	0.1569700000000012	F	F	F
0.7500027304263676	0.3750738829105779	0.2385956804077309	T	T	T
0.7917605515685840	0.4584400203047374	0.3224604801911757	T	T	T

0.7499999736807084	0.5000000757544782	0.0000000000000000	F	F	F
0.7916666883706753	0.5833335051344122	0.0784850000000006	F	F	F
0.8333332688176966	0.5416667904444452	0.1569700000000012	F	F	F
0.7500297972572518	0.5000419441816010	0.2386495993850557	T	T	T
0.7917994517654738	0.5834045175524718	0.3225651122473866	T	T	T
0.7500001079236540	0.6250002198243791	0.0000000000000000	F	F	F
0.7916665723510619	0.7083331486791877	0.0784850000000006	F	F	F
0.8333331527980832	0.6666664339892208	0.1569700000000012	F	F	F
0.7502143683957465	0.6250626105712238	0.2386512105390924	T	T	T
0.7917249641257352	0.7084136925745372	0.3225489904058180	T	T	T
0.7499999919040405	0.7499998633691547	0.0000000000000000	F	F	F
0.7916667065940075	0.8333332927490886	0.0784850000000006	F	F	F
0.8333332870410288	0.7916665780591217	0.1569700000000012	F	F	F
0.7502459296196698	0.7502084721126954	0.2386455051899685	T	T	T
0.7917915231920772	0.8335109834090078	0.3225649257230027	T	T	T
0.7500001261469933	0.8750000074390556	0.0000000000000000	F	F	F
0.7916668408369603	0.9583334368189895	0.0784850000000006	F	F	F
0.8333334212839745	0.9166667221290226	0.1569700000000012	F	F	F
0.7500532420148986	0.8750746608648723	0.2386483599568571	T	T	T
0.7917519915230613	0.9584619015491096	0.3224748654456921	T	T	T
0.8749999960229360	0.0000000000000000	0.0000000000000000	F	F	F
0.9166667107129030	0.0833334293799339	0.0784850000000006	F	F	F
0.9583332911599243	0.0416667146899670	0.1569700000000012	F	F	F
0.8750197523913962	0.0000659575355161	0.2385949893266836	T	T	T
0.9167116307088706	0.0834515762369632	0.3224245122723278	T	T	T
0.8750001302658816	0.1250001440699009	0.0000000000000000	F	F	F
0.9166668449558486	0.2083335734498348	0.0784850000000006	F	F	F
0.9583334254028699	0.1666668587598679	0.1569700000000012	F	F	F
0.8750249058722882	0.1250541088771660	0.2385905547428234	T	T	T
0.9167135268835643	0.2084351439003534	0.3223878580571163	T	T	T
0.8750000142462682	0.2499997876146764	0.0000000000000000	F	F	F
0.9166667289362351	0.3333332169946104	0.0784850000000006	F	F	F
0.9583333093832564	0.2916665023046434	0.1569700000000012	F	F	F
0.8750300067868249	0.2500629666928998	0.2385872493126994	T	T	T
0.9167256503728450	0.3334541066728871	0.3223780901760834	T	T	T
0.8750001484892138	0.3749999316845773	0.0000000000000000	F	F	F
0.9166668631791808	0.4583333610645113	0.0784850000000006	F	F	F
0.9583334436262021	0.4166666463745443	0.1569700000000012	F	F	F
0.8750352524048489	0.3750687855549502	0.2385885024206458	T	T	T
0.9167388334578909	0.4584419643648290	0.3224233838713951	T	T	T
0.8749998492647180	0.5000000757544782	0.0000000000000000	F	F	F
0.9166665639546849	0.5833335051344122	0.0784850000000006	F	F	F
0.9583335778691477	0.5416667904444452	0.1569700000000012	F	F	F
0.8750283100469880	0.5000490571017728	0.2385949962456681	T	T	T
0.9167708219544763	0.5834343643495539	0.3224633196973464	T	T	T
0.8749999835076636	0.6250002198243791	0.0000000000000000	F	F	F
0.9166664479350644	0.7083331486791877	0.0784850000000006	F	F	F
0.9583330283820857	0.6666664339892208	0.1569700000000012	F	F	F
0.8750390715792831	0.6249970908259023	0.2386144114820282	T	T	T
0.9167507455302246	0.7084283412100667	0.3223847279345599	T	T	T
0.8749998674880501	0.7499998633691547	0.0000000000000000	F	F	F
0.9166665821780171	0.8333332927490886	0.0784850000000006	F	F	F
0.9583331626250313	0.7916665780591217	0.1569700000000012	F	F	F
0.8750475547198707	0.7500510981514640	0.2386489455462041	T	T	T
0.9167381354041818	0.8334531816386493	0.3223726990932861	T	T	T
0.8750000017309958	0.8750000074390556	0.0000000000000000	F	F	F
0.9166667164209628	0.9583334368189895	0.0784850000000006	F	F	F
0.9583332968679841	0.9166667221290226	0.1569700000000012	F	F	F
0.8750357837611958	0.8751101760139146	0.2386153265545460	T	T	T
0.9167439085418626	0.9584762141790304	0.3224613228991354	T	T	T
0.4985231317624903	0.5111316541796056	0.4249580864249467	T	T	T
0.4980725323787391	0.6205597256357003	0.4240104380912588	T	T	T
0.3886593248302897	0.5106751784241700	0.4250994431084564	T	T	T
0.5324360415664221	0.5830292854121019	0.4243391102527191	T	T	T
0.4266493037913939	0.4768038295975132	0.4255281683221306	T	T	T
0.4262021215962384	0.5825864727810881	0.4244902106737157	T	T	T
0.5288002721647466	0.4812991964917966	0.4251325313109072	T	T	T
0.4012886911705003	0.4257494645840658	0.4256950051748943	T	T	T
0.3285616978319402	0.4804680057094995	0.4253690661946297	T	T	T
0.4005179583423151	0.6079608839532756	0.4238438536830658	T	T	T
0.5279444898522178	0.6806768102135600	0.4235244686184481	T	T	T
0.5834934761620081	0.6087239433834507	0.4235830982453749	T	T	T

Borazine adsorbed on Au(111), hpc

23.0697829620000014	0.0000000000000000	0.0000000000000000

```
  -11.5348914810000007   19.9790169730000002    0.0000000000000000
    0.0000000000000000    0.0000000000000000   30.0000000000000000
  Au   B    N    H
  320    3    3    6
Selective dynamics
Direct
  0.0000000000000000  0.0000000000000000  0.0000000000000000   F   F   F
  0.0416667146899670  0.0833334293799339  0.0784850000000006   F   F   F
  0.0833332951369883  0.0416667146899670  0.1569700000000012   F   F   F
  0.0000431922751564  0.0000488501489646  0.2385889258787894   T   T   T
  0.0416994886856237  0.0833846717342028  0.3224109774406589   T   T   T
  0.0000001342429456  0.1250001440699009  0.0000000000000000   F   F   F
  0.0416668489329126  0.2083335734498348  0.0784850000000006   F   F   F
  0.0833334293799339  0.1666668587598679  0.1569700000000012   F   F   F
  0.0000748330865545  0.1250657926988245  0.2385816417791171   T   T   T
  0.0416979042769607  0.2084150155376130  0.3224000013429126   T   T   T
  0.0000000182233322  0.2499997876146764  0.0000000000000000   F   F   F
  0.0416667329132991  0.3333332169946104  0.0784850000000006   F   F   F
  0.0833333133603205  0.2916665023046434  0.1569700000000012   F   F   F
  0.0000416220571696  0.2500534495710319  0.2385850505461532   T   T   T
  0.0416970651711471  0.3334231174689254  0.3224587094286327   T   T   T
  0.0000001524662778  0.3749999316845773  0.0000000000000000   F   F   F
  0.0416668671562448  0.4583333610645113  0.0784850000000006   F   F   F
  0.0833334476032661  0.4166666463745443  0.1569700000000012   F   F   F
  0.0000162219827141  0.3750239577851716  0.2386002703718936   T   T   T
  0.0417212092289550  0.4584224961160807  0.3224212106111984   T   T   T
  0.9999997248487205  0.5000000757544782  0.0000000000000000   F   F   F
  0.0416665679317489  0.5833335051344122  0.0784850000000006   F   F   F
  0.0833335818462118  0.5416667904444452  0.1569700000000012   F   F   F
  0.0000075982423941  0.5000023201163305  0.2385825334536233   T   T   T
  0.0417103043445149  0.5834019626535690  0.3223676924601968   T   T   T
  0.9999998590916661  0.6250002198243791  0.0000000000000000   F   F   F
  0.0416664519121284  0.7083331486791877  0.0784850000000006   F   F   F
  0.0833330323591497  0.6666664339892208  0.1569700000000012   F   F   F
  0.0000055704891762  0.6250310381774022  0.2385802067194648   T   T   T
  0.0417161726169642  0.7084174006638587  0.3223682987100818   T   T   T
  0.9999997430720526  0.7499998633691547  0.0000000000000000   F   F   F
  0.0416665861550811  0.8333332927490886  0.0784850000000006   F   F   F
  0.0833331666020953  0.7916665780591217  0.1569700000000012   F   F   F
  0.0000184604111414  0.7500684765157528  0.2385812719905820   T   T   T
  0.0417357107506200  0.8334115215596268  0.3224206927582515   T   T   T
  0.0000000057080598  0.8750000074390556  0.0000000000000000   F   F   F
  0.0416667203980268  0.9583334368189895  0.0784850000000006   F   F   F
  0.0833333008450481  0.9166667221290226  0.1569700000000012   F   F   F
  0.0000245485176897  0.8750544638390946  0.2386009430498119   T   T   T
  0.0417049874061355  0.9583857197262186  0.3224560916523718   T   T   T
  0.1249998755840025  0.0000000000000000  0.0000000000000000   F   F   F
  0.1666665902739695  0.0833334293799339  0.0784850000000006   F   F   F
  0.2083331707209908  0.0416667146899670  0.1569700000000012   F   F   F
  0.1250073409330755  0.0000567468266876  0.2386176337874436   T   T   T
  0.1666590268323807  0.0833340587178628  0.3225599846902216   T   T   T
  0.1250000098269552  0.1250001440699009  0.0000000000000000   F   F   F
  0.1666667245169222  0.2083335734498348  0.0784850000000006   F   F   F
  0.2083333049639364  0.1666668587598679  0.1569700000000012   F   F   F
  0.1250607033470136  0.1250594018683979  0.2386557948401141   T   T   T
  0.1666672506476239  0.2083829849359325  0.3225380617768382   T   T   T
  0.1249998938073347  0.2499997876146764  0.0000000000000000   F   F   F
  0.1666666084973016  0.3333332169946104  0.0784850000000006   F   F   F
  0.2083331889443230  0.2916665023046434  0.1569700000000012   F   F   F
  0.1250649997100298  0.2500592673913216  0.2386539351381108   T   T   T
  0.1666498611542742  0.3334277942226134  0.3225567677382124   T   T   T
  0.1250000280502874  0.3749999316845773  0.0000000000000000   F   F   F
  0.1666667427402544  0.4583333610645113  0.0784850000000006   F   F   F
  0.2083333231872686  0.4166666463745443  0.1569700000000012   F   F   F
  0.1250031405516100  0.3750083584451595  0.2386172085944941   T   T   T
  0.1667067547052360  0.4584041400412969  0.3224670302262484   T   T   T
  0.1250001622932331  0.5000000757544782  0.0000000000000000   F   F   F
  0.1666668769832000  0.5833335051344122  0.0784850000000006   F   F   F
  0.2083334574302214  0.5416667904444452  0.1569700000000012   F   F   F
  0.1250012977972416  0.5000028511279840  0.2385815577284869   T   T   T
  0.1667111595781666  0.5834023949350146  0.3223691116008435   T   T   T
  0.1250002965361787  0.6250002198243791  0.0000000000000000   F   F   F
  0.1666667609635866  0.7083331486791877  0.0784850000000006   F   F   F
  0.2083333414106079  0.6666664339892208  0.1569700000000012   F   F   F
  0.1250164284477538  0.6250227965433730  0.2385734875208912   T   T   T
```

0.1667198445962390	0.7084156648814744	0.3223495205257267	T	T	T
0.1249997470491166	0.7499998633691547	0.0000000000000000	F	F	F
0.1666664617390836	0.8333332927490886	0.0784850000000006	F	F	F
0.2083334756535535	0.7916665780591217	0.1569700000000012	F	F	F
0.1250212606022741	0.7500581268347950	0.2385758774610647	T	T	T
0.1667198779067986	0.8334278388292865	0.3223706707974988	T	T	T
0.1249998812920623	0.8750000074390556	0.0000000000000000	F	F	F
0.1666665959820293	0.9583334368189895	0.0784850000000006	F	F	F
0.2083331764290506	0.9166667221290226	0.1569700000000012	F	F	F
0.1250141630341869	0.8750669900920603	0.2385827791044766	T	T	T
0.1667212193132796	0.9584256199133049	0.3224598403943924	T	T	T
0.2500001846354607	0.0000000000000000	0.0000000000000000	F	F	F
0.2916668993254277	0.0833334293799339	0.0784850000000006	F	F	F
0.3333334797724490	0.0416667146899670	0.1569700000000012	F	F	F
0.2499835363830695	0.0000507943561997	0.2386435434117689	T	T	T
0.2917271985191473	0.0834905183991432	0.3225607269333585	T	T	T
0.2499998854109577	0.1250001440699009	0.0000000000000000	F	F	F
0.2916666001009247	0.2083335734498348	0.0784850000000006	F	F	F
0.3333336140153946	0.1666668587598679	0.1569700000000012	F	F	F
0.2499219120466561	0.1249679126704150	0.2386611543870320	T	T	T
0.2914796926259589	0.2082652094152430	0.3226390214749397	T	T	T
0.2499997693913443	0.2499997876146764	0.0000000000000000	F	F	F
0.2916664840813112	0.3333332169946104	0.0784850000000006	F	F	F
0.3333330645283255	0.2916665023046434	0.1569700000000012	F	F	F
0.2499841231861859	0.2500153843809870	0.2386723597343582	T	T	T
0.2914616958986291	0.3333261077549077	0.3226176411682144	T	T	T
0.2499999036342899	0.3749999316845773	0.0000000000000000	F	F	F
0.2916666183242569	0.4583333610645113	0.0784850000000006	F	F	F
0.3333331987712782	0.4166666463745443	0.1569700000000012	F	F	F
0.2499166765091319	0.3750142806003528	0.2386522446643545	T	T	T
0.2917054822715700	0.4583509766002090	0.3225653710111527	T	T	T
0.2500000378772356	0.5000000757544782	0.0000000000000000	F	F	F
0.2916667525672025	0.5833335051344122	0.0784850000000006	F	F	F
0.3333333330142239	0.5416667904444452	0.1569700000000012	F	F	F
0.2499656562573671	0.4999950817417154	0.2386463849657386	T	T	T
0.2917348023414235	0.5833828833850240	0.3223701840863876	T	T	T
0.2500001721201883	0.6250002198243791	0.0000000000000000	F	F	F
0.2916666365475891	0.7083331486791877	0.0784850000000006	F	F	F
0.3333332169946104	0.6666664339892208	0.1569700000000012	F	F	F
0.2500338370548463	0.6250263306619017	0.2385804189386988	T	T	T
0.2917214537106873	0.7084158694792860	0.3223673293819338	T	T	T
0.2500000561005749	0.7499998633691547	0.0000000000000000	F	F	F
0.2916667707905418	0.8333332927490886	0.0784850000000006	F	F	F
0.3333333512375560	0.7916665780591217	0.1569700000000012	F	F	F
0.2500477858864210	0.7500546817913447	0.2385728052151030	T	T	T
0.2917281659287926	0.8334293595254556	0.3223577292759690	T	T	T
0.2500001903435205	0.8750000074390556	0.0000000000000000	F	F	F
0.2916669050334875	0.9583334368189895	0.0784850000000006	F	F	F
0.3333334854805088	0.9166667221290226	0.1569700000000012	F	F	F
0.2500394334009555	0.8750767805340293	0.2385750378847285	T	T	T
0.2917465884405477	0.9584769394015821	0.3223580451441161	T	T	T
0.3750000602194632	0.0000000000000000	0.0000000000000000	F	F	F
0.4166667749094302	0.0833334293799339	0.0784850000000006	F	F	F
0.4583333553564515	0.0416667146899670	0.1569700000000012	F	F	F
0.3750655904632578	0.0000471041166120	0.2386384040943304	T	T	T
0.4167786526375494	0.0834937604821266	0.3225496365301242	T	T	T
0.3750001944624159	0.1250001440699009	0.0000000000000000	F	F	F
0.4166669091523829	0.2083335734498348	0.0784850000000006	F	F	F
0.4583334895993971	0.1666668587598679	0.1569700000000012	F	F	F
0.3749118140784740	0.1248257158966613	0.2386778683339228	T	T	T
0.4166760118672473	0.2083273318110385	0.3224941599944781	T	T	T
0.3750000784427954	0.2499997876146764	0.0000000000000000	F	F	F
0.4166667931327623	0.3333332169946104	0.0784850000000006	F	F	F
0.4583333735797837	0.2916665023046434	0.1569700000000012	F	F	F
0.3747771626736816	0.2496697398814658	0.2385171953151829	T	T	T
0.4162133900803770	0.3331806798279605	0.3210176412476629	T	T	T
0.3749997792182995	0.3749999316845773	0.0000000000000000	F	F	F
0.4166664939082665	0.4583333610645113	0.0784850000000006	F	F	F
0.4583335078227293	0.4166666463745443	0.1569700000000012	F	F	F
0.3747737130882079	0.3751522052831085	0.2384957823945500	T	T	T
0.4166413466319109	0.4585187559315705	0.3223457767952304	T	T	T
0.3749999134612452	0.5000000757544782	0.0000000000000000	F	F	F
0.4166666281512121	0.5833335051344122	0.0784850000000006	F	F	F
0.4583332085982335	0.5416667904444452	0.1569700000000012	F	F	F
0.3748813521822366	0.5001686706018749	0.2386487561975986	T	T	T

0.4167731356373290	0.5834288110422339	0.3225429785738348	T	T	T
0.3750000477041908	0.6250002198243791	0.0000000000000000	F	F	F
0.4166665121315987	0.7083331486791877	0.0784850000000006	F	F	F
0.4583330925786129	0.6666664339892208	0.1569700000000012	F	F	F
0.3750554181955329	0.6250979941619889	0.2386475225609768	T	T	T
0.4167150841019379	0.7084238209638575	0.3224668119947934	T	T	T
0.3749999316845773	0.7499998633691547	0.0000000000000000	F	F	F
0.4166666463745443	0.8333332927490886	0.0784850000000006	F	F	F
0.4583332268215656	0.7916665780591217	0.1569700000000012	F	F	F
0.3750617240613495	0.7500654783870117	0.2385833212900069	T	T	T
0.4166962966921063	0.8334117130599068	0.3224198434773448	T	T	T
0.3750000659275230	0.8750000074390556	0.0000000000000000	F	F	F
0.4166667806174900	0.9583334368189895	0.0784850000000006	F	F	F
0.4583333610645113	0.9166667221290226	0.1569700000000012	F	F	F
0.3750626129271483	0.8750645140697232	0.2385774681573733	T	T	T
0.4167222169545299	0.9584260748675310	0.3224531231169689	T	T	T
0.4999999358034728	0.0000000000000000	0.0000000000000000	F	F	F
0.5416666504934398	0.0833334293799339	0.0784850000000006	F	F	F
0.5833332309404540	0.0416667146899670	0.1569700000000012	F	F	F
0.5000548215453933	0.0000564363697229	0.2386154025166596	T	T	T
0.5416978722794368	0.0833325492797155	0.3225649387571113	T	T	T
0.5000000700464184	0.1250001440699009	0.0000000000000000	F	F	F
0.5416667847363854	0.2083335734498348	0.0784850000000006	F	F	F
0.5833333651834067	0.1666668587598679	0.1569700000000012	F	F	F
0.5000497032242821	0.1249608384359570	0.2386580390745248	T	T	T
0.5418090806546200	0.2082568427158436	0.3226373312374159	T	T	T
0.4999999540268050	0.2499997876146764	0.0000000000000000	F	F	F
0.5416666687167719	0.3333332169946104	0.0784850000000006	F	F	F
0.5833332491637861	0.2916665023046434	0.1569700000000012	F	F	F
0.4998956471906179	0.2496558980813096	0.2385105994995380	T	T	T
0.5419958102908581	0.3331706180262211	0.3209794895440694	T	T	T
0.5000000882697506	0.3749999316845773	0.0000000000000000	F	F	F
0.5416668029597176	0.4583333610645113	0.0784850000000006	F	F	F
0.5833333834067389	0.4166666463745443	0.1569700000000012	F	F	F
0.5000126216538100	0.3750185756178682	0.2384149800764902	T	T	T
0.5420291530239248	0.4590167831615598	0.3207401616168774	T	T	T
0.5000002225126963	0.5000000757544782	0.0000000000000000	F	F	F
0.5416669372026632	0.5833335051344122	0.0784850000000006	F	F	F
0.5833335176496846	0.5416667904444452	0.1569700000000012	F	F	F
0.4998933703695531	0.5003284270240798	0.2384500520028452	T	T	T
0.5418352868026564	0.5837422876018240	0.3225730040665205	T	T	T
0.4999999232882004	0.6250002198243791	0.0000000000000000	F	F	F
0.5416663877156012	0.7083331486791877	0.0784850000000006	F	F	F
0.5833334016300711	0.6666664339892208	0.1569700000000012	F	F	F
0.5000422471588180	0.6251771938556332	0.2386485239787164	T	T	T
0.5416927278675615	0.7084989813973986	0.3225780344841722	T	T	T
0.4999998072685798	0.7499998633691547	0.0000000000000000	F	F	F
0.5416665219585468	0.8333332927490886	0.0784850000000006	F	F	F
0.5833331024055681	0.7916665780591217	0.1569700000000012	F	F	F
0.5000567863010532	0.7500704671816846	0.2386265993299221	T	T	T
0.5416961314686218	0.8334404113711529	0.3224664116594329	T	T	T
0.4999999415115326	0.8750000074390556	0.0000000000000000	F	F	F
0.5416666562014996	0.9583334368189895	0.0784850000000006	F	F	F
0.5833332366485138	0.9166667221290226	0.1569700000000012	F	F	F
0.5000387271072305	0.8750512640378777	0.2386012201768689	T	T	T
0.5417014178653047	0.9583859394091622	0.3224534579534868	T	T	T
0.6249998113874753	0.0000000000000000	0.0000000000000000	F	F	F
0.6666665260774423	0.0833334293799339	0.0784850000000006	F	F	F
0.7083335399919122	0.0416667146899670	0.1569700000000012	F	F	F
0.6250121084495712	0.0000486058320117	0.2385856876372890	T	T	T
0.6667091313970448	0.0833862633889639	0.3224116667846010	T	T	T
0.6249999456304209	0.1250001440699009	0.0000000000000000	F	F	F
0.6666666603203879	0.2083335734498348	0.0784850000000006	F	F	F
0.7083332407674092	0.1666668587598679	0.1569700000000012	F	F	F
0.6250074109362467	0.1250609929862671	0.2386584845002565	T	T	T
0.6667381654481310	0.2083792797384931	0.3225456252559366	T	T	T
0.6249998296108075	0.2499997876146764	0.0000000000000000	F	F	F
0.6666665443007744	0.3333332169946104	0.0784850000000006	F	F	F
0.7083331247477958	0.2916665023046434	0.1569700000000012	F	F	F
0.6250414930256170	0.2500124401920964	0.2386748143914357	T	T	T
0.6668931576551761	0.3333170922153600	0.3226147473914427	T	T	T
0.6249999638537531	0.3749999316845773	0.0000000000000000	F	F	F
0.6666666785437201	0.4583333610645113	0.0784850000000006	F	F	F
0.7083332589907414	0.4166666463745443	0.1569700000000012	F	F	F
0.6253948674891887	0.3751475795663758	0.2384870947631675	T	T	T

0.6669177955156662	0.4585176763082494	0.3223218302745967	T	T	T
0.6250000980967059	0.5000000757544782	0.0000000000000000	F	F	F
0.6666668127866728	0.5833335051344122	0.0784850000000006	F	F	F
0.7083333932336870	0.5416667904444452	0.1569700000000012	F	F	F
0.6254461036805042	0.5003269123882399	0.2384463269976074	T	T	T
0.6669392060082396	0.5837436068126916	0.3225727137869382	T	T	T
0.6250002323396515	0.6250002198243791	0.0000000000000000	F	F	F
0.6666666967670594	0.7083331486791877	0.0784850000000006	F	F	F
0.7083332772140736	0.6666664339892208	0.1569700000000012	F	F	F
0.6250645375694737	0.6251131592989387	0.2386523426577982	T	T	T
0.6667551743502035	0.7084878462936798	0.3225543404396724	T	T	T
0.6250001163200380	0.7499998633691547	0.0000000000000000	F	F	F
0.6666668310100050	0.8333332927490886	0.0784850000000006	F	F	F
0.7083334114570263	0.7916665780591217	0.1569700000000012	F	F	F
0.6250062821341069	0.7500090463144864	0.2386618271878822	T	T	T
0.6667081045199537	0.8334431776851798	0.3224013404418321	T	T	T
0.6249998170955351	0.8750000074390556	0.0000000000000000	F	F	F
0.6666665317855021	0.9583334368189895	0.0784850000000006	F	F	F
0.7083335456999720	0.9166667221290226	0.1569700000000012	F	F	F
0.6250076881419636	0.8750244664432950	0.2385856837772644	T	T	T
0.6667031486168549	0.9584135121003947	0.3223838232620693	T	T	T
0.7500001204389335	0.0000000000000000	0.0000000000000000	F	F	F
0.7916668351289005	0.0833334293799339	0.0784850000000006	F	F	F
0.8333334155759147	0.0416667146899670	0.1569700000000012	F	F	F
0.7500104890530436	0.0000372991528224	0.2385765932020248	T	T	T
0.7917182894556117	0.0834146910583732	0.3223604719478028	T	T	T
0.7500002546818791	0.1250001440699009	0.0000000000000000	F	F	F
0.7916669693718461	0.2083335734498348	0.0784850000000006	F	F	F
0.8333335498188674	0.1666668587598679	0.1569700000000012	F	F	F
0.7499993075590727	0.1250647952084359	0.2385835652622705	T	T	T
0.7917416160585291	0.2084145661532830	0.3224003148739057	T	T	T
0.7499997051948100	0.2499997876146764	0.0000000000000000	F	F	F
0.7916664198847769	0.3333332169946104	0.0784850000000006	F	F	F
0.8333334337992468	0.2916665023046434	0.1569700000000012	F	F	F
0.7500020334582160	0.2500551034789967	0.2386579059838254	T	T	T
0.7918016492877835	0.3334214480153974	0.3225603838202414	T	T	T
0.7499998394377627	0.3749999316845773	0.0000000000000000	F	F	F
0.7916665541277297	0.4583333610645113	0.0784850000000006	F	F	F
0.8333331345747439	0.4166666463745443	0.1569700000000012	F	F	F
0.7501103582477172	0.3750113816284933	0.2386538097005707	T	T	T
0.7916751140020449	0.4583457643333030	0.3225621356681493	T	T	T
0.7499999736807084	0.5000000757544782	0.0000000000000000	F	F	F
0.7916666883706753	0.5833335051344122	0.0784850000000006	F	F	F
0.8333332688176966	0.5416667904444452	0.1569700000000012	F	F	F
0.7503098333914666	0.5001728027948098	0.2386447668905023	T	T	T
0.7916830691535763	0.5834281644381160	0.3225512549036087	T	T	T
0.7500001079236540	0.6250002198243791	0.0000000000000000	F	F	F
0.7916665723510619	0.7083331486791877	0.0784850000000006	F	F	F
0.8333331527980832	0.6666664339892208	0.1569700000000012	F	F	F
0.7501505022131713	0.6251757467346082	0.2386453594802413	T	T	T
0.7918274751837078	0.7085003315334447	0.3225657042104980	T	T	T
0.7499999919040405	0.7499998633691547	0.0000000000000000	F	F	F
0.7916667065940075	0.8333332927490886	0.0784850000000006	F	F	F
0.8333332870410288	0.7916665780591217	0.1569700000000012	F	F	F
0.7500103937773035	0.7500087912333996	0.2386584874752268	T	T	T
0.7917498625658729	0.8334434586509063	0.3223953975627892	T	T	T
0.7500001261469933	0.8750000074390556	0.0000000000000000	F	F	F
0.7916668408369603	0.9583334368189895	0.0784850000000006	F	F	F
0.8333334212839745	0.9166667221290226	0.1569700000000012	F	F	F
0.7499973095978275	0.8749923981448675	0.2385799108484575	T	T	T
0.7917195452509219	0.9584208823658433	0.3223518655690892	T	T	T
0.8749999960229360	0.0000000000000000	0.0000000000000000	F	F	F
0.9166667107129030	0.0833334293799339	0.0784850000000006	F	F	F
0.9583332911599243	0.0416667146899670	0.1569700000000012	F	F	F
0.8750354866708661	0.0000357206535356	0.2385785257923624	T	T	T
0.9167190912683238	0.0834128951503190	0.3223607416380690	T	T	T
0.8750001302658816	0.1250001440699009	0.0000000000000000	F	F	F
0.9166668449558486	0.2083335734498348	0.0784850000000006	F	F	F
0.9583334254028699	0.1666668587598679	0.1569700000000012	F	F	F
0.8750353933790356	0.1250582888056386	0.2385806116766890	T	T	T
0.9167253029860166	0.2084232279873291	0.3223903601020432	T	T	T
0.8750000142462682	0.2499997876146764	0.0000000000000000	F	F	F
0.9166667289362351	0.3333332169946104	0.0784850000000006	F	F	F
0.9583333093832564	0.2916665023046434	0.1569700000000012	F	F	F
0.8750219270710047	0.2500537050906462	0.2385878750988218	T	T	T

0.9167485619789226 0.3334193590341606 0.3224608077167541 T T T
0.8750001484892138 0.3749999316845773 0.0000000000000000 F F F
0.9166668631791808 0.4583333610645113 0.0784850000000006 F F F
0.9583334436262021 0.4166666463745443 0.1569700000000012 F F F
0.8750145970571515 0.3750062412467143 0.2386210838480654 T T T
0.9167189288692561 0.4584003049792973 0.3224764656197019 T T T
0.8749998492647180 0.5000000757544782 0.0000000000000000 F F F
0.9166665639546849 0.5833335051344122 0.0784850000000006 F F F
0.9583335778691477 0.5416667904444452 0.1569700000000012 F F F
0.8750454426979284 0.4999957328114930 0.2386479473914358 T T T
0.9166716870227114 0.5833833075345108 0.3223718874881867 T T T
0.8749999835076636 0.6250002198243791 0.0000000000000000 F F F
0.9166664479350644 0.7083331486791877 0.0784850000000006 F F F
0.9583330283820857 0.6666664339892208 0.1569700000000012 F F F
0.8750579592647515 0.6251008108651238 0.2386475367561453 T T T
0.9167310051529433 0.7084250937825805 0.3224641199402443 T T T
0.8749998674880501 0.7499998633691547 0.0000000000000000 F F F
0.9166665821780171 0.8333332927490886 0.0784850000000006 F F F
0.9583331626250313 0.7916665780591217 0.1569700000000012 F F F
0.8750300491038828 0.7500769632137582 0.2386216580918650 T T T
0.9167624718057445 0.8334417115570288 0.3224663509609216 T T T
0.8750000017309958 0.8750000074390556 0.0000000000000000 F F F
0.9166667164209628 0.9583334368189895 0.0784850000000006 F F F
0.9583332968679841 0.9166667221290226 0.1569700000000012 F F F
0.8750274527175103 0.8750300757441655 0.2385855974546473 T T T
0.9167278688074065 0.9584111427115058 0.3223946449319174 T T T
0.5753796638917655 0.4151425064756368 0.4242899063343946 T T T
0.4658655149007977 0.4154431669915506 0.4243564219407636 T T T
0.4655687469489420 0.3056431138369070 0.4250034725380502 T T T
0.5377598670140127 0.4494258656768019 0.4241470527368331 T T T
0.5374697109504183 0.3432701509435259 0.4248318066987057 T T T
0.4316008321722401 0.3435633707596037 0.4249221373388460 T T T
0.6354953815764925 0.4450897675497759 0.4241434286055407 T T T
0.5629003654601088 0.3176365638763913 0.4246064640978772 T T T
0.4353968503009434 0.2455468669567303 0.4253639183902407 T T T
0.3805393664323758 0.3181470816204967 0.4247642333271714 T T T
0.4359532640089918 0.4456456740628821 0.4242367572894408 T T T
0.5633974066482309 0.5004816521534471 0.4233725718282645 T T T

Borazine dimer adsorbed on Au(111), dimer I
23.0697829620000014 0.0000000000000000 0.0000000000000000
-11.5348914810000007 19.9790169730000002 0.0000000000000000
0.0000000000000000 0.0000000000000000 30.0000000000000000
Au B N H
320 6 6 12
Selective dynamics
Direct
0.0000000000000000 0.0000000000000000 0.0000000000000000 F F F
0.0416667146899670 0.0833334293799339 0.0784850000000006 F F F
0.0833332951369883 0.0416667146899670 0.1569700000000012 F F F
0.9999634263423139 -0.0000122133656657 0.2386171706224730 T T T
0.0415513703153966 0.0831715004241697 0.3224476161333843 T T T
0.0000001342429456 0.1250001440699009 0.0000000000000000 F F F
0.0416668489329126 0.2083335734498348 0.0784850000000006 F F F
0.0833334293799339 0.1666668587598679 0.1569700000000012 F F F
-0.0000079505279927 0.1249619856191353 0.2385898618898550 T T T
0.0415661315594105 0.2082222434575048 0.3224182863792385 T T T
0.0000000182233322 0.2499997876146764 0.0000000000000000 F F F
0.0416667329132991 0.3333332169946104 0.0784850000000006 F F F
0.0833333133603205 0.2916665023046434 0.1569700000000012 F F F
-0.0000231169765436 0.2499435946088535 0.2385650813097671 T T T
0.0415516318805026 0.3332462532979221 0.3224264334460157 T T T
0.0000001524662778 0.3749999316845773 0.0000000000000000 F F F
0.0416668671562448 0.4583333610645113 0.0784850000000006 F F F
0.0833334476032661 0.4166666463745443 0.1569700000000012 F F F
-0.0000354301934321 0.3749583860088558 0.2385827247109530 T T T
0.0416034061666337 0.4583038451938021 0.3223717303944743 T T T
0.9999997248487205 0.5000000757544782 0.0000000000000000 F F F
0.0416665679317489 0.5833335051344122 0.0784850000000006 F F F
0.0833335818462118 0.5416667904444452 0.1569700000000012 F F F
0.9999711206234684 0.4999742802493763 0.2385610477467986 T T T
0.0416310226680852 0.5833099399307213 0.3223553270052462 T T T
0.9999998590916661 0.6250002198243791 0.0000000000000000 F F F
0.0416664519121284 0.7083331486791877 0.0784850000000006 F F F
0.0833330323591497 0.6666664339892208 0.1569700000000012 F F F

0.9999622963432535	0.6249853436852113	0.2385630969864414	T	T	T
0.0417011893785167	0.7083303685983533	0.3224125231675604	T	T	T
0.9999997430720526	0.7499998633691547	0.0000000000000000	F	F	F
0.0416665861550811	0.8333332927490886	0.0784850000000006	F	F	F
0.0833331666020953	0.7916665780591217	0.1569700000000012	F	F	F
0.9999663589929429	0.7499454663824258	0.2385994772483172	T	T	T
0.0416748511993528	0.8332855208180495	0.3224407897639216	T	T	T
0.0000000057080598	0.8750000074390556	0.0000000000000000	F	F	F
0.0416667203980268	0.9583334368189895	0.0784850000000006	F	F	F
0.0833333008450481	0.9166667221290226	0.1569700000000012	F	F	F
0.9999744929389695	0.8749262160918599	0.2386344853130896	T	T	T
0.0415439992375322	0.9581796876084407	0.3224006756568411	T	T	T
0.1249998755840025	0.0000000000000000	0.0000000000000000	F	F	F
0.1666665902739695	0.0833334293799339	0.0784850000000006	F	F	F
0.2083331707209908	0.0416667146899670	0.1569700000000012	F	F	F
0.1249255427498045	-0.0000390419301642	0.2385992182752396	T	T	T
0.1665351583715427	0.0831510608023622	0.3225396829962296	T	T	T
0.1250000098269552	0.1250001440699009	0.0000000000000000	F	F	F
0.1666667245169222	0.2083335734498348	0.0784850000000006	F	F	F
0.2083333049639364	0.1666668587598679	0.1569700000000012	F	F	F
0.1249699883296576	0.1249638358836705	0.2386396279766422	T	T	T
0.1664958211722642	0.2081638751153918	0.3225495190381326	T	T	T
0.1249998938073347	0.2499997876146764	0.0000000000000000	F	F	F
0.1666666084973016	0.3333332169946104	0.0784850000000006	F	F	F
0.2083331889443230	0.2916665023046434	0.1569700000000012	F	F	F
0.1249782183568886	0.2499542038436312	0.2386516007677137	T	T	T
0.1664555471260750	0.3332158459953336	0.3226024718339743	T	T	T
0.1250000280502874	0.3749999316845773	0.0000000000000000	F	F	F
0.1666667427402544	0.4583333610645113	0.0784850000000006	F	F	F
0.2083333231872686	0.4166666463745443	0.1569700000000012	F	F	F
0.1249398612383777	0.3749298870085053	0.2386043481214850	T	T	T
0.1665332116785349	0.4582333459659587	0.3224424434746835	T	T	T
0.1250001622932331	0.5000000757544782	0.0000000000000000	F	F	F
0.1666668769832000	0.5833335051344122	0.0784850000000006	F	F	F
0.2083334574302214	0.5416667904444452	0.1569700000000012	F	F	F
0.1249858285273305	0.4999685726901013	0.2385580322079655	T	T	T
0.1665980694596580	0.5832950575608477	0.3223598869451025	T	T	T
0.1250002965361787	0.6250002198243791	0.0000000000000000	F	F	F
0.1666667609635866	0.7083331486791877	0.0784850000000006	F	F	F
0.2083333414106079	0.6666664339892208	0.1569700000000012	F	F	F
0.1249823655521063	0.6249864722656207	0.2385573425045146	T	T	T
0.1666555970551839	0.7083264827390999	0.3223752774739835	T	T	T
0.1249997470491166	0.7499998633691547	0.0000000000000000	F	F	F
0.1666664617390836	0.8333332927490886	0.0784850000000006	F	F	F
0.2083334756535535	0.7916665780591217	0.1569700000000012	F	F	F
0.1249835743021611	0.7499839632815112	0.2385633176311925	T	T	T
0.1667157096334090	0.8333570388292068	0.3223519198531133	T	T	T
0.1249998812920623	0.8750000074390556	0.0000000000000000	F	F	F
0.1666665959820293	0.9583334368189895	0.0784850000000006	F	F	F
0.2083331764290506	0.9166667221290226	0.1569700000000012	F	F	F
0.1249664726373983	0.8749624233391324	0.2385591139615433	T	T	T
0.1666672147511054	0.9582905724973135	0.3224124869717306	T	T	T
0.2500001846354607	0.0000000000000000	0.0000000000000000	F	F	F
0.2916668993254277	0.0833334293799339	0.0784850000000006	F	F	F
0.3333334797724490	0.0416667146899670	0.1569700000000012	F	F	F
0.2499514053899252	0.9999541779942824	0.2386238060576498	T	T	T
0.2916781281840444	0.0833540030931566	0.3225187948825120	T	T	T
0.2499998854109577	0.1250001440699009	0.0000000000000000	F	F	F
0.2916666001009247	0.2083335734498348	0.0784850000000006	F	F	F
0.3333336140153946	0.1666668587598679	0.1569700000000012	F	F	F
0.2498290521810950	0.1248413402520080	0.2386386273748198	T	T	T
0.2913212813871484	0.2080588453932765	0.3225841592626696	T	T	T
0.2499997693913443	0.2499997876146764	0.0000000000000000	F	F	F
0.2916664840813112	0.3333332169946104	0.0784850000000006	F	F	F
0.3333330645283255	0.2916665023046434	0.1569700000000012	F	F	F
0.2498487864532039	0.2498840867343047	0.2386492470758152	T	T	T
0.2912366178544199	0.3330877034474294	0.3226574898523127	T	T	T
0.2499999036342899	0.3749999316845773	0.0000000000000000	F	F	F
0.2916666183242569	0.4583333610645113	0.0784850000000006	F	F	F
0.3333331987712782	0.4166666463745443	0.1569700000000012	F	F	F
0.2497770427726008	0.3748974227241433	0.2386729375899876	T	T	T
0.2914511815090920	0.4580667819035447	0.3226900550808574	T	T	T
0.2500000378772356	0.5000000757544782	0.0000000000000000	F	F	F
0.2916667525672025	0.5833335051344122	0.0784850000000006	F	F	F
0.3333333330142239	0.5416667904444452	0.1569700000000012	F	F	F

0.2499176613231413	0.4999231152047397	0.2387034007790621	T	T	T
0.2915560094817997	0.5832037290876393	0.3224859518573689	T	T	T
0.2500001721201883	0.6250002198243791	0.0000000000000000	F	F	F
0.2916666365475891	0.7083331486791877	0.0784850000000006	F	F	F
0.3333332169946104	0.6666664339892208	0.1569700000000012	F	F	F
0.2500006307467730	0.6249637778098653	0.2386173419722383	T	T	T
0.2916119185932042	0.7082995478457965	0.3225053773264849	T	T	T
0.2500000561005749	0.7499998633691547	0.0000000000000000	F	F	F
0.2916667707905418	0.8333332927490886	0.0784850000000006	F	F	F
0.3333333512375560	0.7916665780591217	0.1569700000000012	F	F	F
0.2499948478536138	0.7499536695966967	0.2385793303129294	T	T	T
0.2917200717681311	0.8333065100964313	0.3223790994959458	T	T	T
0.2500001903435205	0.8750000074390556	0.0000000000000000	F	F	F
0.2916669050334875	0.9583334368189895	0.0784850000000006	F	F	F
0.3333334854805088	0.9166667221290226	0.1569700000000012	F	F	F
0.2500196044200988	0.8749782752523494	0.2385518798125005	T	T	T
0.2917490672293696	0.9583595608015015	0.3223114200509578	T	T	T
0.3750000602194632	0.0000000000000000	0.0000000000000000	F	F	F
0.4166667749094302	0.0833334293799339	0.0784850000000006	F	F	F
0.4583333553564515	0.0416667146899670	0.1569700000000012	F	F	F
0.3750933710460659	0.9999388741267555	0.2386161774934504	T	T	T
0.4167803629495025	0.0833286824640579	0.3225172854512690	T	T	T
0.3750001944624159	0.1250001440699009	0.0000000000000000	F	F	F
0.4166669091523829	0.2083335734498348	0.0784850000000006	F	F	F
0.4583334895993971	0.1666668587598679	0.1569700000000012	F	F	F
0.3749007119095752	0.1247014915998607	0.2386418156036055	T	T	T
0.4165921133571490	0.2081257592888053	0.3223430767296822	T	T	T
0.3750000784427954	0.2499997876146764	0.0000000000000000	F	F	F
0.4166667931327623	0.3333332169946104	0.0784850000000006	F	F	F
0.4583333735797837	0.2916665023046434	0.1569700000000012	F	F	F
0.3746805120188104	0.2495252958130169	0.2384602014340279	T	T	T
0.4160622726305959	0.3330107884714411	0.3207722850817501	T	T	T
0.3749997792182995	0.3749999316845773	0.0000000000000000	F	F	F
0.4166664939082665	0.4583333610645113	0.0784850000000006	F	F	F
0.4583335078227293	0.4166666463745443	0.1569700000000012	F	F	F
0.3745661492158231	0.3749940255493442	0.2385006483150995	T	T	T
0.4164024420854182	0.4583000331686084	0.3224792889465964	T	T	T
0.3749999134612452	0.5000000757544782	0.0000000000000000	F	F	F
0.4166666281512121	0.5833335051344122	0.0784850000000006	F	F	F
0.4583332085982335	0.5416667904444452	0.1569700000000012	F	F	F
0.3746673008646432	0.4999725502296136	0.2387004728532186	T	T	T
0.4163351808655691	0.5830139340483020	0.3227796467078242	T	T	T
0.3750000477041908	0.6250002198243791	0.0000000000000000	F	F	F
0.4166665121315987	0.7083331486791877	0.0784850000000006	F	F	F
0.4583330925786129	0.6666664339892208	0.1569700000000012	F	F	F
0.3749230327552457	0.6249656296448238	0.2387225543466497	T	T	T
0.4164184402558280	0.7081200667166635	0.3226929251611622	T	T	T
0.3749999316845773	0.7499998633691547	0.0000000000000000	F	F	F
0.4166666463745443	0.8333332927490886	0.0784850000000006	F	F	F
0.4583332268215656	0.7916665780591217	0.1569700000000012	F	F	F
0.3749212109078108	0.7499340739059770	0.2386399307636566	T	T	T
0.4166900674650071	0.8331611179694784	0.3225157129776606	T	T	T
0.3750000659275230	0.8750000074390556	0.0000000000000000	F	F	F
0.4166667806174900	0.9583334368189895	0.0784850000000006	F	F	F
0.4583333610645113	0.9166667221290226	0.1569700000000012	F	F	F
0.3750280628175640	0.8749287712427846	0.2386009190323964	T	T	T
0.4167515644985345	0.9582314593148131	0.3223907216181915	T	T	T
0.4999999358034728	0.0000000000000000	0.0000000000000000	F	F	F
0.5416666504934398	0.0833334293799339	0.0784850000000006	F	F	F
0.5833332309404540	0.0416667146899670	0.1569700000000012	F	F	F
0.5001146764406037	-0.0000354641632405	0.2386405205245975	T	T	T
0.5416779589633467	0.0831226807732379	0.3225801408880986	T	T	T
0.5000000700464184	0.1250001440699009	0.0000000000000000	F	F	F
0.5416667847363854	0.2083335734498348	0.0784850000000006	F	F	F
0.5833333651834067	0.1666668587598679	0.1569700000000012	F	F	F
0.5000871273667189	0.1248659791057875	0.2386402711730189	T	T	T
0.5417475510206367	0.2080757688027575	0.3226316431805186	T	T	T
0.4999999540268050	0.2499997876146764	0.0000000000000000	F	F	F
0.5416666687167719	0.3333332169946104	0.0784850000000006	F	F	F
0.5833332491637861	0.2916665023046434	0.1569700000000012	F	F	F
0.4999095111027022	0.2495485090245844	0.2384754281319517	T	T	T
0.5419045589177470	0.3329942228902530	0.3209803635689751	T	T	T
0.5000000882697506	0.3749999316845773	0.0000000000000000	F	F	F
0.5416668029597176	0.4583333610645113	0.0784850000000006	F	F	F
0.5833333834067389	0.4166666463745443	0.1569700000000012	F	F	F

0.4999796146197897	0.3748463291608421	0.2384216665198495	T	T	T
0.5419164126642139	0.4588103015919364	0.3208911282980293	T	T	T
0.5000002225126963	0.5000000757544782	0.0000000000000000	F	F	F
0.5416669372026632	0.5833335051344122	0.0784850000000006	F	F	F
0.5833335176496846	0.5416667904444452	0.1569700000000012	F	F	F
0.4996045533296407	0.4998413846770365	0.2384244822899358	T	T	T
0.5415059624148685	0.5831875477849896	0.3222096348440098	T	T	T
0.4999999232882004	0.6250002198243791	0.0000000000000000	F	F	F
0.5416663877156012	0.7083331486791877	0.0784850000000006	F	F	F
0.5833334016300711	0.6666664339892208	0.1569700000000012	F	F	F
0.4996801915616860	0.6247142891500678	0.2385008684767442	T	T	T
0.5410657113141456	0.7078972223028158	0.3213596860875861	T	T	T
0.4999998072685798	0.7499998633691547	0.0000000000000000	F	F	F
0.5416665219585468	0.8333332927490886	0.0784850000000006	F	F	F
0.5833331024055681	0.7916665780591217	0.1569700000000012	F	F	F
0.4997562455444886	0.7500470995080668	0.2385614635306300	T	T	T
0.5415856362143080	0.8331180398390396	0.3226690644454406	T	T	T
0.4999999415115326	0.8750000074390556	0.0000000000000000	F	F	F
0.5416666562014996	0.9583334368189895	0.0784850000000006	F	F	F
0.5833332366485138	0.9166667221290226	0.1569700000000012	F	F	F
0.4999557659731867	0.8749970293311599	0.2386881214581759	T	T	T
0.5417190062251495	0.9581048643826521	0.3225685024144985	T	T	T
0.6249998113874753	0.0000000000000000	0.0000000000000000	F	F	F
0.6666665260774423	0.0833334293799339	0.0784850000000006	F	F	F
0.7083335399919122	0.0416667146899670	0.1569700000000012	F	F	F
0.6250390619241086	0.9999824184524880	0.2386490046625398	T	T	T
0.6666286959842717	0.0831649814728150	0.3225055204125635	T	T	T
0.6249999456304209	0.1250001440699009	0.0000000000000000	F	F	F
0.6666666603203879	0.2083335734498348	0.0784850000000006	F	F	F
0.7083332407674092	0.1666668587598679	0.1569700000000012	F	F	F
0.6250342316743940	0.1249686606333395	0.2386508829008505	T	T	T
0.6666880472483009	0.2082066151611774	0.3225430017929253	T	T	T
0.6249998296108075	0.2499997876146764	0.0000000000000000	F	F	F
0.6666665443007744	0.3333332169946104	0.0784850000000006	F	F	F
0.7083331247477958	0.2916665023046434	0.1569700000000012	F	F	F
0.6250423859682138	0.2499159218884653	0.2386690675162204	T	T	T
0.6667904355616199	0.3330995046328321	0.3226938121035663	T	T	T
0.6249999638537531	0.3749999316845773	0.0000000000000000	F	F	F
0.6666667785437201	0.4583333610645113	0.0784850000000006	F	F	F
0.7083332589907414	0.4166666463745443	0.1569700000000012	F	F	F
0.6254005312314302	0.3750412643348878	0.2385176758482021	T	T	T
0.6667494175318196	0.4581718322814037	0.3225674783562759	T	T	T
0.6250000980967059	0.5000000757544782	0.0000000000000000	F	F	F
0.6666668127866728	0.5833335051344122	0.0784850000000006	F	F	F
0.7083333932336870	0.5416667904444452	0.1569700000000012	F	F	F
0.6253815142178027	0.5000325875190899	0.2384933369486087	T	T	T
0.6669416016820930	0.5832143249309881	0.3226783964209753	T	T	T
0.6250002323396515	0.6250002198243791	0.0000000000000000	F	F	F
0.6666666967670594	0.7083331486791877	0.0784850000000006	F	F	F
0.7083332772140736	0.6666664339892208	0.1569700000000012	F	F	F
0.6248521839929729	0.6245217587169741	0.2384108681200679	T	T	T
0.6668922988924258	0.7078489071292807	0.3206321589230066	T	T	T
0.6250001163200380	0.7499998633691547	0.0000000000000000	F	F	F
0.6666668310100050	0.8333332927490886	0.0784850000000006	F	F	F
0.7083334114570263	0.7916665780591217	0.1569700000000012	F	F	F
0.6248759724296926	0.7498823260003618	0.2384958279942062	T	T	T
0.6667090539083297	0.8333896238759601	0.3215585324501285	T	T	T
0.6249998170955351	0.8750000074390556	0.0000000000000000	F	F	F
0.6666665317855021	0.9583334368189895	0.0784850000000006	F	F	F
0.7083335456999720	0.9166667221290226	0.1569700000000012	F	F	F
0.6248604960264844	0.8750581152926629	0.2385953505194637	T	T	T
0.6666372320785547	0.9582008925678018	0.3227002658130303	T	T	T
0.7500001204389335	0.0000000000000000	0.0000000000000000	F	F	F
0.7916668351289005	0.0833334293799339	0.0784850000000006	F	F	F
0.8333334155759147	0.0416667146899670	0.1569700000000012	F	F	F
0.7499382312581802	0.9998891188524731	0.2386769423639144	T	T	T
0.7915934343412452	0.0831438716563308	0.3224414190428387	T	T	T
0.7500002546818791	0.1250001440699009	0.0000000000000000	F	F	F
0.7916669693718461	0.2083335734498348	0.0784850000000006	F	F	F
0.8333335498188674	0.1666668587598679	0.1569700000000012	F	F	F
0.7499537632321700	0.1249197072285666	0.2386155500110834	T	T	T
0.7916536056302073	0.2082119614217321	0.3223871695098140	T	T	T
0.7499997051948100	0.2499997876146764	0.0000000000000000	F	F	F
0.7916664198847769	0.3333332169946104	0.0784850000000006	F	F	F
0.8333334337992468	0.2916665023046434	0.1569700000000012	F	F	F

0.7499843479870550	0.2499612492866959	0.2386356909889525	T	T	T
0.7917197570203822	0.3332595358678573	0.3225340769887307	T	T	T
0.7499998394377627	0.3749999316845773	0.0000000000000000	F	F	F
0.7916665541277297	0.4583333610645113	0.0784850000000006	F	F	F
0.8333331345747439	0.4166666463745443	0.1569700000000012	F	F	F
0.7500575238707705	0.3749138245551205	0.2386363654525276	T	T	T
0.7915754525206499	0.4581587126807783	0.3225466423424608	T	T	T
0.7499999736807084	0.5000000757544782	0.0000000000000000	F	F	F
0.7916666883706753	0.5833335051344122	0.0784850000000006	F	F	F
0.8333332688176966	0.5416667904444452	0.1569700000000012	F	F	F
0.7502134104714643	0.5000834507870159	0.2387332479968293	T	T	T
0.7916288648789708	0.5831970843790377	0.3227427061860561	T	T	T
0.7500001079236540	0.6250002198243791	0.0000000000000000	F	F	F
0.7916665723510619	0.7083331486791877	0.0784850000000006	F	F	F
0.8333331527980832	0.6666664339892208	0.1569700000000012	F	F	F
0.7501142496041626	0.6250078201431669	0.2386747733621870	T	T	T
0.7919303642556783	0.7081211198288974	0.3226937933623913	T	T	T
0.7499999919040405	0.7499998633691547	0.0000000000000000	F	F	F
0.7916667065940075	0.8333332927490886	0.0784850000000006	F	F	F
0.8333332870410288	0.7916665780591217	0.1569700000000012	F	F	F
0.7503778223768534	0.7500121506151234	0.2384590072987364	T	T	T
0.7917386076035117	0.8331887548767974	0.3223983989482635	T	T	T
0.7500001261469933	0.8750000074390556	0.0000000000000000	F	F	F
0.7916668408369603	0.9583334368189895	0.0784850000000006	F	F	F
0.8333334212839745	0.9166667221290226	0.1569700000000012	F	F	F
0.7501453085015782	0.8749889972300311	0.2385807765055055	T	T	T
0.7916423881885222	0.9582343063834189	0.3226224355305112	T	T	T
0.8749999960229360	0.0000000000000000	0.0000000000000000	F	F	F
0.9166667107129030	0.0833334293799339	0.0784850000000006	F	F	F
0.9583332911599243	0.0416667146899670	0.1569700000000012	F	F	F
0.8749720624035354	0.9999502519087724	0.2386391521096175	T	T	T
0.9166188372845465	0.0831741652898735	0.3224583345598327	T	T	T
0.8750001302658816	0.1250001440699009	0.0000000000000000	F	F	F
0.9166668449558486	0.2083335734498348	0.0784850000000006	F	F	F
0.9583334254028699	0.1666668587598679	0.1569700000000012	F	F	F
0.8749505718534653	0.1248911393785813	0.2386181573556396	T	T	T
0.9166050417780729	0.2081912189975646	0.3223589063039646	T	T	T
0.8750000142462682	0.2499997876146764	0.0000000000000000	F	F	F
0.9166667289362351	0.3333332169946104	0.0784850000000006	F	F	F
0.9583333093832564	0.2916665023046434	0.1569700000000012	F	F	F
0.8749579963701309	0.2499177473891950	0.2385651985208573	T	T	T
0.9166318460100896	0.3332462752180108	0.3224064855720930	T	T	T
0.8750001484892138	0.3749999316845773	0.0000000000000000	F	F	F
0.9166668631791808	0.4583333610645113	0.0784850000000006	F	F	F
0.9583334436262021	0.4166666463745443	0.1569700000000012	F	F	F
0.8749694415973593	0.3749396165821719	0.2385974503028773	T	T	T
0.9166363230399823	0.4582807592436070	0.3224221466157684	T	T	T
0.8749998492647180	0.5000000757544782	0.0000000000000000	F	F	F
0.9166665639546849	0.5833335051344122	0.0784850000000006	F	F	F
0.9583335778691477	0.5416667904444452	0.1569700000000012	F	F	F
0.8749683368567291	0.4999517293298856	0.2386276743023629	T	T	T
0.9166279320506446	0.5832674366602199	0.3223570016198155	T	T	T
0.8749999835076636	0.6250002198243791	0.0000000000000000	F	F	F
0.9166664479350644	0.7083331486791877	0.0784850000000006	F	F	F
0.9583330283820857	0.6666664339892208	0.1569700000000012	F	F	F
0.8749750495258450	0.6250276915789992	0.2387162066465678	T	T	T
0.9167533707422951	0.7082912422057454	0.3226371874526821	T	T	T
0.8749998674880501	0.7499998633691547	0.0000000000000000	F	F	F
0.9166665821780171	0.8333332927490886	0.0784850000000006	F	F	F
0.9583331626250313	0.7916665780591217	0.1569700000000012	F	F	F
0.8750887348840704	0.7499535068194016	0.2386595878732043	T	T	T
0.9165882737032998	0.8331782856925828	0.3225688165617523	T	T	T
0.8750000017309958	0.8750000074390556	0.0000000000000000	F	F	F
0.9166667164209628	0.9583334368189895	0.0784850000000006	F	F	F
0.9583332968679841	0.9166667221290226	0.1569700000000012	F	F	F
0.8751641622377656	0.8750362328542953	0.2386358199100282	T	T	T
0.9165288274380261	0.9581580387932328	0.3225436102009878	T	T	T
0.6994786449979095	0.7769435351650361	0.4251339651557873	T	T	T
0.5925824469738044	0.6648869947236522	0.4250252711566680	T	T	T
0.5873646732241874	0.7716684494824905	0.4270344394621785	T	T	T
0.5729234793645779	0.4156771226024441	0.4248400269633936	T	T	T
0.4640355722601562	0.3052847964897289	0.4242077087253637	T	T	T
0.4625626301049505	0.4141888675865074	0.4245008744203554	T	T	T
0.6642632637258762	0.7049553941030896	0.4244220166773797	T	T	T
0.6592556567862219	0.8083350483226445	0.4267531407933751	T	T	T

```
  0.5558254023507070  0.6998832584293700  0.4264550857239910   T   T   T
  0.5358867436557685  0.3437675003947179  0.4247090046641698   T   T   T
  0.5344532468996955  0.4490647155753615  0.4248698398654064   T   T   T
  0.4291545237911188  0.3423269702056898  0.4242731573733992   T   T   T
  0.6917061399308360  0.6814506593172319  0.4225475499640098   T   T   T
  0.5645156616039663  0.6046895049740046  0.4246511321905811   T   T   T
  0.5048148121031450  0.6725653473217027  0.4262216700461778   T   T   T
  0.5551797657392176  0.7994855087088044  0.4278876245714536   T   T   T
  0.6829371798863197  0.8593418611802759  0.4269920840849530   T   T   T
  0.7595271072569304  0.8089970266785543  0.4245896818103122   T   T   T
  0.5619405149252060  0.3187647756047928  0.4243231591774929   T   T   T
  0.4346000554511448  0.2451874743747922  0.4238783957326131   T   T   T
  0.3780987758416799  0.3162844038636561  0.4235642412858199   T   T   T
  0.4319501731491018  0.4436962598564295  0.4245804019139115   T   T   T
  0.5592591459657918  0.5001466673767569  0.4246313084022399   T   T   T
  0.6330294623487451  0.4463215410586702  0.4250868661538686   T   T   T
```

Borazine dimer adsorbed on Au(111), dimer II

```
    23.0697829620000014    0.0000000000000000    0.0000000000000000
   -11.5348914810000007   19.9790169730000002    0.0000000000000000
     0.0000000000000000    0.0000000000000000   30.0000000000000000
   Au   B    N    H
   320     6     6    12
Selective dynamics
Direct
  0.0000000000000000  0.0000000000000000  0.0000000000000000   F   F   F
  0.0416667146899670  0.0833334293799339  0.0784850000000006   F   F   F
  0.0833332951369883  0.0416667146899670  0.1569700000000012   F   F   F
  0.0000386306477370  0.0000170664558566  0.2385698720846657   T   T   T
  0.0416089149678961  0.0832871233435406  0.3224293160256608   T   T   T
  0.0000001342429456  0.1250001440699009  0.0000000000000000   F   F   F
  0.0416668489329126  0.2083335734498348  0.0784850000000006   F   F   F
  0.0833334293799339  0.1666668587598679  0.1569700000000012   F   F   F
  0.0000053299204368  0.1250060024355980  0.2385712140527525   T   T   T
  0.0416077587701288  0.2082943639849962  0.3224914544374957   T   T   T
  0.0000000182233322  0.2499997876146764  0.0000000000000000   F   F   F
  0.0416667329132991  0.3333332169946104  0.0784850000000006   F   F   F
  0.0833333133603205  0.2916665023046434  0.1569700000000012   F   F   F
  0.0000316525286784  0.2500000010223495  0.2385906300893831   T   T   T
  0.0416926258513331  0.3333381824644399  0.3223842374239829   T   T   T
  0.0000001524662778  0.3749999316845773  0.0000000000000000   F   F   F
  0.0416668671562448  0.4583333610645113  0.0784850000000006   F   F   F
  0.0833334476032661  0.4166666463745443  0.1569700000000012   F   F   F
  0.0000299193156970  0.3749757588448215  0.2385778313843589   T   T   T
  0.0417015115858202  0.4583108135257001  0.3224323782192487   T   T   T
  0.9999997248487205  0.5000000757544782  0.0000000000000000   F   F   F
  0.0416665679317489  0.5833335051344122  0.0784850000000006   F   F   F
  0.0833335818462118  0.5416667904444452  0.1569700000000012   F   F   F
  0.0000119841974980  0.4999605717836347  0.2385700617341981   T   T   T
  0.0417206905179474  0.5833120037157682  0.3223959633162752   T   T   T
  0.9999998590916661  0.6250002198243791  0.0000000000000000   F   F   F
  0.0416664519121284  0.7083331486791877  0.0784850000000006   F   F   F
  0.0833330323591497  0.6666664339892208  0.1569700000000012   F   F   F
  0.0000280631453650  0.6249844706761615  0.2385645502352554   T   T   T
  0.0417687498490671  0.7083161238885606  0.3223647164686804   T   T   T
  0.9999997430720526  0.7499998633691547  0.0000000000000000   F   F   F
  0.0416665861550811  0.8333332927490886  0.0784850000000006   F   F   F
  0.0833331666020953  0.7916665780591217  0.1569700000000012   F   F   F
  0.0000294896976386  0.7499784121932516  0.2385531422718649   T   T   T
  0.0417188364327587  0.8332952603734188  0.3223843597845815   T   T   T
  0.0000000057080598  0.8750000074390556  0.0000000000000000   F   F   F
  0.0416667203980268  0.9583334368189895  0.0784850000000006   F   F   F
  0.0833333008450481  0.9166667221290226  0.1569700000000012   F   F   F
  0.0000259973922884  0.8750021844097797  0.2385491288807623   T   T   T
  0.0416700562060952  0.9582855675970581  0.3223783453548144   T   T   T
  0.1249998755840025  0.0000000000000000  0.0000000000000000   F   F   F
  0.1666665902739695  0.0833334293799339  0.0784850000000006   F   F   F
  0.2083331707209908  0.0416667146899670  0.1569700000000012   F   F   F
  0.1250334116036166  0.0000189631215267  0.2386194317910958   T   T   T
  0.1665971494415941  0.0832730008639330  0.3225319629464752   T   T   T
  0.1250000098269552  0.1250001440699009  0.0000000000000000   F   F   F
  0.1666667245169222  0.2083335734498348  0.0784850000000006   F   F   F
  0.2083333049639364  0.1666668587598679  0.1569700000000012   F   F   F
  0.1249837080829655  0.1250067051670186  0.2386720812904533   T   T   T
  0.1665063586988086  0.2082690912798515  0.3227145088917939   T   T   T
```

0.1249998938073347	0.2499997876146764	0.0000000000000000	F	F	F
0.1666666084973016	0.3333332169946104	0.0784850000000006	F	F	F
0.2083331889443230	0.2916665023046434	0.1569700000000012	F	F	F
0.1249679512871026	0.2499575007549049	0.2386699683677998	T	T	T
0.1666283972666576	0.3332568399588361	0.3226235561515183	T	T	T
0.1250000280502874	0.3749999316845773	0.0000000000000000	F	F	F
0.1666667427402544	0.4583333610645113	0.0784850000000006	F	F	F
0.2083333231872686	0.4166666463745443	0.1569700000000012	F	F	F
0.1250424893280548	0.3749888731953435	0.2386423836951643	T	T	T
0.1667069546605455	0.4583302036865209	0.3225603734168396	T	T	T
0.1250001622932331	0.5000000757544782	0.0000000000000000	F	F	F
0.1666668769832000	0.5833335051344122	0.0784850000000006	F	F	F
0.2083334574302214	0.5416667904444452	0.1569700000000012	F	F	F
0.1250120125607395	0.4999620972842820	0.2386259581571761	T	T	T
0.1666978097188219	0.5832850262588114	0.3225014007627608	T	T	T
0.1250002965361787	0.6250002198243791	0.0000000000000000	F	F	F
0.1666667609635866	0.7083331486791877	0.0784850000000006	F	F	F
0.2083333414106079	0.6666664339892208	0.1569700000000012	F	F	F
0.1250125592279338	0.6249506365925894	0.2385838889371117	T	T	T
0.1667546443486833	0.7083194613480004	0.3223326642468579	T	T	T
0.1249997470491166	0.7499998633691547	0.0000000000000000	F	F	F
0.1666664617390836	0.8333332927490886	0.0784850000000006	F	F	F
0.2083334756535535	0.7916665780591217	0.1569700000000012	F	F	F
0.1250409215678483	0.7499920633952669	0.2385417470750013	T	T	T
0.1667522824035617	0.8333409713956782	0.3223842749047085	T	T	T
0.1249998812920623	0.8750000074390556	0.0000000000000000	F	F	F
0.1666665959820293	0.9583334368189895	0.0784850000000006	F	F	F
0.2083331764290506	0.9166667221290226	0.1569700000000012	F	F	F
0.1250149090552297	0.8750053335207481	0.2385780157821667	T	T	T
0.1666412814433432	0.9582347689561923	0.3225028075938766	T	T	T
0.2500001846354607	0.0000000000000000	0.0000000000000000	F	F	F
0.2916668993254277	0.0833334293799339	0.0784850000000006	F	F	F
0.3333334797724490	0.0416667146899670	0.1569700000000012	F	F	F
0.2499268574692073	0.9999143228824598	0.2386198681116336	T	T	T
0.2914430055348309	0.0831744392261397	0.3225906187481656	T	T	T
0.2499998854109577	0.1250001440699009	0.0000000000000000	F	F	F
0.2916666001009247	0.2083335734498348	0.0784850000000006	F	F	F
0.3333336140153946	0.1666668587598679	0.1569700000000012	F	F	F
0.2499215074531732	0.1249303344762971	0.2387173928739616	T	T	T
0.2913737839401276	0.2082334734746714	0.3227237737901724	T	T	T
0.2499997693913443	0.2499997876146764	0.0000000000000000	F	F	F
0.2916664840813112	0.3333332169946104	0.0784850000000006	F	F	F
0.3333330645283255	0.2916665023046434	0.1569700000000012	F	F	F
0.2497088614630870	0.2497808944079073	0.2386839635148557	T	T	T
0.2914501518127459	0.3330250020788116	0.3226905727418584	T	T	T
0.2499999036342899	0.3749999316845773	0.0000000000000000	F	F	F
0.2916666183242569	0.4583333610645113	0.0784850000000006	F	F	F
0.3333331987712782	0.4166666463745443	0.1569700000000012	F	F	F
0.2498309607335171	0.3748623572484606	0.2387326396403029	T	T	T
0.2915035765850779	0.4581506522743228	0.3224667713013010	T	T	T
0.2500000378772356	0.5000000757544782	0.0000000000000000	F	F	F
0.2916667525672025	0.5833335051344122	0.0784850000000006	F	F	F
0.3333333330142239	0.5416667904444452	0.1569700000000012	F	F	F
0.2499189335101808	0.5000003862750636	0.2386772993148299	T	T	T
0.2916523489491263	0.5833128775006903	0.3226314537800222	T	T	T
0.2500001721201883	0.6250002198243791	0.0000000000000000	F	F	F
0.2916666365475891	0.7083331486791877	0.0784850000000006	F	F	F
0.3333332169946104	0.6666664339892208	0.1569700000000012	F	F	F
0.2499505659194408	0.6250052609075997	0.2386527449488306	T	T	T
0.2917750178437022	0.7083057406662621	0.3224770819204379	T	T	T
0.2500000561005749	0.7499998633691547	0.0000000000000000	F	F	F
0.2916667707905418	0.8333332927490886	0.0784850000000006	F	F	F
0.3333333512375560	0.7916665780591217	0.1569700000000012	F	F	F
0.2500802039896866	0.7500166169403738	0.2386063406710545	T	T	T
0.2917928862614041	0.8334005587513509	0.3223238902957940	T	T	T
0.2500001903435205	0.8750000074390556	0.0000000000000000	F	F	F
0.2916669050334875	0.9583334368189895	0.0784850000000006	F	F	F
0.3333334854805088	0.9166667221290226	0.1569700000000012	F	F	F
0.2500380329671115	0.8750135476199568	0.2386066440277506	T	T	T
0.2917488355026619	0.9584295483299539	0.3225025965144908	T	T	T
0.3750000602194632	0.0000000000000000	0.0000000000000000	F	F	F
0.4166667749094302	0.0833334293799339	0.0784850000000006	F	F	F
0.4583333553564515	0.0416667146899670	0.1569700000000012	F	F	F
0.3749739829941486	0.9998302925824951	0.2386718633916579	T	T	T
0.4166678465537314	0.0832958832306069	0.3226042296244139	T	T	T

0.3750001944624159	0.1250001440699009	0.0000000000000000	F	F	F
0.4166669091523829	0.2083335734498348	0.0784850000000006	F	F	F
0.4583334895993971	0.1666668587598679	0.1569700000000012	F	F	F
0.3747975445011513	0.1245845574016428	0.2385143282130386	T	T	T
0.4161647310559586	0.2080513798335012	0.3210451462938438	T	T	T
0.3750000784427954	0.2499997876146764	0.0000000000000000	F	F	F
0.4166667931327623	0.3333332169946104	0.0784850000000006	F	F	F
0.4583333735797837	0.2916665023046434	0.1569700000000012	F	F	F
0.3746596958675752	0.2498507677613118	0.2384579990515392	T	T	T
0.4164943741635561	0.3331910729200613	0.3222056588523463	T	T	T
0.3749997792182995	0.3749999316845773	0.0000000000000000	F	F	F
0.4166664939082665	0.4583333610645113	0.0784850000000006	F	F	F
0.4583335078227293	0.4166666463745443	0.1569700000000012	F	F	F
0.3745382088351126	0.3745855656443224	0.2383688769931345	T	T	T
0.4164994236038203	0.4578031854113007	0.3207924367968201	T	T	T
0.3749999134612452	0.5000000757544782	0.0000000000000000	F	F	F
0.4166666281512121	0.5833335051344122	0.0784850000000006	F	F	F
0.4583332085982335	0.5416667904444452	0.1569700000000012	F	F	F
0.3747966707555253	0.5000156742285465	0.2384812165172307	T	T	T
0.4165441751779467	0.5834790143240285	0.3213880453874596	T	T	T
0.3750000477041908	0.6250002198243791	0.0000000000000000	F	F	F
0.4166665121315987	0.7083331486791877	0.0784850000000006	F	F	F
0.4583330925786129	0.6666664339892208	0.1569700000000012	F	F	F
0.3748331232227297	0.6252459379712622	0.2385186767543628	T	T	T
0.4167010890590344	0.7083806334779514	0.3226703739535460	T	T	T
0.3749999316845773	0.7499998633691547	0.0000000000000000	F	F	F
0.4166666463745443	0.8333332927490886	0.0784850000000006	F	F	F
0.4583332268215656	0.7916665780591217	0.1569700000000012	F	F	F
0.3750723793266703	0.7501217265705500	0.2386529089926613	T	T	T
0.4167520771038326	0.8333828271924597	0.3225804277385302	T	T	T
0.3750000659275230	0.8750000074390556	0.0000000000000000	F	F	F
0.4166667806174900	0.9583334368189895	0.0784850000000006	F	F	F
0.4583333610645113	0.9166667221290226	0.1569700000000012	F	F	F
0.3751415738148672	0.8750525403381236	0.2386383877229107	T	T	T
0.4167807425538155	0.9584594499890401	0.3225765167687088	T	T	T
0.4999999358034728	0.0000000000000000	0.0000000000000000	F	F	F
0.5416666504934398	0.0833334293799339	0.0784850000000006	F	F	F
0.5833332309404540	0.0416667146899670	0.1569700000000012	F	F	F
0.5000749452103553	0.9999719838172234	0.2386561661266043	T	T	T
0.5417459826375985	0.0832801594229138	0.3226806311673485	T	T	T
0.5000000700464184	0.1250001440699009	0.0000000000000000	F	F	F
0.5416667847363854	0.2083335734498348	0.0784850000000006	F	F	F
0.5833333651834067	0.1666668587598679	0.1569700000000012	F	F	F
0.4999243790043509	0.1246983204766150	0.2385633138134714	T	T	T
0.5418808776702470	0.2081875671301957	0.3213455627207822	T	T	T
0.4999999540268050	0.2499997876146764	0.0000000000000000	F	F	F
0.5416666687167719	0.3333332169946104	0.0784850000000006	F	F	F
0.5833332491637861	0.2916665023046434	0.1569700000000012	F	F	F
0.5000184699329721	0.2499415007501548	0.2384492930543220	T	T	T
0.5419514779386441	0.3339026649559689	0.3208336089399638	T	T	T
0.5000000882697506	0.3749999316845773	0.0000000000000000	F	F	F
0.5416668029597176	0.4583333610645113	0.0784850000000006	F	F	F
0.5833333834067389	0.4166666463745443	0.1569700000000012	F	F	F
0.4998700544234019	0.3751165880308315	0.2383855357527797	T	T	T
0.5420197431461096	0.4585323675802094	0.3222222461944327	T	T	T
0.5000002225126963	0.5000000757544782	0.0000000000000000	F	F	F
0.5416669372026632	0.5833335051344122	0.0784850000000006	F	F	F
0.5833335176496846	0.5416667904444452	0.1569700000000012	F	F	F
0.5001735879922100	0.5000621035683579	0.2383546924752446	T	T	T
0.5422064025833808	0.5836771291369420	0.3209809369314753	T	T	T
0.4999999232882004	0.6250002198243791	0.0000000000000000	F	F	F
0.5416663877156012	0.7083331486791877	0.0784850000000006	F	F	F
0.5833334016300711	0.6666664339892208	0.1569700000000012	F	F	F
0.5001167685780299	0.6252672882191270	0.2384838381082223	T	T	T
0.5417968496850445	0.7085176223528915	0.3226213521347526	T	T	T
0.4999998072685798	0.7499998633691547	0.0000000000000000	F	F	F
0.5416665219585468	0.8333332927490886	0.0784850000000006	F	F	F
0.5833331024055681	0.7916665780591217	0.1569700000000012	F	F	F
0.5000263039564392	0.7500932450481249	0.2387012744504634	T	T	T
0.5416525021114263	0.8332953190698629	0.3226068167340549	T	T	T
0.4999999415115326	0.8750000074390556	0.0000000000000000	F	F	F
0.5416666562014996	0.9583334368189895	0.0784850000000006	F	F	F
0.5833332366485138	0.9166667221290226	0.1569700000000012	F	F	F
0.5000611393584641	0.8750222962984099	0.2386554728319292	T	T	T
0.5416543908636311	0.9582954227085931	0.3225923528339276	T	T	T

0.6249998113874753	0.0000000000000000	0.0000000000000000	F	F	F
0.6666665260774423	0.0833334293799339	0.0784850000000006	F	F	F
0.7083335399919122	0.0416667146899670	0.1569700000000012	F	F	F
0.6249761768348793	0.9999881425351970	0.2386461811762942	T	T	T
0.6666569844973881	0.0833309779446537	0.3224993838677677	T	T	T
0.6249999456304209	0.1250001440699009	0.0000000000000000	F	F	F
0.6666666603203879	0.2083335734498348	0.0784850000000006	F	F	F
0.7083332407674092	0.1666668587598679	0.1569700000000012	F	F	F
0.6250155090984976	0.1249998599663626	0.2386750213897391	T	T	T
0.6667668015526491	0.2082911263691476	0.3226495647150775	T	T	T
0.6249998296108075	0.2499997876146764	0.0000000000000000	F	F	F
0.6666665443007744	0.3333332169946104	0.0784850000000006	F	F	F
0.7083331247477958	0.2916665023046434	0.1569700000000012	F	F	F
0.6253027007805015	0.2501306456836418	0.2385539243080913	T	T	T
0.6668223559793783	0.3334660628669788	0.3224165974145379	T	T	T
0.6249999638537531	0.3749999316845773	0.0000000000000000	F	F	F
0.6666666785437201	0.4583333610645113	0.0784850000000006	F	F	F
0.7083332589907414	0.4166666463745443	0.1569700000000012	F	F	F
0.6254433880073987	0.3753290164759124	0.2385041148961546	T	T	T
0.6670133615967371	0.4587095157677564	0.3227272068619101	T	T	T
0.6250000980967059	0.5000000757544782	0.0000000000000000	F	F	F
0.6666668127866728	0.5833335051344122	0.0784850000000006	F	F	F
0.7083333932336870	0.5416667904444452	0.1569700000000012	F	F	F
0.6252957765683381	0.5001466602282129	0.2386491593165870	T	T	T
0.6669947500305841	0.5834698308017335	0.3227025792763917	T	T	T
0.6250002323396515	0.6250002198243791	0.0000000000000000	F	F	F
0.6666666967670594	0.7083331486791877	0.0784850000000006	F	F	F
0.7083332772140736	0.6666664339892208	0.1569700000000012	F	F	F
0.6255611962235981	0.6252596226729162	0.2384857939010777	T	T	T
0.6669188328967058	0.7085318859783961	0.3225801858097385	T	T	T
0.6250001163200380	0.7499998633691547	0.0000000000000000	F	F	F
0.6666668310100050	0.8333332927490886	0.0784850000000006	F	F	F
0.7083334114570263	0.7916665780591217	0.1569700000000012	F	F	F
0.6250858861526419	0.7500658312957227	0.2386864138004693	T	T	T
0.6667072996476834	0.8333331480377165	0.3225429841602457	T	T	T
0.6249998170955351	0.8750000074390556	0.0000000000000000	F	F	F
0.6666665317855021	0.9583334368189895	0.0784850000000006	F	F	F
0.7083335456999720	0.9166667221290226	0.1569700000000012	F	F	F
0.6249711934953646	0.8749404594102754	0.2386454490913155	T	T	T
0.6666471649255907	0.9582956311162443	0.3223739685376169	T	T	T
0.7500001204389335	0.0000000000000000	0.0000000000000000	F	F	F
0.7916668351289005	0.0833334293799339	0.0784850000000006	F	F	F
0.8333334155759147	0.0416667146899670	0.1569700000000012	F	F	F
0.7499570168003625	0.9999731393906468	0.2385798093895336	T	T	T
0.7916787872914868	0.0833336096048046	0.3223733936540477	T	T	T
0.7500002546818791	0.1250001440699009	0.0000000000000000	F	F	F
0.7916669693718461	0.2083335734498348	0.0784850000000006	F	F	F
0.8333335498188674	0.1666668587598679	0.1569700000000012	F	F	F
0.7499751279881895	0.1250137483890346	0.2386407471998124	T	T	T
0.7917129498835961	0.2083538748034177	0.3225376602763446	T	T	T
0.7499997051948100	0.2499997876146764	0.0000000000000000	F	F	F
0.7916664198847769	0.3333332169946104	0.0784850000000006	F	F	F
0.8333334337992468	0.2916665023046434	0.1569700000000012	F	F	F
0.7500444590014702	0.2499729265819828	0.2386536277342459	T	T	T
0.7916264429300607	0.3332860701867816	0.3225510474765942	T	T	T
0.7499998394377627	0.3749999316845773	0.0000000000000000	F	F	F
0.7916665541277297	0.4583333610645113	0.0784850000000006	F	F	F
0.8333331345747439	0.4166666463745443	0.1569700000000012	F	F	F
0.7502350909372982	0.3751273005716033	0.2386677601309950	T	T	T
0.7917031196036455	0.4583594718488516	0.3226089285413223	T	T	T
0.7499999736807084	0.5000000757544782	0.0000000000000000	F	F	F
0.7916666883706753	0.5833335051344122	0.0784850000000006	F	F	F
0.8333332688176966	0.5416667904444452	0.1569700000000012	F	F	F
0.7502009377874564	0.5001429546571122	0.2386924350830632	T	T	T
0.7918908093212385	0.5834115307399731	0.3227292083333799	T	T	T
0.7500001079236540	0.6250002198243791	0.0000000000000000	F	F	F
0.7916665723510619	0.7083331486791877	0.0784850000000006	F	F	F
0.8333331527980832	0.6666664339892208	0.1569700000000012	F	F	F
0.7502370595777971	0.6250353811246343	0.2387101600861512	T	T	T
0.7917474118448931	0.7083714326250881	0.3225402951935143	T	T	T
0.7499999919040405	0.7499998633691547	0.0000000000000000	F	F	F
0.7916667065940075	0.8333332927490886	0.0784850000000006	F	F	F
0.8333332870410288	0.7916665780591217	0.1569700000000012	F	F	F
0.7501611736359571	0.7501037605785619	0.2386308619138324	T	T	T
0.7917569597742334	0.8334107355110630	0.3225168917883461	T	T	T

```
  0.7500001261469933  0.8750000074390556  0.0000000000000000   F   F   F
  0.7916668408369603  0.9583334368189895  0.0784850000000006   F   F   F
  0.8333334212839745  0.9166667221290226  0.1569700000000012   F   F   F
  0.7499916878155437  0.8749849635228457  0.2386305989146958   T   T   T
  0.7916835696663427  0.9583351133900730  0.3224133703857353   T   T   T
  0.8749999960229360  0.0000000000000000  0.0000000000000000   F   F   F
  0.9166667107129030  0.0833334293799339  0.0784850000000006   F   F   F
  0.9583332911599243  0.0416667146899670  0.1569700000000012   F   F   F
  0.8749964664992891  0.9999975721163394  0.2385772136852126   T   T   T
  0.9166551778834086  0.0833266192105344  0.3223931917645034   T   T   T
  0.8750001302658816  0.1250001440699009  0.0000000000000000   F   F   F
  0.9166668449558486  0.2083335734498348  0.0784850000000006   F   F   F
  0.9583334254028699  0.1666668587598679  0.1569700000000012   F   F   F
  0.8749951719431653  0.1249963245445485  0.2385729441597407   T   T   T
  0.9166778513127318  0.2083225803430096  0.3224144741286931   T   T   T
  0.8750000142462682  0.2499997876146764  0.0000000000000000   F   F   F
  0.9166667289362351  0.3333332169946104  0.0784850000000006   F   F   F
  0.9583333093832564  0.2916665023046434  0.1569700000000012   F   F   F
  0.8749849293914499  0.2499678377799479  0.2386036810400413   T   T   T
  0.9166697622440636  0.3333216348962348  0.3224371032879965   T   T   T
  0.8750001484892138  0.3749999316845773  0.0000000000000000   F   F   F
  0.9166668631791808  0.4583333610645113  0.0784850000000006   F   F   F
  0.9583334436262021  0.4166666463745443  0.1569700000000012   F   F   F
  0.8750084390787186  0.3749450945460523  0.2386267359793159   T   T   T
  0.9166704760287457  0.4582885625565627  0.3223685035792473   T   T   T
  0.8749998492647180  0.5000000757544782  0.0000000000000000   F   F   F
  0.9166665639546849  0.5833335051344122  0.0784850000000006   F   F   F
  0.9583335778691477  0.5416667904444452  0.1569700000000012   F   F   F
  0.8750517844500681  0.5000303728996032  0.2386355075958893   T   T   T
  0.9167713232921944  0.5833291632498445  0.3224958068933951   T   T   T
  0.8749999835076636  0.6250002198243791  0.0000000000000000   F   F   F
  0.9166664479350644  0.7083331486791877  0.0784850000000006   F   F   F
  0.9583330283820857  0.6666664339892208  0.1569700000000012   F   F   F
  0.8750441774928925  0.6249802277573251  0.2386373694249301   T   T   T
  0.9167846205962739  0.7083495676668895  0.3224140148825805   T   T   T
  0.8749998674880501  0.7499998633691547  0.0000000000000000   F   F   F
  0.9166665821780171  0.8333332927490886  0.0784850000000006   F   F   F
  0.9583331626250313  0.7916665780591217  0.1569700000000012   F   F   F
  0.8750384824755867  0.7499965188553219  0.2386272115855010   T   T   T
  0.9167261617417881  0.8333375840403283  0.3223724586049072   T   T   T
  0.8750000017309958  0.8750000074390556  0.0000000000000000   F   F   F
  0.9166667164209628  0.9583334368189895  0.0784850000000006   F   F   F
  0.9583332968679841  0.9166667221290226  0.1569700000000012   F   F   F
  0.8750182944727136  0.8750275034557626  0.2385929264848662   T   T   T
  0.9166965290207343  0.9583415309981868  0.3224091208450008   T   T   T
  0.4981397374443788  0.4987444541542301  0.4246930632026708   T   T   T
  0.4968813220529382  0.6076642187741228  0.4258197791067935   T   T   T
  0.3879252849572198  0.4973768629432142  0.4253588701653682   T   T   T
  0.4631480242462623  0.1843243431841038  0.4257074342266295   T   T   T
  0.5723159653649948  0.2944262930679252  0.4251541764544037   T   T   T
  0.4622819694161499  0.2934446144133709  0.4247082831314418   T   T   T
  0.5317060434364665  0.5705330505613534  0.4252952447954559   T   T   T
  0.4263160618357206  0.4638516050405172  0.4248458913559977   T   T   T
  0.4250569155416224  0.5692419082517572  0.4261918340764884   T   T   T
  0.5349881854894891  0.2225632725998618  0.4258724963261889   T   T   T
  0.4285246728523127  0.2216471737090494  0.4253404663770769   T   T   T
  0.5341115872587792  0.3281452682298924  0.4247846394638051   T   T   T
  0.5288091360780905  0.4692259048700862  0.4243455084725177   T   T   T
  0.4013822734623937  0.4127990324571206  0.4238653398366077   T   T   T
  0.3278282607604190  0.4667651142037434  0.4252653115077730   T   T   T
  0.3990633556135978  0.5943025580860098  0.4262954034941833   T   T   T
  0.5264348915979777  0.6677542920134044  0.4260881345626407   T   T   T
  0.5827508530133764  0.5965495841940732  0.4245601282561782   T   T   T
  0.4334335617746649  0.1242360535708535  0.4260441247530231   T   T   T
  0.3774703106067069  0.1957732194706159  0.4247633453162271   T   T   T
  0.4317721075422573  0.3231328908854648  0.4243919669181742   T   T   T
  0.5591818101280515  0.3792072598565570  0.4238446268961418   T   T   T
  0.6324058166340905  0.3248778638518003  0.4250654001681876   T   T   T
  0.5608437957339083  0.1973625554101314  0.4258380484305509   T   T   T
```

Borazine dimer adsorbed on Au(111), dimer III

```
   23.0697829620000014    0.0000000000000000    0.0000000000000000
  -11.5348914810000007   19.9790169730000002    0.0000000000000000
    0.0000000000000000    0.0000000000000000   30.0000000000000000
  Au   B    N    H
```

```
  320     6     6    12
Selective dynamics
Direct
  0.0000000000000000  0.0000000000000000  0.0000000000000000   F   F   F
  0.0416667146899670  0.0833334293799339  0.0784850000000006   F   F   F
  0.0833332951369883  0.0416667146899670  0.1569700000000012   F   F   F
  0.0000233846025924  0.0000642879887868  0.2385558817829553   T   T   T
  0.0417300320156494  0.0834165798544141  0.3224134215432239   T   T   T
  0.0000001342429456  0.1250001440699009  0.0000000000000000   F   F   F
  0.0416668489329126  0.2083335734498348  0.0784850000000006   F   F   F
  0.0833334293799339  0.1666668587598679  0.1569700000000012   F   F   F
  0.0000150135670388  0.1250643605292474  0.2385952416486378   T   T   T
  0.0416649455323064  0.2083072856569265  0.3225654114919818   T   T   T
  0.0000000182233322  0.2499997876146764  0.0000000000000000   F   F   F
  0.0416667329132991  0.3333332169946104  0.0784850000000006   F   F   F
  0.0833333133603205  0.2916665023046434  0.1569700000000012   F   F   F
  0.0000538879464192  0.2500460820767986  0.2386442595630266   T   T   T
  0.0416953429372030  0.3333519609818880  0.3225849703976383   T   T   T
  0.0000001524662778  0.3749999316845773  0.0000000000000000   F   F   F
  0.0416668671562448  0.4583333610645113  0.0784850000000006   F   F   F
  0.0833334476032661  0.4166666463745443  0.1569700000000012   F   F   F
  0.0000550864278484  0.3749908507246652  0.2386687645739650   T   T   T
  0.0416478587307502  0.4583890359421265  0.3225353453872649   T   T   T
  0.9999997248487205  0.5000000757544782  0.0000000000000000   F   F   F
  0.0416665679317489  0.5833335051344122  0.0784850000000006   F   F   F
  0.0833335818462118  0.5416667904444452  0.1569700000000012   F   F   F
  0.0000053826752811  0.4999872734682401  0.2386635222500383   T   T   T
  0.0417028299644144  0.5833899535424677  0.3224237312614061   T   T   T
  0.9999998590916661  0.6250002198243791  0.0000000000000000   F   F   F
  0.0416664519121284  0.7083331486791877  0.0784850000000006   F   F   F
  0.0833330323591497  0.6666664339892208  0.1569700000000012   F   F   F
  0.0000105932138192  0.6250450810278344  0.2385907676963402   T   T   T
  0.0417360921604853  0.7084166093344140  0.3223996977509136   T   T   T
  0.9999997430720526  0.7499998633691547  0.0000000000000000   F   F   F
  0.0416665861550811  0.8333332927490886  0.0784850000000006   F   F   F
  0.0833331666020953  0.7916665780591217  0.1569700000000012   F   F   F
  0.0000099640301950  0.7500194949920723  0.2385623918961584   T   T   T
  0.0417225916507261  0.8334047155184847  0.3223491397313870   T   T   T
  0.0000000057080598  0.8750000074390556  0.0000000000000000   F   F   F
  0.0416667203980268  0.9583334368189895  0.0784850000000006   F   F   F
  0.0833333008450481  0.9166667221290226  0.1569700000000012   F   F   F
  0.0000238369059837  0.8750428765296332  0.2385546913277655   T   T   T
  0.0417324923432849  0.9584054975803984  0.3223203340567637   T   T   T
  0.1249998755840025  0.0000000000000000  0.0000000000000000   F   F   F
  0.1666665902739695  0.0833334293799339  0.0784850000000006   F   F   F
  0.2083331707209908  0.0416667146899670  0.1569700000000012   F   F   F
  0.1250961775849452  0.0000967272603280  0.2385630796819530   T   T   T
  0.1667769946250385  0.0834770519940713  0.3223033318052567   T   T   T
  0.1250000098269552  0.1250001440699009  0.0000000000000000   F   F   F
  0.1666667245169222  0.2083335734498348  0.0784850000000006   F   F   F
  0.2083333049639364  0.1666668587598679  0.1569700000000012   F   F   F
  0.1250334381457862  0.1250691839734687  0.2386287747223968   T   T   T
  0.1667260025949811  0.2084841862593496  0.3226010890499597   T   T   T
  0.1249998938073347  0.2499997876146764  0.0000000000000000   F   F   F
  0.1666666084973016  0.3333332169946104  0.0784850000000006   F   F   F
  0.2083331889443230  0.2916665023046434  0.1569700000000012   F   F   F
  0.1249222673226721  0.2499559674501279  0.2386484564152780   T   T   T
  0.1664556333982952  0.3332336087526890  0.3226792814893880   T   T   T
  0.1250000280502874  0.3749999316845773  0.0000000000000000   F   F   F
  0.1666667427402544  0.4583333610645113  0.0784850000000006   F   F   F
  0.2083333231872686  0.4166666463745443  0.1569700000000012   F   F   F
  0.1249715743901620  0.3749869484997365  0.2386634820173371   T   T   T
  0.1664532120802151  0.4582976832429033  0.3225932679665452   T   T   T
  0.1250001622932331  0.5000000757544782  0.0000000000000000   F   F   F
  0.1666668769832000  0.5833335051344122  0.0784850000000006   F   F   F
  0.2083334574302214  0.5416667904444452  0.1569700000000012   F   F   F
  0.1248981083898756  0.4999761248091802  0.2386183315549434   T   T   T
  0.1667068758812289  0.5833335420675498  0.3224893359289267   T   T   T
  0.1250002965361787  0.6250002198243791  0.0000000000000000   F   F   F
  0.1666667609635866  0.7083331486791877  0.0784850000000006   F   F   F
  0.2083333414106079  0.6666664339892208  0.1569700000000012   F   F   F
  0.1249534453323258  0.6249908684832621  0.2386005610915538   T   T   T
  0.1667413248116235  0.7083750063626919  0.3223061138578917   T   T   T
  0.1249997470491166  0.7499998633691547  0.0000000000000000   F   F   F
  0.1666664617390836  0.8333332927490886  0.0784850000000006   F   F   F
```

0.2083334756535535	0.7916665780591217	0.1569700000000012	F	F	F
0.1250308063353009	0.7500415374536528	0.2385595458915317	T	T	T
0.1667057136045217	0.8333906076377759	0.3223912196597256	T	T	T
0.1249998812920623	0.8750000074390556	0.0000000000000000	F	F	F
0.1666665959820293	0.9583334368189895	0.0784850000000006	F	F	F
0.2083331764290506	0.9166667221290226	0.1569700000000012	F	F	F
0.1250498750163951	0.8750633479391030	0.2385615947604466	T	T	T
0.1667247163143750	0.9584000426326696	0.3224125317086035	T	T	T
0.2500001846354607	0.0000000000000000	0.0000000000000000	F	F	F
0.2916668993254277	0.0833334293799339	0.0784850000000006	F	F	F
0.3333334797724490	0.0416667146899670	0.1569700000000012	F	F	F
0.2500626951838249	0.0000624199954261	0.2386198693760461	T	T	T
0.2917151876054451	0.0833726141176046	0.3226252674668892	T	T	T
0.2499998854109577	0.1250001440699009	0.0000000000000000	F	F	F
0.2916666001009247	0.2083335734498348	0.0784850000000006	F	F	F
0.3333336140153946	0.1666668587598679	0.1569700000000012	F	F	F
0.2501533197990778	0.1250799611266618	0.2387005588497034	T	T	T
0.2917783383321356	0.2085076974618344	0.3227143907386500	T	T	T
0.2499997693913443	0.2499997876146764	0.0000000000000000	F	F	F
0.2916664840813112	0.3333332169946104	0.0784850000000006	F	F	F
0.3333330645283255	0.2916665023046434	0.1569700000000012	F	F	F
0.2499106186871198	0.2498057076173291	0.2387175877320547	T	T	T
0.2916138131212990	0.3332827061300385	0.3225966405898361	T	T	T
0.2499999036342899	0.3749999316845773	0.0000000000000000	F	F	F
0.2916666183242569	0.4583333610645113	0.0784850000000006	F	F	F
0.3333331987712782	0.4166666463745443	0.1569700000000012	F	F	F
0.2497330572733095	0.3746138843487397	0.2385211339855970	T	T	T
0.2911880015950265	0.4581475752990062	0.3209768471151556	T	T	T
0.2500000378772356	0.5000000757544782	0.0000000000000000	F	F	F
0.2916667525672025	0.5833335051344122	0.0784850000000006	F	F	F
0.3333333330142239	0.5416667904444452	0.1569700000000012	F	F	F
0.2497585470118207	0.5001269893968963	0.2384596069399649	T	T	T
0.2916296898967538	0.5834892263286263	0.3223064498787586	T	T	T
0.2500001721201883	0.6250002198243791	0.0000000000000000	F	F	F
0.2916666365475891	0.7083331486791877	0.0784850000000006	F	F	F
0.3333332169946104	0.6666664339892208	0.1569700000000012	F	F	F
0.2498852000698277	0.6251612476309116	0.2386142088834952	T	T	T
0.2917784981569413	0.7084125217423611	0.3224803126280144	T	T	T
0.2500000561005749	0.7499998633691547	0.0000000000000000	F	F	F
0.2916667707905418	0.8333332927490886	0.0784850000000006	F	F	F
0.3333333512375560	0.7916665780591217	0.1569700000000012	F	F	F
0.2500636566583245	0.7501117683484234	0.2386013606417360	T	T	T
0.2917175620083504	0.8334244341784436	0.3224192153050907	T	T	T
0.2500001903435205	0.8750000074390556	0.0000000000000000	F	F	F
0.2916669050334875	0.9583334368189895	0.0784850000000006	F	F	F
0.3333334854805088	0.9166667221290226	0.1569700000000012	F	F	F
0.2500316306099796	0.8750767516647528	0.2385830270649606	T	T	T
0.2916422968151040	0.9583706821636939	0.3225529562827286	T	T	T
0.3750000602194632	0.0000000000000000	0.0000000000000000	F	F	F
0.4166667749094302	0.0833334293799339	0.0784850000000006	F	F	F
0.4583333553564515	0.0416667146899670	0.1569700000000012	F	F	F
0.3749133889799197	0.9999059262188816	0.2386560796522110	T	T	T
0.4165704002393165	0.0832204379033466	0.3226778590915982	T	T	T
0.3750001944624159	0.1250001440699009	0.0000000000000000	F	F	F
0.4166669091523829	0.2083335734498348	0.0784850000000006	F	F	F
0.4583334895993971	0.1666668587598679	0.1569700000000012	F	F	F
0.3749641945940974	0.1249375603018947	0.2387055017568931	T	T	T
0.4164815069669426	0.2082196941612656	0.3226144884716660	T	T	T
0.3750000784427954	0.2499997876146764	0.0000000000000000	F	F	F
0.4166667931327623	0.3333332169946104	0.0784850000000006	F	F	F
0.4583333735797837	0.2916665023046434	0.1569700000000012	F	F	F
0.3749750729856646	0.2499889665097801	0.2387619122318374	T	T	T
0.4166870044788240	0.3333030522027727	0.3229209427121424	T	T	T
0.3749997792182995	0.3749999316845773	0.0000000000000000	F	F	F
0.4166664939082665	0.4583333610645113	0.0784850000000006	F	F	F
0.4583335078227293	0.4166666463745443	0.1569700000000012	F	F	F
0.3747593367096564	0.3746522497737458	0.2385623600697536	T	T	T
0.4169857438066449	0.4581530955833156	0.3210191178635848	T	T	T
0.3749999134612452	0.5000000757544782	0.0000000000000000	F	F	F
0.4166666281512121	0.5833335051344122	0.0784850000000006	F	F	F
0.4583332085982335	0.5416667904444452	0.1569700000000012	F	F	F
0.3750090994151430	0.5000163757243055	0.2384415641435852	T	T	T
0.4169907028751148	0.5839708455410801	0.3207945019592068	T	T	T
0.3750000477041908	0.6250002198243791	0.0000000000000000	F	F	F
0.4166665121315987	0.7083331486791877	0.0784850000000006	F	F	F

0.4583330925786129	0.6666664339892208	0.1569700000000012	F	F	F
0.3749110026384909	0.6253193412346694	0.2384262073709024	T	T	T
0.4168226693627944	0.7086956353535112	0.3225633591713734	T	T	T
0.3749999316845773	0.7499998633691547	0.0000000000000000	F	F	F
0.4166666463745443	0.8333332927490886	0.0784850000000006	F	F	F
0.4583332268215656	0.7916665780591217	0.1569700000000012	F	F	F
0.3750713465445444	0.7501800467539258	0.2386131912210802	T	T	T
0.4167133611792885	0.8334837208474597	0.3225340989237542	T	T	T
0.3750000659275230	0.8750000074390556	0.0000000000000000	F	F	F
0.4166667806174900	0.9583334368189895	0.0784850000000006	F	F	F
0.4583333610645113	0.9166667221290226	0.1569700000000012	F	F	F
0.3750703998803589	0.8750743270552325	0.2386545759688683	T	T	T
0.4167198433242056	0.9584630726498414	0.3225636426372507	T	T	T
0.4999999358034728	0.0000000000000000	0.0000000000000000	F	F	F
0.5416666504934398	0.0833334293799339	0.0784850000000006	F	F	F
0.5833332309404540	0.0416667146899670	0.1569700000000012	F	F	F
0.5000028907970417	0.9998751701942211	0.2386412283771119	T	T	T
0.5416774248711224	0.0832265375823280	0.3226038785250072	T	T	T
0.5000000700464184	0.1250001440699009	0.0000000000000000	F	F	F
0.5416667847363854	0.2083335734498348	0.0784850000000006	F	F	F
0.5833333651834067	0.1666668587598679	0.1569700000000012	F	F	F
0.4997561069162016	0.1245163434831415	0.2384625183618310	T	T	T
0.5414866054403997	0.2079090984408539	0.3210197760231863	T	T	T
0.4999999540268050	0.2499997876146764	0.0000000000000000	F	F	F
0.5416666687167719	0.3333332169946104	0.0784850000000006	F	F	F
0.5833332491637861	0.2916665023046434	0.1569700000000012	F	F	F
0.4997766863942701	0.2499166009461697	0.2384756656778601	T	T	T
0.5415257997524572	0.3336141789709990	0.3209534292073297	T	T	T
0.5000000882697506	0.3749999316845773	0.0000000000000000	F	F	F
0.5416668029597176	0.4583333610645113	0.0784850000000006	F	F	F
0.5833333834067389	0.4166666463745443	0.1569700000000012	F	F	F
0.4997746301012423	0.3752808398873630	0.2385313341635055	T	T	T
0.5418310884658060	0.4584387023654262	0.3229182272052225	T	T	T
0.5000002225126963	0.5000000757544782	0.0000000000000000	F	F	F
0.5416669372026632	0.5833335051344122	0.0784850000000006	F	F	F
0.5833335176496846	0.5416667904444452	0.1569700000000012	F	F	F
0.5003876340817768	0.5002883214027111	0.2385466687560416	T	T	T
0.5419003444124501	0.5835445484812943	0.3224853693475074	T	T	T
0.4999999232882004	0.6250002198243791	0.0000000000000000	F	F	F
0.5416663877156012	0.7083331486791877	0.0784850000000006	F	F	F
0.5833334016300711	0.6666664339892208	0.1569700000000012	F	F	F
0.5004699631856934	0.6253469770693467	0.2384747002606606	T	T	T
0.5419108959476712	0.7087124160427060	0.3226236234368873	T	T	T
0.4999998072685798	0.7499998633691547	0.0000000000000000	F	F	F
0.5416665219585468	0.8333332927490886	0.0784850000000006	F	F	F
0.5833331024055681	0.7916665780591217	0.1569700000000012	F	F	F
0.5000730400557319	0.7501042690574470	0.2386440399135485	T	T	T
0.5417512836278329	0.8334339195271334	0.3225814964656637	T	T	T
0.4999999415115326	0.8750000074390556	0.0000000000000000	F	F	F
0.5416666562014996	0.9583334368189895	0.0784850000000006	F	F	F
0.5833332366485138	0.9166667221290226	0.1569700000000012	F	F	F
0.5000606097010258	0.8750119432175464	0.2386650744876862	T	T	T
0.5417376712366540	0.9583846206325884	0.3225381376784260	T	T	T
0.6249998113874753	0.0000000000000000	0.0000000000000000	F	F	F
0.6666665260774423	0.0833334293799339	0.0784850000000006	F	F	F
0.7083335399919122	0.0416667146899670	0.1569700000000012	F	F	F
0.6250071015504314	0.9999830204724355	0.2386240156645407	T	T	T
0.6666903685677387	0.0833394200119989	0.3225368295400738	T	T	T
0.6249999456304209	0.1250001440699009	0.0000000000000000	F	F	F
0.6666666603203879	0.2083335734498348	0.0784850000000006	F	F	F
0.7083332407674092	0.1666668587598679	0.1569700000000012	F	F	F
0.6249921938536130	0.1248957086099775	0.2386703446335783	T	T	T
0.6668514072330136	0.2082557129427512	0.3224025304108937	T	T	T
0.6249998296108075	0.2499997876146764	0.0000000000000000	F	F	F
0.6666665443007744	0.3333332169946104	0.0784850000000006	F	F	F
0.7083331247477958	0.2916665023046434	0.1569700000000012	F	F	F
0.6251049058138300	0.2499013922755771	0.2383997948937159	T	T	T
0.6672670608328263	0.3336324540418050	0.3208053901678841	T	T	T
0.6249999638537531	0.3749999316845773	0.0000000000000000	F	F	F
0.6666666785437201	0.4583333610645113	0.0784850000000006	F	F	F
0.7083332589907414	0.4166666463745443	0.1569700000000012	F	F	F
0.6251389689346324	0.3752897449842462	0.2384348168536839	T	T	T
0.6669562556884270	0.4586916298924879	0.3224837905584722	T	T	T
0.6250000980967059	0.5000000757544782	0.0000000000000000	F	F	F
0.6666668127866728	0.5833335051344122	0.0784850000000006	F	F	F

0.7083333932336870	0.5416667904444452	0.1569700000000012	F	F	F
0.6250648661159409	0.5001030378694420	0.2387390537453692	T	T	T
0.6666308068796766	0.5833429744381756	0.3227315992902228	T	T	T
0.6250002323396515	0.6250002198243791	0.0000000000000000	F	F	F
0.6666666967670594	0.7083331486791877	0.0784850000000006	F	F	F
0.7083332772140736	0.6666664339892208	0.1569700000000012	F	F	F
0.6252967772412825	0.6251703598040488	0.2386983449297204	T	T	T
0.6666537896795089	0.7084045756287510	0.3225999709162911	T	T	T
0.6250001163200380	0.7499998633691547	0.0000000000000000	F	F	F
0.6666668310100050	0.8333332927490886	0.0784850000000006	F	F	F
0.7083334114570263	0.7916665780591217	0.1569700000000012	F	F	F
0.6251346315264589	0.7501565258390901	0.2386365125464980	T	T	T
0.6668116048974074	0.8334593575928644	0.3225662375130540	T	T	T
0.6249998170955351	0.8750000074390556	0.0000000000000000	F	F	F
0.6666665317855021	0.9583334368189895	0.0784850000000006	F	F	F
0.7083335456999720	0.9166667221290226	0.1569700000000012	F	F	F
0.6250112129276139	0.8750025114949977	0.2386462531774441	T	T	T
0.6667363265745353	0.9583878349769728	0.3224438894306710	T	T	T
0.7500001204389335	0.0000000000000000	0.0000000000000000	F	F	F
0.7916668351289005	0.0833334293799339	0.0784850000000006	F	F	F
0.8333334155759147	0.0416667146899670	0.1569700000000012	F	F	F
0.7499935798253996	0.0000419776742111	0.2385729269472998	T	T	T
0.7917191529397758	0.0833855993392103	0.3223456655885289	T	T	T
0.7500002546818791	0.1250001440699009	0.0000000000000000	F	F	F
0.7916669693718461	0.2083335734498348	0.0784850000000006	F	F	F
0.8333335498188674	0.1666668587598679	0.1569700000000012	F	F	F
0.7499946995777712	0.1250589547454112	0.2386438135150749	T	T	T
0.7917633126132859	0.2084012767858146	0.3225396693909588	T	T	T
0.7499997051948100	0.2499997876146764	0.0000000000000000	F	F	F
0.7916664198847769	0.3333332169946104	0.0784850000000006	F	F	F
0.8333334337992468	0.2916665023046434	0.1569700000000012	F	F	F
0.7501532233629100	0.2500549792177713	0.2386719082116232	T	T	T
0.7919367484723426	0.3334261049907816	0.3225896962876036	T	T	T
0.7499998394377627	0.3749999316845773	0.0000000000000000	F	F	F
0.7916665541277297	0.4583333610645113	0.0784850000000006	F	F	F
0.8333331345747439	0.4166666463745443	0.1569700000000012	F	F	F
0.7505880514022014	0.3753057786037549	0.2384135951306031	T	T	T
0.7919669042324331	0.4585658359903831	0.3226068533920834	T	T	T
0.7499999736807084	0.5000000757544782	0.0000000000000000	F	F	F
0.7916666883706753	0.5833335051344122	0.0784850000000006	F	F	F
0.8333332688176966	0.5416667904444452	0.1569700000000012	F	F	F
0.7501646659028725	0.5001168607776745	0.2386804482952248	T	T	T
0.7917798244302726	0.5834151521671916	0.3226208676055685	T	T	T
0.7500001079236540	0.6250002198243791	0.0000000000000000	F	F	F
0.7916665723510619	0.7083331486791877	0.0784850000000006	F	F	F
0.8333331527980832	0.6666664339892208	0.1569700000000012	F	F	F
0.7499971307356572	0.6249104857422200	0.2387079497413399	T	T	T
0.7916522157293333	0.7083403845585337	0.3223120819358967	T	T	T
0.7499999919040405	0.7499998633691547	0.0000000000000000	F	F	F
0.7916667065940075	0.8333332927490886	0.0784850000000006	F	F	F
0.8333332870410288	0.7916665780591217	0.1569700000000012	F	F	F
0.7500157452371156	0.7500378002477069	0.2386340335182096	T	T	T
0.7917045073346969	0.8333856987807281	0.3224197694028965	T	T	T
0.7500001261469933	0.8750000074390556	0.0000000000000000	F	F	F
0.7916668408369603	0.9583334368189895	0.0784850000000006	F	F	F
0.8333334212839745	0.9166667221290226	0.1569700000000012	F	F	F
0.7500073176492201	0.8750543082891505	0.2386006087519116	T	T	T
0.7917436875659342	0.9583982539380873	0.3224094545896591	T	T	T
0.8749999960229360	0.0000000000000000	0.0000000000000000	F	F	F
0.9166667107129030	0.0833334293799339	0.0784850000000006	F	F	F
0.9583332911599243	0.0416667146899670	0.1569700000000012	F	F	F
0.8750155262793050	0.0000473941815378	0.2385744254945479	T	T	T
0.9167155127123474	0.0833697067755699	0.3224036848938485	T	T	T
0.8750001302658816	0.1250001440699009	0.0000000000000000	F	F	F
0.9166668449558486	0.2083335734498348	0.0784850000000006	F	F	F
0.9583334254028699	0.1666668587598679	0.1569700000000012	F	F	F
0.8750131804513500	0.1250622864507105	0.2385754935325274	T	T	T
0.9167258234050296	0.2083697769612559	0.3224462497277688	T	T	T
0.8750000142462682	0.2499997876146764	0.0000000000000000	F	F	F
0.9166667289362351	0.3333332169946104	0.0784850000000006	F	F	F
0.9583333093832564	0.2916665023046434	0.1569700000000012	F	F	F
0.8750697280111832	0.2500468979927807	0.2386284211267382	T	T	T
0.9167467980002026	0.3333608285789393	0.3225570719579852	T	T	T
0.8750001484892138	0.3749999316845773	0.0000000000000000	F	F	F
0.9166668631791808	0.4583333610645113	0.0784850000000006	F	F	F

```
0.9583334436262021  0.4166666463745443  0.1569700000000012   F   F   F
0.8752122677461813  0.3750546421836917  0.2386386019699980   T   T   T
0.9166717801712622  0.4583778553955121  0.3225887203283653   T   T   T
0.8749998492647180  0.5000000757544782  0.0000000000000000   F   F   F
0.9166665639546849  0.5833335051344122  0.0784850000000006   F   F   F
0.9583335778691477  0.5416667904444452  0.1569700000000012   F   F   F
0.8752134939490609  0.5001666443695554  0.2386429598413691   T   T   T
0.9167732754713497  0.5834737910203950  0.3225619439065739   T   T   T
0.8749999835076636  0.6250002198243791  0.0000000000000000   F   F   F
0.9166664479350644  0.7083331486791877  0.0784850000000006   F   F   F
0.9583330283820857  0.6666664339892208  0.1569700000000012   F   F   F
0.8750252356849262  0.6250080524586127  0.2386220516913787   T   T   T
0.9167340939742914  0.7083963080124558  0.3224179395096738   T   T   T
0.8749998674880501  0.7499998633691547  0.0000000000000000   F   F   F
0.9166665821780171  0.8333332927490886  0.0784850000000006   F   F   F
0.9583331626250313  0.7916665780591217  0.1569700000000012   F   F   F
0.8749740664801604  0.7499631539913997  0.2385708411323831   T   T   T
0.9167224577399327  0.8333906583533940  0.3223177048380304   T   T   T
0.8750000017309958  0.8750000074390556  0.0000000000000000   F   F   F
0.9166667164209628  0.9583334368189895  0.0784850000000006   F   F   F
0.9583332968679841  0.9166667221290226  0.1569700000000012   F   F   F
0.8750039918027634  0.8750409888948233  0.2385562300716783   T   T   T
0.9167323321438103  0.9583811899545005  0.3223751147385109   T   T   T
0.4492711382364695  0.5394651107700092  0.4245106749105436   T   T   T
0.3397956356859002  0.4295499620609632  0.4250036663639194   T   T   T
0.3393751200602123  0.5389667690673039  0.4246611673662203   T   T   T
0.6215499437043722  0.2563441615207074  0.4249386351089645   T   T   T
0.5128398459581281  0.2581547507136681  0.4250975064396145   T   T   T
0.6234397051382428  0.3669197413517534  0.4245359914742118   T   T   T
0.4116868113909720  0.4675744629510270  0.4249100802454399   T   T   T
0.4112892300370236  0.5733492682030432  0.4245614740040402   T   T   T
0.3054762982737557  0.4670947789011689  0.4250792941901037   T   T   T
0.5497105122316090  0.2231435263098363  0.4253599818137148   T   T   T
0.6566279308899242  0.3282803447343710  0.4247361373740564   T   T   T
0.5515316983789059  0.3300107279183814  0.4249027716830985   T   T   T
0.4373670006779989  0.4422256178640795  0.4242325382513469   T   T   T
0.3099978826547660  0.3694487850967790  0.4251950189122329   T   T   T
0.2544157599188316  0.4414262127271156  0.4247995127194857   T   T   T
0.3091519452909492  0.5688500709160549  0.4245947147891681   T   T   T
0.4366471071773955  0.6244074272300556  0.4238650203318731   T   T   T
0.5093928598795051  0.5696705036161362  0.4243378480248125   T   T   T
0.5235584659640423  0.1720873919211168  0.4251492408895550   T   T   T
0.4527472842331418  0.2289337035450921  0.4251951173152845   T   T   T
0.5266860010476523  0.3561836035819101  0.4240536018097251   T   T   T
0.6542008520354317  0.4270284710679505  0.4242482052345268   T   T   T
0.7076778447280407  0.3531373635215415  0.4240456179625954   T   T   T
0.6508604701306256  0.2255521873380209  0.4249314665177792   T   T   T
```

Borazine adsorbed on Au(111), hpc, with 9 H2 molecules

```
   23.0697829620000014    0.0000000000000000    0.0000000000000000
  -11.5348914810000007   19.9790169730000002    0.0000000000000000
    0.0000000000000000    0.0000000000000000   30.0000000000000000
  Au   B    N    H
  320    3    3   24
Selective dynamics
Direct
0.0000000000000000  0.0000000000000000  0.0000000000000000   F   F   F
0.0416667146899670  0.0833334293799339  0.0784850000000006   F   F   F
0.0833332951369883  0.0416667146899670  0.1569700000000012   F   F   F
0.9999942302177672  0.9999724617849010  0.2385942084569199   T   T   T
0.0416444834701767  0.0832707018954374  0.3224201531346439   T   T   T
0.0000001342429456  0.1250001440699009  0.0000000000000000   F   F   F
0.0416668489329126  0.2083335734498348  0.0784850000000006   F   F   F
0.0833334293799339  0.1666668587598679  0.1569700000000012   F   F   F
0.0000326268387682  0.1249982851864430  0.2385918248734053   T   T   T
0.0416510909569179  0.2083067368088353  0.3224241695271849   T   T   T
0.0000000182233322  0.2499997876146764  0.0000000000000000   F   F   F
0.0416667329132991  0.3333332169946104  0.0784850000000006   F   F   F
0.0833333133603205  0.2916665023046434  0.1569700000000012   F   F   F
0.9999979768646937  0.2499856038650661  0.2385958654265448   T   T   T
0.0416357179242383  0.3333084787886944  0.3224785802952575   T   T   T
0.0000001524662778  0.3749999316845773  0.0000000000000000   F   F   F
0.0416668671562448  0.4583333610645113  0.0784850000000006   F   F   F
0.0833334476032661  0.4166666463745443  0.1569700000000012   F   F   F
0.9999771524925763  0.3749570962745201  0.2386076549849698   T   T   T
```

0.0416533548587948	0.4583065439634854	0.3224294371264849	T	T	T
0.9999997248487205	0.5000000757544782	0.0000000000000000	F	F	F
0.0416665679317489	0.5833335051344122	0.0784850000000006	F	F	F
0.0833335818462118	0.5416667904444452	0.1569700000000012	F	F	F
0.9999703164252420	0.4999369171881610	0.2385844326156740	T	T	T
0.0416128720399919	0.5832666882028421	0.3223689208740145	T	T	T
0.9999998590916661	0.6250002198243791	0.0000000000000000	F	F	F
0.0416664519121284	0.7083331486791877	0.0784850000000006	F	F	F
0.0833330323591497	0.6666664339892208	0.1569700000000012	F	F	F
0.9999539219449908	0.6249576880772950	0.2385819410663911	T	T	T
0.0416050451720956	0.7082636922354987	0.3223739750143809	T	T	T
0.9999997430720526	0.7499998633691547	0.0000000000000000	F	F	F
0.0416665861550811	0.8333332927490886	0.0784850000000006	F	F	F
0.0833331666020953	0.7916665780591217	0.1569700000000012	F	F	F
0.9999603088145934	0.7499854158288942	0.2385878736996161	T	T	T
0.0416335737129728	0.8332584531107330	0.3224307604245747	T	T	T
0.0000000057080598	0.8750000074390556	0.0000000000000000	F	F	F
0.0416667203980268	0.9583334368189895	0.0784850000000006	F	F	F
0.0833333008450481	0.9166667221290226	0.1569700000000012	F	F	F
0.9999673519292822	0.8749739405414388	0.2386087245544236	T	T	T
0.0416156767591685	0.9582382395783586	0.3224802256242272	T	T	T
0.1249998755840025	0.0000000000000000	0.0000000000000000	F	F	F
0.1666665902739695	0.0833334293799339	0.0784850000000006	F	F	F
0.2083331707209908	0.0416667146899670	0.1569700000000012	F	F	F
0.1249366624834979	0.9999536123444367	0.2386225835835539	T	T	T
0.1665567725715658	0.0831810761946515	0.3225241382071096	T	T	T
0.1250000098269552	0.1250001440699009	0.0000000000000000	F	F	F
0.1666667245169222	0.2083335734498348	0.0784850000000006	F	F	F
0.2083333049639364	0.1666668587598679	0.1569700000000012	F	F	F
0.1250023405920763	0.1249784010265858	0.2386594319601923	T	T	T
0.1665903080460419	0.2082557283458802	0.3225170043405993	T	T	T
0.1249998938073347	0.2499997876146764	0.0000000000000000	F	F	F
0.1666666084973016	0.3333332169946104	0.0784850000000006	F	F	F
0.2083331889443230	0.2916665023046434	0.1569700000000012	F	F	F
0.1250027538079080	0.2499865656634011	0.2386587676364375	T	T	T
0.1665762586689002	0.3332981981558484	0.3225256687637407	T	T	T
0.1250000280502874	0.3749999316845773	0.0000000000000000	F	F	F
0.1666667427402544	0.4583333610645113	0.0784850000000006	F	F	F
0.2083333231872686	0.4166666463745443	0.1569700000000012	F	F	F
0.1249469043430942	0.3749449848161339	0.2386196851022682	T	T	T
0.1666669095516098	0.4582704235893340	0.3224563310266835	T	T	T
0.1250001622932331	0.5000000757544782	0.0000000000000000	F	F	F
0.1666668769832000	0.5833335051344122	0.0784850000000006	F	F	F
0.2083334574302214	0.5416667904444452	0.1569700000000012	F	F	F
0.1249664634408768	0.4999374264284942	0.2385845055692479	T	T	T
0.1666605427666205	0.5832678359536918	0.3223689427917801	T	T	T
0.1250002965361787	0.6250002198243791	0.0000000000000000	F	F	F
0.1666667609635866	0.7083331486791877	0.0784850000000006	F	F	F
0.2083333414106079	0.6666664339892208	0.1569700000000012	F	F	F
0.1249757411259503	0.6249495408715370	0.2385778885008751	T	T	T
0.1666451589940665	0.7082787842877329	0.3223605955885581	T	T	T
0.1249997470491166	0.7499998633691547	0.0000000000000000	F	F	F
0.1666664617390836	0.8333332927490886	0.0784850000000006	F	F	F
0.2083334756535535	0.7916665780591217	0.1569700000000012	F	F	F
0.1249704760604779	0.7499749932382234	0.2385782651520504	T	T	T
0.1666469688348958	0.8332944724501631	0.3223748392585145	T	T	T
0.1249998812920623	0.8750000074390556	0.0000000000000000	F	F	F
0.1666665959820293	0.9583334368189895	0.0784850000000006	F	F	F
0.2083331764290506	0.9166667221290226	0.1569700000000012	F	F	F
0.1249541578369890	0.8749747561190628	0.2385863315664711	T	T	T
0.1666416499305886	0.9582910294254274	0.3224617189854372	T	T	T
0.2500001846354607	0.0000000000000000	0.0000000000000000	F	F	F
0.2916668993254277	0.0833334293799339	0.0784850000000006	F	F	F
0.3333334797724490	0.0416667146899670	0.1569700000000012	F	F	F
0.2499168714502738	0.9999252162794009	0.2386485797853139	T	T	T
0.2916214596853687	0.0833192580340088	0.3225153518476861	T	T	T
0.2499998854109577	0.1250001440699009	0.0000000000000000	F	F	F
0.2916666001009247	0.2083335734498348	0.0784850000000006	F	F	F
0.3333336140153946	0.1666668587598679	0.1569700000000012	F	F	F
0.2498409344730624	0.1248263874523241	0.2386393947569423	T	T	T
0.2912920250437942	0.2080360814871728	0.3224614441605618	T	T	T
0.2499997693913443	0.2499997876146764	0.0000000000000000	F	F	F
0.2916664840813112	0.3333332169946104	0.0784850000000006	F	F	F
0.3333330645283255	0.2916665023046434	0.1569700000000012	F	F	F
0.2498934738472723	0.2499248608862586	0.2386399842477608	T	T	T

0.2913172281825443	0.3331597315548751	0.3224761202650476	T	T	T
0.2499999036342899	0.3749999316845773	0.0000000000000000	F	F	F
0.2916666183242569	0.4583333610645113	0.0784850000000006	F	F	F
0.3333331987712782	0.4166666463745443	0.1569700000000012	F	F	F
0.2498511081494417	0.3749657065152144	0.2386419485797786	T	T	T
0.2916589970841965	0.4582044699647291	0.3225023820550593	T	T	T
0.2500000378772356	0.5000000757544782	0.0000000000000000	F	F	F
0.2916667525672025	0.5833335051344122	0.0784850000000006	F	F	F
0.3333333330142239	0.5416667904444452	0.1569700000000012	F	F	F
0.2499383298177509	0.4999421851155094	0.2386446122335407	T	T	T
0.2917075800782138	0.5832249051588689	0.3223737322184487	T	T	T
0.2500001721201883	0.6250002198243791	0.0000000000000000	F	F	F
0.2916666365475891	0.7083331486791877	0.0784850000000006	F	F	F
0.3333332169946104	0.6666664339892208	0.1569700000000012	F	F	F
0.2500064468777157	0.6249601299194710	0.2385826290555687	T	T	T
0.2916711977983725	0.7082680357203093	0.3223740805616926	T	T	T
0.2500000561005749	0.7499998633691547	0.0000000000000000	F	F	F
0.2916667707905418	0.8333332927490886	0.0784850000000006	F	F	F
0.3333333512375560	0.7916665780591217	0.1569700000000012	F	F	F
0.2500111822675323	0.7499776596387505	0.2385783502348816	T	T	T
0.2916650305978542	0.8332983469092584	0.3223741275824366	T	T	T
0.2500001903435205	0.8750000074390556	0.0000000000000000	F	F	F
0.2916669050334875	0.9583334368189895	0.0784850000000006	F	F	F
0.3333334854805088	0.9166667221290226	0.1569700000000012	F	F	F
0.2499949046104582	0.8749829723728570	0.2385855246008750	T	T	T
0.2916914026075539	0.9583700933155473	0.3223828495678108	T	T	T
0.3750000602194632	0.0000000000000000	0.0000000000000000	F	F	F
0.4166667749094302	0.0833334293799339	0.0784850000000006	F	F	F
0.4583333553564515	0.0416667146899670	0.1569700000000012	F	F	F
0.3750150108778031	0.9999252709778907	0.2386492654081604	T	T	T
0.4167069594748380	0.0833219493702854	0.3225125425194896	T	T	T
0.3750001944624159	0.1250001440699009	0.0000000000000000	F	F	F
0.4166669091523829	0.2083335734498348	0.0784850000000006	F	F	F
0.4583334895993971	0.1666668587598679	0.1569700000000012	F	F	F
0.3748358930673474	0.1246695330062923	0.2386560784600076	T	T	T
0.4165283013121320	0.2080613463126652	0.3222824527180740	T	T	T
0.3750000784427954	0.2499997876146764	0.0000000000000000	F	F	F
0.4166667931327623	0.3333332169946104	0.0784850000000006	F	F	F
0.4583333735797837	0.2916665023046434	0.1569700000000012	F	F	F
0.3747118479033452	0.2495763317190479	0.2384684240470875	T	T	T
0.4160076007740879	0.3329287677816714	0.3208987562924485	T	T	T
0.3749997792182995	0.3749999316845773	0.0000000000000000	F	F	F
0.4166664939082665	0.4583333610645113	0.0784850000000006	F	F	F
0.4583335078227293	0.4166666463745443	0.1569700000000012	F	F	F
0.3747173047097178	0.3750896168902048	0.2384839314390972	T	T	T
0.4165672471584847	0.4583042691042818	0.3223904787654756	T	T	T
0.3749999134612452	0.5000000757544782	0.0000000000000000	F	F	F
0.4166666281512121	0.5833335051344122	0.0784850000000006	F	F	F
0.4583332085982335	0.5416667904444452	0.1569700000000012	F	F	F
0.3748703413791432	0.5000965026672032	0.2386760476381708	T	T	T
0.4167176547011793	0.5832466964661117	0.3225162650625525	T	T	T
0.3750000477041908	0.6250002198243791	0.0000000000000000	F	F	F
0.4166665121315987	0.7083331486791877	0.0784850000000006	F	F	F
0.4583330925786129	0.6666664339892208	0.1569700000000012	F	F	F
0.3750323014717426	0.6250226573171361	0.2386475565958011	T	T	T
0.4166698330747114	0.7082479518155091	0.3224664020964559	T	T	T
0.3749999316845773	0.7499998633691547	0.0000000000000000	F	F	F
0.4166666463745443	0.8333332927490886	0.0784850000000006	F	F	F
0.4583332268215656	0.7916665780591217	0.1569700000000012	F	F	F
0.3750319574745685	0.7499873946748683	0.2385877474459568	T	T	T
0.4166409426218028	0.8332618518380216	0.3224316576963124	T	T	T
0.3750000659275230	0.8750000074390556	0.0000000000000000	F	F	F
0.4166667806174900	0.9583334368189895	0.0784850000000006	F	F	F
0.4583333610645113	0.9166667221290226	0.1569700000000012	F	F	F
0.3750295031345720	0.8749762909490034	0.2385848896174522	T	T	T
0.4166642264764817	0.9582928300443581	0.3224594336609088	T	T	T
0.4999999358034728	0.0000000000000000	0.0000000000000000	F	F	F
0.5416666504934398	0.0833334293799339	0.0784850000000006	F	F	F
0.5833332309404540	0.0416667146899670	0.1569700000000012	F	F	F
0.5000244978281518	0.9999554823539082	0.2386214084391796	T	T	T
0.5416333335196425	0.0831834899533981	0.3225218380133762	T	T	T
0.5000000700464184	0.1250001440699009	0.0000000000000000	F	F	F
0.5416667847363854	0.2083335734498348	0.0784850000000006	F	F	F
0.5833333651834067	0.1666668587598679	0.1569700000000012	F	F	F
0.4999896099595512	0.1248264979628692	0.2386378819170195	T	T	T

0.5417437350038684	0.2080365985545268	0.3224579525154537	T	T	T
0.4999999540268050	0.2499997876146764	0.0000000000000000	F	F	F
0.5416666687167719	0.3333332169946104	0.0784850000000006	F	F	F
0.5833332491637861	0.2916665023046434	0.1569700000000012	F	F	F
0.4998619862628692	0.2495729288948110	0.2384635299137084	T	T	T
0.5419090778883399	0.3329242201997994	0.3208834387714964	T	T	T
0.5000000882697506	0.3749999316845773	0.0000000000000000	F	F	F
0.5416668029597176	0.4583333610645113	0.0784850000000006	F	F	F
0.5833333834067389	0.4166666463745443	0.1569700000000012	F	F	F
0.4999834520711002	0.3749719691365145	0.2384624984134112	T	T	T
0.5418915248230961	0.4587862619486957	0.3210542777257282	T	T	T
0.5000002225126963	0.5000000757544782	0.0000000000000000	F	F	F
0.5416669372026632	0.5833335051344122	0.0784850000000006	F	F	F
0.5833335176496846	0.5416667904444452	0.1569700000000012	F	F	F
0.4998713888585039	0.5002081838640454	0.2385141712732360	T	T	T
0.5417258608682469	0.5835221116818020	0.3225150960994717	T	T	T
0.4999999232882004	0.6250002198243791	0.0000000000000000	F	F	F
0.5416663877156012	0.7083331486791877	0.0784850000000006	F	F	F
0.5833334016300711	0.6666664339892208	0.1569700000000012	F	F	F
0.5000033131019161	0.6250840264317858	0.2386506097776572	T	T	T
0.5416287616124195	0.7083125474568103	0.3225226306495490	T	T	T
0.4999998072685798	0.7499998633691547	0.0000000000000000	F	F	F
0.5416665219585468	0.8333332927490886	0.0784850000000006	F	F	F
0.5833331024055681	0.7916665780591217	0.1569700000000012	F	F	F
0.5000242568392567	0.7500018926468701	0.2386208911088056	T	T	T
0.5416351502969144	0.8332734869756779	0.3224744745373489	T	T	T
0.4999999415115326	0.8750000074390556	0.0000000000000000	F	F	F
0.5416666562014996	0.9583334368189895	0.0784850000000006	F	F	F
0.5833332366485138	0.9166667221290226	0.1569700000000012	F	F	F
0.5000149544402014	0.8749768348063470	0.2386082060214933	T	T	T
0.5416371983904510	0.9582420418914853	0.3224797307708313	T	T	T
0.6249998113874753	0.0000000000000000	0.0000000000000000	F	F	F
0.6666665260774423	0.0833334293799339	0.0784850000000006	F	F	F
0.7083335399919122	0.0416667146899670	0.1569700000000012	F	F	F
0.6249863230633497	0.9999740952305660	0.2385946621299596	T	T	T
0.6666352271299140	0.0832722548243421	0.3224183021865283	T	T	T
0.6249999456304209	0.1250001440699009	0.0000000000000000	F	F	F
0.6666666603203879	0.2083335734498348	0.0784850000000006	F	F	F
0.7083332407674092	0.1666668587598679	0.1569700000000012	F	F	F
0.6249804243383386	0.1249786550475274	0.2386579822930200	T	T	T
0.6666685491437438	0.2082567346732538	0.3225150445904166	T	T	T
0.6249998296108075	0.2499997876146764	0.0000000000000000	F	F	F
0.6666665443007744	0.3333332169946104	0.0784850000000006	F	F	F
0.7083331247477958	0.2916665023046434	0.1569700000000012	F	F	F
0.6250322636018395	0.2499230367473359	0.2386390503428351	T	T	T
0.6668359953026977	0.3331563612709029	0.3224711682072439	T	T	T
0.6249999638537531	0.3749999316845773	0.0000000000000000	F	F	F
0.6666667785437201	0.4583333610645113	0.0784850000000006	F	F	F
0.7083332589907414	0.4166666463745443	0.1569700000000012	F	F	F
0.6253709486554911	0.3750877292224013	0.2384801308285820	T	T	T
0.6667360712479519	0.4583025977694514	0.3223812701519028	T	T	T
0.6250000980967059	0.5000000757544782	0.0000000000000000	F	F	F
0.6666668127866728	0.5833335051344122	0.0784850000000006	F	F	F
0.7083333932336870	0.5416667904444452	0.1569700000000012	F	F	F
0.6253333644491216	0.5002063682305344	0.2385134257087199	T	T	T
0.6668007231445175	0.5835215540081434	0.3225134110314438	T	T	T
0.6250002323396515	0.6250002198243791	0.0000000000000000	F	F	F
0.6666666967670594	0.7083331486791877	0.0784850000000006	F	F	F
0.7083332772140736	0.6666664339892208	0.1569700000000012	F	F	F
0.6250172012308407	0.6250320220409956	0.2386565605395983	T	T	T
0.6666445184652100	0.7082792793467410	0.3225046775736560	T	T	T
0.6250001163200380	0.7499998633691547	0.0000000000000000	F	F	F
0.6666668310100050	0.8333332927490886	0.0784850000000006	F	F	F
0.7083334114570263	0.7916665780591217	0.1569700000000012	F	F	F
0.6249765815192042	0.7499453249329884	0.2386538283307443	T	T	T
0.6666245547519978	0.8332509707038198	0.3224182801699977	T	T	T
0.6249998170955351	0.8750000074390556	0.0000000000000000	F	F	F
0.6666665317855021	0.9583334368189895	0.0784850000000006	F	F	F
0.7083335456999720	0.9166667221290226	0.1569700000000012	F	F	F
0.6249852426311810	0.8749614322588938	0.2385938917009079	T	T	T
0.6666325147358365	0.9582642538281032	0.3224013350763896	T	T	T
0.7500001204389335	0.0000000000000000	0.0000000000000000	F	F	F
0.7916668351289005	0.0833334293799339	0.0784850000000006	F	F	F
0.8333334155759147	0.0416667146899670	0.1569700000000012	F	F	F
0.7499798495884779	0.9999692960860271	0.2385843479602611	T	T	T

0.7916454629023799	0.0832861544051448	0.3223689862856272	T	T	T
0.7500002546818791	0.1250001440699009	0.0000000000000000	F	F	F
0.7916669693718461	0.2083335734498348	0.0784850000000006	F	F	F
0.8333335498188674	0.1666668587598679	0.1569700000000012	F	F	F
0.7499706033174121	0.1249997525105022	0.2385915965601133	T	T	T
0.7916577173961935	0.2083070947224146	0.3224249323937683	T	T	T
0.7499997051948100	0.2499997876146764	0.0000000000000000	F	F	F
0.7916664198847769	0.3333332169946104	0.0784850000000006	F	F	F
0.8333334337992468	0.2916665023046434	0.1569700000000012	F	F	F
0.7499853996142662	0.2499863138087894	0.2386580505721557	T	T	T
0.7917218066731482	0.3332985096885442	0.3225246403795908	T	T	T
0.7499998394377627	0.3749999316845773	0.0000000000000000	F	F	F
0.7916665541277297	0.4583333610645113	0.0784850000000006	F	F	F
0.8333331345747439	0.4166666463745443	0.1569700000000012	F	F	F
0.7501136414167450	0.3749641530520822	0.2386413602975517	T	T	T
0.7915431507270927	0.4582010722074439	0.3225013302611439	T	T	T
0.7499999736807084	0.5000000757544782	0.0000000000000000	F	F	F
0.7916666883706753	0.5833335051344122	0.0784850000000006	F	F	F
0.8333332688176966	0.5416667904444452	0.1569700000000012	F	F	F
0.7502258225813684	0.5000946604520384	0.2386737505557089	T	T	T
0.7915318271464153	0.5832428792010855	0.3225154096495851	T	T	T
0.7500001079236540	0.6250002198243791	0.0000000000000000	F	F	F
0.7916665723510619	0.7083331486791877	0.0784850000000006	F	F	F
0.8333331527980832	0.6666664339892208	0.1569700000000012	F	F	F
0.7500811532413164	0.6250802581325574	0.2386485117223376	T	T	T
0.7916928064783828	0.7083083273508261	0.3225194005448566	T	T	T
0.7499999919040405	0.7499998633691547	0.0000000000000000	F	F	F
0.7916667065940075	0.8333332927490886	0.0784850000000006	F	F	F
0.8333332870410288	0.7916665780591217	0.1569700000000012	F	F	F
0.7499741470719936	0.7499446889668762	0.2386541818520551	T	T	T
0.7916389583068347	0.8332495541433542	0.3224179192275117	T	T	T
0.7500001261469933	0.8750000074390556	0.0000000000000000	F	F	F
0.7916668408369603	0.9583334368189895	0.0784850000000006	F	F	F
0.8333334212839745	0.9166667221290226	0.1569700000000012	F	F	F
0.7499660707096407	0.8749269779608648	0.2385894505952614	T	T	T
0.7916386801462754	0.9582635836367104	0.3223672871857532	T	T	T
0.8749999960229360	0.0000000000000000	0.0000000000000000	F	F	F
0.9166667107129030	0.0833334293799339	0.0784850000000006	F	F	F
0.9583332911599243	0.0416667146899670	0.1569700000000012	F	F	F
0.8749954430140224	0.9999669012053437	0.2385843910292722	T	T	T
0.9166496968268557	0.0832853073202679	0.3223692508558135	T	T	T
0.8750001302658816	0.1250001440699009	0.0000000000000000	F	F	F
0.9166668449558486	0.2083335734498348	0.0784850000000006	F	F	F
0.9583334254028699	0.1666668587598679	0.1569700000000012	F	F	F
0.8749982826716517	0.1249920280521514	0.2385853778949522	T	T	T
0.9166543654757493	0.2083052925693252	0.3224078591084090	T	T	T
0.8750000142462682	0.2499997876146764	0.0000000000000000	F	F	F
0.9166667289362351	0.3333332169946104	0.0784850000000006	F	F	F
0.9583333093832564	0.2916665023046434	0.1569700000000012	F	F	F
0.8749890553397877	0.2499854753587438	0.2385966174881837	T	T	T
0.9166736502930153	0.3333073401868595	0.3224788401396815	T	T	T
0.8750001484892138	0.3749999316845773	0.0000000000000000	F	F	F
0.9166668631791808	0.4583333610645113	0.0784850000000006	F	F	F
0.9583334436262021	0.4166666463745443	0.1569700000000012	F	F	F
0.8749960892781360	0.3749446972541485	0.2386205241925328	T	T	T
0.9166039226800025	0.4582700583890683	0.3224576984826664	T	T	T
0.8749998492647180	0.5000000757544782	0.0000000000000000	F	F	F
0.9166665639546849	0.5833335051344122	0.0784850000000006	F	F	F
0.9583335778691477	0.5416667904444452	0.1569700000000012	F	F	F
0.8750029100657186	0.4999406645068462	0.2386448888282766	T	T	T
0.9165197718113552	0.5832215528874835	0.3223730251972791	T	T	T
0.8749999835076636	0.6250002198243791	0.0000000000000000	F	F	F
0.9166664479350644	0.7083331486791877	0.0784850000000006	F	F	F
0.9583330283820857	0.6666664339892208	0.1569700000000012	F	F	F
0.8749913713796974	0.6250202043587818	0.2386465165028659	T	T	T
0.9165862451648362	0.7082423301062355	0.3224668461986188	T	T	T
0.8749998674880501	0.7499998633691547	0.0000000000000000	F	F	F
0.9166665821780171	0.8333332927490886	0.0784850000000006	F	F	F
0.9583331626250313	0.7916665780591217	0.1569700000000012	F	F	F
0.8749817186335743	0.7499976561640089	0.2386193019984981	T	T	T
0.9166491978870305	0.8332669288217748	0.3224746275670795	T	T	T
0.8750000017309958	0.8750000074390556	0.0000000000000000	F	F	F
0.9166667164209628	0.9583334368189895	0.0784850000000006	F	F	F
0.9583332968679841	0.9166667221290226	0.1569700000000012	F	F	F
0.8749816989363348	0.8749586328016683	0.2385947102335156	T	T	T

0.9166448467533339	0.9582622185144237	0.3224004847661802	T	T	T
0.5716587584356244	0.4086172469535312	0.4241554279722849	T	T	T
0.4620837250901053	0.4087602140603047	0.4242265105772831	T	T	T
0.4619341485796450	0.2990265996397628	0.4238533553511000	T	T	T
0.5339750122880138	0.4428291958729677	0.4243581030104645	T	T	T
0.5338324555621179	0.3367041706371747	0.4239600596677501	T	T	T
0.4278338547399596	0.3368377634167416	0.4240277476002480	T	T	T
0.6317259677242532	0.4385714798910569	0.4243733453252159	T	T	T
0.5593070801519822	0.3110914005567999	0.4238497421610201	T	T	T
0.4318745981920398	0.2389582929857517	0.4238718962444564	T	T	T
0.3767441379906203	0.3113501120484065	0.4239647845522651	T	T	T
0.4321068618055675	0.4388394627487077	0.4245079486929409	T	T	T
0.5595563768487420	0.4939110222859481	0.4245503182880319	T	T	T
0.6180822153771982	0.6111594588822625	0.4179383884969931	T	T	T
0.6167468200925217	0.6084434359478107	0.4430120614253956	T	T	T
0.6199588234925237	0.2539921757000119	0.4177932284497246	T	T	T
0.6183113595732712	0.2551084037931016	0.4428643111838491	T	T	T
0.2592638682336573	0.2544228870802644	0.4178233643687935	T	T	T
0.2621827952590328	0.2557297788109338	0.4428873853418386	T	T	T
0.2928753752641063	0.4020269351997191	0.4180803409736860	T	T	T
0.2964403901010572	0.4035910818483168	0.4430754497816838	T	T	T
0.2914089416974506	0.1396832755569772	0.4181209236655218	T	T	T
0.2950829260315558	0.1416117629676397	0.4431095707949091	T	T	T
0.4712576443624289	0.5782638797052918	0.4180243413582165	T	T	T
0.4697678961848356	0.5746972225836782	0.4430189518820618	T	T	T
0.4732312098647004	0.1397098318295335	0.4180790777001792	T	T	T
0.4715607010223574	0.1417834907080397	0.4430625837772368	T	T	T
0.7337559700307302	0.4010572760633167	0.4180022352759399	T	T	T
0.7316378200499509	0.4024304209932854	0.4430012218030757	T	T	T
0.7319777143075241	0.5781577576873939	0.4180123934213189	T	T	T
0.7298271648275928	0.5745434015001981	0.4430033550111311	T	T	T

Borazine dimer adsorbed on Au(111), dimer II, with 10 H2 molecules

23.0697829620000014	0.0000000000000000	0.0000000000000000
-11.5348914810000007	19.9790169730000002	0.0000000000000000
0.0000000000000000	0.0000000000000000	30.0000000000000000

Au B N H
320 6 6 32
Selective dynamics
Direct

0.0000000000000000	0.0000000000000000	0.0000000000000000	F	F	F
0.0416667146899670	0.0833334293799339	0.0784850000000006	F	F	F
0.0833332951369883	0.0416667146899670	0.1569700000000012	F	F	F
0.0000537259102898	0.0000337061172669	0.2385736472189307	T	T	T
0.0416603928195830	0.0833134677777821	0.3224596141669762	T	T	T
0.0000001342429456	0.1250001440699009	0.0000000000000000	F	F	F
0.0416668489329126	0.2083335734498348	0.0784850000000006	F	F	F
0.0833334293799339	0.1666668587598679	0.1569700000000012	F	F	F
0.0000241927953762	0.1250143488915853	0.2385897815480675	T	T	T
0.0416592827734681	0.2083279096828799	0.3225046551897596	T	T	T
0.0000000182233322	0.2499997876146764	0.0000000000000000	F	F	F
0.0416667329132991	0.3333332169946104	0.0784850000000006	F	F	F
0.0833333133603205	0.2916665023046434	0.1569700000000012	F	F	F
0.0000431010417638	0.2500036662687813	0.2386001216882904	T	T	T
0.0417255064910459	0.3333687595853942	0.3223690432462044	T	T	T
0.0000001524662778	0.3749999316845773	0.0000000000000000	F	F	F
0.0416668671562448	0.4583333610645113	0.0784850000000006	F	F	F
0.0833334476032661	0.4166666463745443	0.1569700000000012	F	F	F
0.0000443099743128	0.3749915184566292	0.2385815096901640	T	T	T
0.0417414133967063	0.4583360542918527	0.3224549776348462	T	T	T
0.9999997248487205	0.5000000757544782	0.0000000000000000	F	F	F
0.0416665679317489	0.5833335051344122	0.0784850000000006	F	F	F
0.0833335818462118	0.5416667904444452	0.1569700000000012	F	F	F
0.0000535902490299	0.4999870019828954	0.2385781160668842	T	T	T
0.0417469714018403	0.5833329880065797	0.3224191260705314	T	T	T
0.9999998590916661	0.6250002198243791	0.0000000000000000	F	F	F
0.0416664519121284	0.7083331486791877	0.0784850000000006	F	F	F
0.0833330323591497	0.6666664339892208	0.1569700000000012	F	F	F
0.0000533426413836	0.6250053021029535	0.2385688185644825	T	T	T
0.0418063497780608	0.7083454844421245	0.3223855419658604	T	T	T
0.9999997430720526	0.7499998633691547	0.0000000000000000	F	F	F
0.0416665861550811	0.8333332927490886	0.0784850000000006	F	F	F
0.0833331666020953	0.7916665780591217	0.1569700000000012	F	F	F
0.0000439306827922	0.7499974803496965	0.2385657976354666	T	T	T
0.0417608779594616	0.8333324820589488	0.3223861764220814	T	T	T

0.0000000057080598	0.8750000074390556	0.0000000000000000	F	F	F
0.0416667203980268	0.9583334368189895	0.0784850000000006	F	F	F
0.0833333008450481	0.9166667221290226	0.1569700000000012	F	F	F
0.0000360190559462	0.8750272433558284	0.2385691272108438	T	T	T
0.0417208871415859	0.9583192415118661	0.3224122269942646	T	T	T
0.1249998755840025	0.0000000000000000	0.0000000000000000	F	F	F
0.1666665902739695	0.0833334293799339	0.0784850000000006	F	F	F
0.2083331707209908	0.0416667146899670	0.1569700000000012	F	F	F
0.1250370095693387	0.0000174717402583	0.2386181776040576	T	T	T
0.1666394943699434	0.0832978469785353	0.3224937742178282	T	T	T
0.1250000098269552	0.1250001440699009	0.0000000000000000	F	F	F
0.1666667245169222	0.2083335734498348	0.0784850000000006	F	F	F
0.2083333049639364	0.1666668587598679	0.1569700000000012	F	F	F
0.1249872848184486	0.1249988781746724	0.2386662328106949	T	T	T
0.1665182916038495	0.2082762409334075	0.3226430647757285	T	T	T
0.1249998938073347	0.2499997876146764	0.0000000000000000	F	F	F
0.1666666084973016	0.3333332169946104	0.0784850000000006	F	F	F
0.2083331889443230	0.2916665023046434	0.1569700000000012	F	F	F
0.1249711792932841	0.2499590032241031	0.2386519346619407	T	T	T
0.1666448590505689	0.3332596464632445	0.3225319736609839	T	T	T
0.1250000280502874	0.3749999316845773	0.0000000000000000	F	F	F
0.1666667427402544	0.4583333610645113	0.0784850000000006	F	F	F
0.2083333231872686	0.4166666463745443	0.1569700000000012	F	F	F
0.1250515335563242	0.3749983549532425	0.2386554681862490	T	T	T
0.1667317803221489	0.4583381420046051	0.3225566543557503	T	T	T
0.1250001622932331	0.5000000757544782	0.0000000000000000	F	F	F
0.1666668769832000	0.5833335051344122	0.0784850000000006	F	F	F
0.2083334574302214	0.5416667904444452	0.1569700000000012	F	F	F
0.1250222923753512	0.4999580347143096	0.2386258163091978	T	T	T
0.1667284600963333	0.5832946083863887	0.3224685093084451	T	T	T
0.1250002965361787	0.6250002198243791	0.0000000000000000	F	F	F
0.1666667609635866	0.7083331486791877	0.0784850000000006	F	F	F
0.2083333414106079	0.6666664339892208	0.1569700000000012	F	F	F
0.1250256461670656	0.6249699578499347	0.2385932843216596	T	T	T
0.1668086568558568	0.7083511835502325	0.3223663096095525	T	T	T
0.1249997470491166	0.7499998633691547	0.0000000000000000	F	F	F
0.1666664617390836	0.8333332927490886	0.0784850000000006	F	F	F
0.2083334756535535	0.7916665780591217	0.1569700000000012	F	F	F
0.1250509077813027	0.7500095798411854	0.2385552647157595	T	T	T
0.1668174457950435	0.8333880918244395	0.3223913002774593	T	T	T
0.1249998812920623	0.8750000074390556	0.0000000000000000	F	F	F
0.1666665959820293	0.9583334368189895	0.0784850000000006	F	F	F
0.2083331764290506	0.9166667221290226	0.1569700000000012	F	F	F
0.1250284414384063	0.8750188070861188	0.2385734045469895	T	T	T
0.1667093548401243	0.9582845706224515	0.3224793733550400	T	T	T
0.2500001846354607	0.0000000000000000	0.0000000000000000	F	F	F
0.2916668993254277	0.0833334293799339	0.0784850000000006	F	F	F
0.3333334797724490	0.0416667146899670	0.1569700000000012	F	F	F
0.2499549655667978	0.9999283512305650	0.2386113699175641	T	T	T
0.2914875427868485	0.0831929648431144	0.3225556572228452	T	T	T
0.2499998854109577	0.1250001440699009	0.0000000000000000	F	F	F
0.2916666001009247	0.2083335734498348	0.0784850000000006	F	F	F
0.3333336140153946	0.1666668587598679	0.1569700000000012	F	F	F
0.2499271787267835	0.1249146216647411	0.2387107313486918	T	T	T
0.2913673028382369	0.2082417277529130	0.3225715015519427	T	T	T
0.2499997693913443	0.2499997876146764	0.0000000000000000	F	F	F
0.2916664840813112	0.3333332169946104	0.0784850000000006	F	F	F
0.3333330645283255	0.2916665023046434	0.1569700000000012	F	F	F
0.2497211809626771	0.2497794017590265	0.2386330012433708	T	T	T
0.2914092573034348	0.3329884012674882	0.3225703883284903	T	T	T
0.2499999036342899	0.3749999316845773	0.0000000000000000	F	F	F
0.2916666183242569	0.4583333610645113	0.0784850000000006	F	F	F
0.3333331987712782	0.4166666463745443	0.1569700000000012	F	F	F
0.2498479279816614	0.3748742147260059	0.2387171982772860	T	T	T
0.2915142081634615	0.4581523475658494	0.3224377756233763	T	T	T
0.2500000378772356	0.5000000757544782	0.0000000000000000	F	F	F
0.2916667525672025	0.5833335051344122	0.0784850000000006	F	F	F
0.3333333330142239	0.5416667904444452	0.1569700000000012	F	F	F
0.2499166485427648	0.4999934561783893	0.2386758822234151	T	T	T
0.2916424983255976	0.5832947663726394	0.3225575927771299	T	T	T
0.2500001721201883	0.6250002198243791	0.0000000000000000	F	F	F
0.2916666365475891	0.7083331486791877	0.0784850000000006	F	F	F
0.3333332169946104	0.6666664339892208	0.1569700000000012	F	F	F
0.2499632537757901	0.6250147999237755	0.2386436021648267	T	T	T
0.2918057880292846	0.7083167823564438	0.3224216714525129	T	T	T

0.2500000561005749	0.7499998633691547	0.0000000000000000	F	F	F
0.2916667707905418	0.8333332927490886	0.0784850000000006	F	F	F
0.3333333512375560	0.7916665780591217	0.1569700000000012	F	F	F
0.2500697555014383	0.7500223324711105	0.2386003198203032	T	T	T
0.2918398358800405	0.8334243167159047	0.3223103169081480	T	T	T
0.2500001903435205	0.8750000074390556	0.0000000000000000	F	F	F
0.2916669050334875	0.9583334368189895	0.0784850000000006	F	F	F
0.3333334854805088	0.9166667221290226	0.1569700000000012	F	F	F
0.2500520882763219	0.8750350150891272	0.2386178182299284	T	T	T
0.2918025045353179	0.9584693016928262	0.3224889247102914	T	T	T
0.3750000602194632	0.0000000000000000	0.0000000000000000	F	F	F
0.4166667749094302	0.0833334293799339	0.0784850000000006	F	F	F
0.4583333553564515	0.0416667146899670	0.1569700000000012	F	F	F
0.3749958188420949	0.9998619878157392	0.2386867620696790	T	T	T
0.4167054389791185	0.0833429981639519	0.3226200891592658	T	T	T
0.3750001944624159	0.1250001440699009	0.0000000000000000	F	F	F
0.4166669091523829	0.2083335734498348	0.0784850000000006	F	F	F
0.4583334895993971	0.1666668587598679	0.1569700000000012	F	F	F
0.3748276324093565	0.1246235042288826	0.2385427553863062	T	T	T
0.4161829785891512	0.2080437427587666	0.3212348267680657	T	T	T
0.3750000784427954	0.2499997876146764	0.0000000000000000	F	F	F
0.4166667931327623	0.3333332169946104	0.0784850000000006	F	F	F
0.4583333735797837	0.2916665023046434	0.1569700000000012	F	F	F
0.3747074316736331	0.2498348019738290	0.2384869228772683	T	T	T
0.4164908303279060	0.3331509189276436	0.3221977877501157	T	T	T
0.3749997792182995	0.3749999316845773	0.0000000000000000	F	F	F
0.4166664939082665	0.4583333610645113	0.0784850000000006	F	F	F
0.4583335078227293	0.4166666463745443	0.1569700000000012	F	F	F
0.3745613716289756	0.3745809537904126	0.2383681884459332	T	T	T
0.4164747488556736	0.4577520328949286	0.3207928899390536	T	T	T
0.3749999134612452	0.5000000757544782	0.0000000000000000	F	F	F
0.4166666281512121	0.5833335051344122	0.0784850000000006	F	F	F
0.4583332085982335	0.5416667904444452	0.1569700000000012	F	F	F
0.3748119113488544	0.5000174712496086	0.2385039471457722	T	T	T
0.4165509707954384	0.5834780871724923	0.3214811255939780	T	T	T
0.3750000477041908	0.6250002198243791	0.0000000000000000	F	F	F
0.4166665121315987	0.7083331486791877	0.0784850000000006	F	F	F
0.4583330925786129	0.6666664339892208	0.1569700000000012	F	F	F
0.3748506855284417	0.6252214638299324	0.2385397963642453	T	T	T
0.4167023577269330	0.7083857191317517	0.3225974441834371	T	T	T
0.3749999316845773	0.7499998633691547	0.0000000000000000	F	F	F
0.4166666463745443	0.8333332927490886	0.0784850000000006	F	F	F
0.4583332268215656	0.7916665780591217	0.1569700000000012	F	F	F
0.3750734261307122	0.7501174207830830	0.2386456819861152	T	T	T
0.4167733593222854	0.8333860372304679	0.3225679962825770	T	T	T
0.3750000659275230	0.8750000074390556	0.0000000000000000	F	F	F
0.4166667806174900	0.9583334368189895	0.0784850000000006	F	F	F
0.4583333610645113	0.9166667221290226	0.1569700000000012	F	F	F
0.3751331727659361	0.8750454278177487	0.2386363326802338	T	T	T
0.4168163270405514	0.9584718256957057	0.3224831135504435	T	T	T
0.4999999358034728	0.0000000000000000	0.0000000000000000	F	F	F
0.5416666504934398	0.0833334293799339	0.0784850000000006	F	F	F
0.5833332309404540	0.0416667146899670	0.1569700000000012	F	F	F
0.5000817365104198	0.9999629507279274	0.2386435903454424	T	T	T
0.5417523985101972	0.0832651809995597	0.3225955903582670	T	T	T
0.5000000700464184	0.1250001440699009	0.0000000000000000	F	F	F
0.5416667847363854	0.2083335734498348	0.0784850000000006	F	F	F
0.5833333651834067	0.1666668587598679	0.1569700000000012	F	F	F
0.4999334712449094	0.1247267333749304	0.2385865278889341	T	T	T
0.5419088042881267	0.2081776432871122	0.3214705554468354	T	T	T
0.4999999540268050	0.2499997876146764	0.0000000000000000	F	F	F
0.5416666687167719	0.3333332169946104	0.0784850000000006	F	F	F
0.5833332491637861	0.2916665023046434	0.1569700000000012	F	F	F
0.5000108323610721	0.2499152843560565	0.2385089030917823	T	T	T
0.5419997637143348	0.3339477877853946	0.3207774997170044	T	T	T
0.5000000882697506	0.3749999316845773	0.0000000000000000	F	F	F
0.5416668029597176	0.4583333610645113	0.0784850000000006	F	F	F
0.5833333834067389	0.4166666463745443	0.1569700000000012	F	F	F
0.4998634477824988	0.3751036581031177	0.2383807879058949	T	T	T
0.5420438264741484	0.4585688802037879	0.3221742144771442	T	T	T
0.5000002225126963	0.5000000757544782	0.0000000000000000	F	F	F
0.5416669372026632	0.5833335051344122	0.0784850000000006	F	F	F
0.5833335176496846	0.5416667904444452	0.1569700000000012	F	F	F
0.5001640446128854	0.5000736552267653	0.2383829190080163	T	T	T
0.5422324495512562	0.5836990965019891	0.3210982102715738	T	T	T

0.4999999232882004	0.6250002198243791	0.0000000000000000	F	F	F
0.5416663877156012	0.7083331486791877	0.0784850000000006	F	F	F
0.5833334016300711	0.6666664339892208	0.1569700000000012	F	F	F
0.5000987350076092	0.6252274616370227	0.2385152247432549	T	T	T
0.5417871118815522	0.7084915796580247	0.3226007060053346	T	T	T
0.4999998072685798	0.7499998633691547	0.0000000000000000	F	F	F
0.5416665219585468	0.8333332927490886	0.0784850000000006	F	F	F
0.5833331024055681	0.7916665780591217	0.1569700000000012	F	F	F
0.5000363657461655	0.7500791915778003	0.2387006161481062	T	T	T
0.5416388820539660	0.8332741583089026	0.3225575613668102	T	T	T
0.4999999415115326	0.8750000074390556	0.0000000000000000	F	F	F
0.5416666562014996	0.9583334368189895	0.0784850000000006	F	F	F
0.5833332366485138	0.9166667221290226	0.1569700000000012	F	F	F
0.5000596714732939	0.8750085037698998	0.2386459332333224	T	T	T
0.5416565859946683	0.9582829223292243	0.3225777225687272	T	T	T
0.6249998113874753	0.0000000000000000	0.0000000000000000	F	F	F
0.6666665260774423	0.0833334293799339	0.0784850000000006	F	F	F
0.7083335399919122	0.0416667146899670	0.1569700000000012	F	F	F
0.6249844218354575	0.9999829664007099	0.2386442345936521	T	T	T
0.6666583863516180	0.0833195007635766	0.3224557830876876	T	T	T
0.6249999456304209	0.1250001440699009	0.0000000000000000	F	F	F
0.6666666603203879	0.2083335734498348	0.0784850000000006	F	F	F
0.7083332407674092	0.1666668587598679	0.1569700000000012	F	F	F
0.6250260701006959	0.1250075884851979	0.2386835943407801	T	T	T
0.6668093723114650	0.2082961465498620	0.3225980659711267	T	T	T
0.6249998296108075	0.2499997876146764	0.0000000000000000	F	F	F
0.6666665443007744	0.3333332169946104	0.0784850000000006	F	F	F
0.7083331247477958	0.2916665023046434	0.1569700000000012	F	F	F
0.6252814097830927	0.2501204110193900	0.2385736293055757	T	T	T
0.6668515147146096	0.3334653208059339	0.3223515647858669	T	T	T
0.6249999638537531	0.3749999316845773	0.0000000000000000	F	F	F
0.6666666785437201	0.4583333610645113	0.0784850000000006	F	F	F
0.7083332589907414	0.4166666463745443	0.1569700000000012	F	F	F
0.6254366598242620	0.3753235015546285	0.2384884935134847	T	T	T
0.6670896538247231	0.4587545782839704	0.3226130591140199	T	T	T
0.6250000980967059	0.5000000757544782	0.0000000000000000	F	F	F
0.6666668127866728	0.5833335051344122	0.0784850000000006	F	F	F
0.7083333932336870	0.5416667904444452	0.1569700000000012	F	F	F
0.6252793723351907	0.5001392447554870	0.2386141866942618	T	T	T
0.6670361808349148	0.5834757459059485	0.3225381945222802	T	T	T
0.6250002323396515	0.6250002198243791	0.0000000000000000	F	F	F
0.6666666967670594	0.7083331486791877	0.0784850000000006	F	F	F
0.7083332772140736	0.6666664339892208	0.1569700000000012	F	F	F
0.6255257957905777	0.6252611661879126	0.2385002857088656	T	T	T
0.6669325557893803	0.7085441199084572	0.3225494866357577	T	T	T
0.6250001163200380	0.7499998633691547	0.0000000000000000	F	F	F
0.6666668310100050	0.8333332927490886	0.0784850000000006	F	F	F
0.7083334114570263	0.7916665780591217	0.1569700000000012	F	F	F
0.6250855995430407	0.7500660683095971	0.2386944264200663	T	T	T
0.6666982393242883	0.8333144667709395	0.3225311071248069	T	T	T
0.6249998170955351	0.8750000074390556	0.0000000000000000	F	F	F
0.6666665317855021	0.9583334368189895	0.0784850000000006	F	F	F
0.7083335456999720	0.9166667221290226	0.1569700000000012	F	F	F
0.6249831403530044	0.8749422883251621	0.2386807264009002	T	T	T
0.6666360551639603	0.9582882669516211	0.3223759446754131	T	T	T
0.7500001204389335	0.0000000000000000	0.0000000000000000	F	F	F
0.7916668351289005	0.0833334293799339	0.0784850000000006	F	F	F
0.8333334155759147	0.0416667146899670	0.1569700000000012	F	F	F
0.7499758157144804	0.9999857851683459	0.2385922494396449	T	T	T
0.7916741529185726	0.0833344829135054	0.3224226495159046	T	T	T
0.7500002546818791	0.1250001440699009	0.0000000000000000	F	F	F
0.7916669693718461	0.2083335734498348	0.0784850000000006	F	F	F
0.8333335498188674	0.1666668587598679	0.1569700000000012	F	F	F
0.7499897490270768	0.1250147826624752	0.2386533680106944	T	T	T
0.7917381637382447	0.2083739058284876	0.3225228889951727	T	T	T
0.7499997051948100	0.2499997876146764	0.0000000000000000	F	F	F
0.7916664198847769	0.3333332169946104	0.0784850000000006	F	F	F
0.8333334337992468	0.2916665023046434	0.1569700000000012	F	F	F
0.7500631483058126	0.2499840887853807	0.2386483059687942	T	T	T
0.7916473835607136	0.3332856136671434	0.3225280648336018	T	T	T
0.7499998394377627	0.3749999316845773	0.0000000000000000	F	F	F
0.7916665541277297	0.4583333610645113	0.0784850000000006	F	F	F
0.8333331345747439	0.4166666463745443	0.1569700000000012	F	F	F
0.7502556362651478	0.3751313762847115	0.2386732875431348	T	T	T
0.7917354327405063	0.4583740482582337	0.3225415715521098	T	T	T

0.7499999736807084	0.5000000757544782	0.0000000000000000	F	F	F
0.7916666883706753	0.5833335051344122	0.0784850000000006	F	F	F
0.8333332688176966	0.5416667904444452	0.1569700000000012	F	F	F
0.7502258996562243	0.5001568498666407	0.2386727300237055	T	T	T
0.7919250255251152	0.5834185003558230	0.3226902471717565	T	T	T
0.7500001079236540	0.6250002198243791	0.0000000000000000	F	F	F
0.7916665723510619	0.7083331486791877	0.0784850000000006	F	F	F
0.8333331527980832	0.6666664339892208	0.1569700000000012	F	F	F
0.7502626941849085	0.6250549596936715	0.2386992533947668	T	T	T
0.7917600613781453	0.7083632169699193	0.3224957544581211	T	T	T
0.7499999919040405	0.7499998633691547	0.0000000000000000	F	F	F
0.7916667065940075	0.8333332927490886	0.0784850000000006	F	F	F
0.8333332870410288	0.7916665780591217	0.1569700000000012	F	F	F
0.7501678127963555	0.7501049661615820	0.2386422801788372	T	T	T
0.7917483607904072	0.8333971651361550	0.3225297171359561	T	T	T
0.7500001261469933	0.8750000074390556	0.0000000000000000	F	F	F
0.7916668408369603	0.9583334368189895	0.0784850000000006	F	F	F
0.8333334212839745	0.9166667221290226	0.1569700000000012	F	F	F
0.7499977015846775	0.8749846058295161	0.2386364497145850	T	T	T
0.7916716000158540	0.9583203185929220	0.3224087604802929	T	T	T
0.8749999960229360	0.0000000000000000	0.0000000000000000	F	F	F
0.9166667107129030	0.0833334293799339	0.0784850000000006	F	F	F
0.9583332911599243	0.0416667146899670	0.1569700000000012	F	F	F
0.8750114694725731	0.0000098063009635	0.2385800656052103	T	T	T
0.9166800482421555	0.0833430224754086	0.3224159847418328	T	T	T
0.8750001302658816	0.1250001440699009	0.0000000000000000	F	F	F
0.9166668449558486	0.2083335734498348	0.0784850000000006	F	F	F
0.9583334254028699	0.1666668587598679	0.1569700000000012	F	F	F
0.8750057192570697	0.1250031133198103	0.2385945483188291	T	T	T
0.9167138838541110	0.2083447260752571	0.3224246773549995	T	T	T
0.8750000142462682	0.2499997876146764	0.0000000000000000	F	F	F
0.9166667289362351	0.3333332169946104	0.0784850000000006	F	F	F
0.9583333093832564	0.2916665023046434	0.1569700000000012	F	F	F
0.8750093198322934	0.2499789865113861	0.2386043216580498	T	T	T
0.9167006203698241	0.3333442147497119	0.3224494191855155	T	T	T
0.8750001484892138	0.3749999316845773	0.0000000000000000	F	F	F
0.9166668631791808	0.4583333610645113	0.0784850000000006	F	F	F
0.9583334436262021	0.4166666463745443	0.1569700000000012	F	F	F
0.8750292133309290	0.3749526331932375	0.2386447490414327	T	T	T
0.9166882083336205	0.4582961534985793	0.3223328567760397	T	T	T
0.8749998492647180	0.5000000757544782	0.0000000000000000	F	F	F
0.9166665639546849	0.5833335051344122	0.0784850000000006	F	F	F
0.9583335778691477	0.5416667904444452	0.1569700000000012	F	F	F
0.8750859375870123	0.5000526451473434	0.2386417085201728	T	T	T
0.9167906437205754	0.5833421330457742	0.3225071949667740	T	T	T
0.8749999835076636	0.6250002198243791	0.0000000000000000	F	F	F
0.9166664479350644	0.7083331486791877	0.0784850000000006	F	F	F
0.9583330283820857	0.6666664339892208	0.1569700000000012	F	F	F
0.8750778081583985	0.6250010386353912	0.2386410678275095	T	T	T
0.9167997784179639	0.7083636117445919	0.3224358254686403	T	T	T
0.8749998674880501	0.7499998633691547	0.0000000000000000	F	F	F
0.9166665821780171	0.8333332927490886	0.0784850000000006	F	F	F
0.9583331626250313	0.7916665780591217	0.1569700000000012	F	F	F
0.8750696017325210	0.7500203383924694	0.2386312620053174	T	T	T
0.9167294085589205	0.8333469788930427	0.3224167510842889	T	T	T
0.8750000017309958	0.8750000074390556	0.0000000000000000	F	F	F
0.9166667164209628	0.9583334368189895	0.0784850000000006	F	F	F
0.9583332968679841	0.9166667221290226	0.1569700000000012	F	F	F
0.8750282325673407	0.8750307695727766	0.2386063141421586	T	T	T
0.9167214247149732	0.9583602275075792	0.3224002031009341	T	T	T
0.4972964268502853	0.4975654262766909	0.4243505114879735	T	T	T
0.4962548334508250	0.6065495963723910	0.4258867963891440	T	T	T
0.3871892726969385	0.4963850499578337	0.4251098701885472	T	T	T
0.4655137874216020	0.1867661691495424	0.4260073232411457	T	T	T
0.5741731138824400	0.2973306971559495	0.4247041802676483	T	T	T
0.4636275061386788	0.2953218745519908	0.4244062025782427	T	T	T
0.5310024463933261	0.5693446786380978	0.4251817715100951	T	T	T
0.4254671644010728	0.4627437914593404	0.4243887361671461	T	T	T
0.4244198273634473	0.5682393685937415	0.4260610973103923	T	T	T
0.5373437797548460	0.2254696881455332	0.4258313502327125	T	T	T
0.4303722651046031	0.2235780001621056	0.4254476219015003	T	T	T
0.5354815896564766	0.3305515634475976	0.4240958849379865	T	T	T
0.5278996030387826	0.4679988640007831	0.4239635439557018	T	T	T
0.4003774633600182	0.4116761276309831	0.4235352651421290	T	T	T
0.3270966117917238	0.4658190100804878	0.4251476349780892	T	T	T

0.3985133434232391	0.5933967852605555	0.4265596191209314	T	T	T
0.5258926797041149	0.6666453331382221	0.4265186037532098	T	T	T
0.5820755886512875	0.5952421324202857	0.4249133344893091	T	T	T
0.4362450039466533	0.1266935644666819	0.4268193332396880	T	T	T
0.3793082719208806	0.1973908467075799	0.4254625249770170	T	T	T
0.4327145794991284	0.3245610099978293	0.4241427268465627	T	T	T
0.5602608183237316	0.3816045458853978	0.4230970713575253	T	T	T
0.6342727379111113	0.3282357575501724	0.4245392012225423	T	T	T
0.5635045261559524	0.2005703197428944	0.4262357808509309	T	T	T
0.6103672416103605	0.1314642516363082	0.4193777728560710	T	T	T
0.6088973918427310	0.1324265912073861	0.4444750872163604	T	T	T
0.7799703161875616	0.3865065572350865	0.4182163821181271	T	T	T
0.7776172136106931	0.3853981864993188	0.4432800791164920	T	T	T
0.3762752699198041	0.9808917594800637	0.4185741278101675	T	T	T
0.3776606834921362	0.9826409636021631	0.4436559885530351	T	T	T
0.2630344204993622	0.1402049731696489	0.4189360042556916	T	T	T
0.2658128019774156	0.1422967147301719	0.4440108311784284	T	T	T
0.2949710099411116	0.2980208221628148	0.4205807250022022	T	T	T
0.2986904598182407	0.3014799934871520	0.4455664757507059	T	T	T
0.6667879228580484	0.4998401347563703	0.4201257544045762	T	T	T
0.6629986005439984	0.4968877944042385	0.4451221150833670	T	T	T
0.6991403590708213	0.6534213749002159	0.4185881493437752	T	T	T
0.6963683441740107	0.6510925108974157	0.4436588740707940	T	T	T
0.5942392491017560	0.8135008746722456	0.4181747866907675	T	T	T
0.5934511823940863	0.8115342381188870	0.4432532646328443	T	T	T
0.3432312327364975	0.6544995884044645	0.4192051198635381	T	T	T
0.3442696542696598	0.6532250881387908	0.4443080913124947	T	T	T
0.1816211258718800	0.3952390450728983	0.4183474052413895	T	T	T
0.1842889896084333	0.3956220493846175	0.4433856579457001	T	T	T
